# Oncolytic virotherapy: basic principles, recent advances and future directions

**DOI:** 10.1038/s41392-023-01407-6

**Published:** 2023-04-11

**Authors:** Danni Lin, Yinan Shen, Tingbo Liang

**Affiliations:** 1 Department of Hepatobiliary and Pancreatic Surgery, The First Affiliated Hospital, Zhejiang University School of Medicine, Hangzhou, Zhejiang China; 2 Zhejiang Provincial Key Laboratory of Pancreatic Disease, The First Affiliated Hospital, Zhejiang University School of Medicine, Hangzhou, Zhejiang China; 3 Zhejiang Clinical Research Center of Hepatobiliary and Pancreatic Diseases, Hangzhou, Zhejiang China; 4 The Innovation Center for the Study of Pancreatic Diseases of Zhejiang Province, Hangzhou, Zhejiang China; 5 Cancer Center, Zhejiang University, Hangzhou, Zhejiang China

**Keywords:** Cancer therapy, Drug development

## Abstract

Oncolytic viruses (OVs) have attracted growing awareness in the twenty-first century, as they are generally considered to have direct oncolysis and cancer immune effects. With the progress in genetic engineering technology, OVs have been adopted as versatile platforms for developing novel antitumor strategies, used alone or in combination with other therapies. Recent studies have yielded eye-catching results that delineate the promising clinical outcomes that OVs would bring about in the future. In this review, we summarized the basic principles of OVs in terms of their classifications, as well as the recent advances in OV-modification strategies based on their characteristics, biofunctions, and cancer hallmarks. Candidate OVs are expected to be designed as “qualified soldiers” first by improving target fidelity and safety, and then equipped with “cold weapons” for a proper cytocidal effect, “hot weapons” capable of activating cancer immunotherapy, or “auxiliary weapons” by harnessing tactics such as anti-angiogenesis, reversed metabolic reprogramming and decomposing extracellular matrix around tumors. Combinations with other cancer therapeutic agents have also been elaborated to show encouraging antitumor effects. Robust results from clinical trials using OV as a treatment congruously suggested its significance in future application directions and challenges in developing OVs as novel weapons for tactical decisions in cancer treatment.

## Introduction

Viruses used to be associated with the evil devil. However, oncolytic viruses (OVs) are comparable to be noble angels, as they can save lives. Oncolytic virotherapy is an emerging novel tumor therapeutic approach that selectively replicates in and destroys tumor cells while leaving normal cells undamaged.^1,2^ Initially, in the twentieth century, investigations carried out on the oncolytic effects were generally based on wild-type or naturally occurring viruses such as West Nile virus, rabies virus, yellow fever, hepatitis, etc.,^3^ and the mechanism was simply thought to be their intrinsic lytic characteristics. Approaching the year 2000, it is technically feasible to carry out an array of modifications on wild-type viruses by means of genetic engineering. Modified OVs can be armed with desired exogenous genes that could exert profound antitumor effects via different mechanisms. At first, the main focus of reconstructions was to improve target specificity, selective replication and oncolysis. Soon later, an elicited antigen-specific antitumor immunoreactive response during tumor lysis was appreciated, which is another advantage of OVs as immunotherapy.^2^ Strategies have, therefore, begun to shift toward developing viral vectors for enhancing immune responses within the tumors, or for adjusting tumor neovascularization, tumor metabolism and other aspects to counteract the malicious tumor microenvironment (TME) in recent years. This process can be graphically described as “soldiers” equipped with a variety of “sophisticated weapons” to cope with different situations. Also, it is equally important to arm a soldier with the right weapons to maximize tumor damage.

The outcomes of OVs are determined by a three-way race among virus replication, immune activation and tumor growth.^4^ Unlike the theories of conventional chemoradiotherapy, OVs precisely lyse cancer cells by interacting with specific cellular receptors, or taking advantage of tumor-suppressor gene defects, downregulation of the antiviral pathway in tumor cells, or by designing virus vector with specific gene knockout. The benefits regarding different forms of cell death are various due to the characteristics of virus vectors and tumor cell type, and most of them can trigger immunogenic cell death (ICD), releasing tumor-associated antigens and initiating antitumor immune responses. However, it cannot be ignored that antiviral immunity can be triggered at the same time as the infection has been launched. Therefore, the selection and design of virus vectors are diversified and flexible, considering the balance among the viruses, TME and host immunity. Regarding the activation of tumor immunity, OVs seem to outperform the ICIs and other targeted drugs since ICIs specifically target the immune checkpoint, while small molecule drugs only target a certain molecule. In the context of OVs, a broader range of antitumor immunity activities would be aroused to fight against tumors. For example, the release of TAAs during oncolysis, the initiation of immunity, the promoted immune cell infiltration, improved recognition and killing abilities of immune cells, the reversal of the immunosuppressive microenvironment, and others (a detailed comparison of this part is presented in Bommareddy’s review).^5^ Compared with the limited effect of other treatment methods, OVs can carry out multiple “weapons” to kill tumors systematically and comprehensively in multiple ways. OVs can also help to regulate the abnormalities in TME, such as neovascularization, tumor metabolisms, and the stiff extracellular matrix barrier brought by tumor stromal cells. In short, oncolytic therapy offers various advantages.

To date, scientists have made a number of preclinical attempts and clinical trials of both naturally occurring OVs (e.g., reovirus and vesicular stomatitis virus)^6–8^ and genetically engineered OVs (e.g., adenovirus, vaccinia virus and herpesvirus),^9–11^ with some encouraging data. From H101 for nasopharyngeal carcinoma admitted by China in 2005 to Delytact for malignant glioma approved in Japan in 2021,^12,13^ a total of four functionally compensatory OV products have been approved for clinical treatment. OV has gradually managed to secure its place as a powerful anticancer agent in cancer treatment options. As most investigators have found that OVs are ideally suitable for combination strategies compared to single modality therapies because of the complexity of mechanisms involved in the progress of OV to take action in the complex environment of tumors. The development of combination methods implementing antitumor drugs yields synergistic or additional antitumor benefits, for which clinical validations through well-designed and statistically sound clinical trials are required.^14,15^

This review provides a comprehensive overview of oncolytic virotherapy, especially addressing the basic principles of how OVs take effect in the context of complex TME. Furthermore, recent advances in genetic engineering strategies to construct versatile OVs will be discussed in full range. The accurately selected combination options for cancer treatment and the outcomes of ongoing associated clinical trials are especially worthwhile keeping an eye on because the valuable information would provide future directions for the development of more advanced OVs with maximized capabilities.

## The dominate types of OVs

Regarding the antitumor mechanisms, although OVs share properties, different types or subtypes of viruses are being scrutinized for efficacies to cope with various pathological conditions. OVs are derived from single- or double-stranded DNA or RNA viruses according to nucleic acid type. ssRNA and dsDNA viruses are the most prevalent in OVs products, except for reovirus (dsRNA) and parvovirus (ssDNA). dsDNA viruses mainly include adenovirus, vaccinia virus, herpesvirus, etc., while ssRNA viruses are composed of two main categories: positive-sense (coxsackievirus, Seneca Valley virus, poliovirus) and negative-sense (measles virus, Newcastle Disease virus, vesicular stomatitis virus). The genetic information of positive-sense ssRNA viruses is directly translated into protein by ribosomes of host cells, while the nucleic acid of negative-sense ssRNA viruses is complementary to the viral mRNA, which must be transcribed into positive-sense RNA before it can be translated into protein. OVs can also be divided into naturally attenuated viral strains and genetically modified viral vectors according to their structures (Table 1).

**Table 1:** Features of selected oncolytic viruses

	Herpesvirus	Adenovirus	Vaccinia virus	Reovirus	Coxsackievirus	Seneca Valley virus	Poliovirus	Measles virus	Newcastle disease virus	Vesicular stomatitis virus
Model										
Genome	dsDNA 150 kb	dsDNA 36 kb	dsDNA 190 kb	dsRNA123 kb	ss(+)RNA 28 kb	ss(+)RNA 7 kb	ss(+)RNA 7.5 kb	ss(-)RNA 16 kb	ss(-)RNA 15 kb	ss(-)RNA 11k b
Capsid symmetry	Icosahedral	Icosahedral	Complex	Icosahedral	Icosahedral	Icosahedral	Icosahedral	Icosahedral	Helical	Helical
Virion	Enveloped	Naked	Complex coats	Naked	Naked	Naked	Naked	Enveloped	Enveloped	Enveloped
Replication site	Nucleus and cytoplasm	Nucleus and cytoplasm	Cytoplasm	Cytoplasm	Cytoplasm	Cytoplasm	Cytoplasm	Cytoplasm	Cytoplasm	Cytoplasm
Methods of entry	HVEM, nectin-1, nectin 2	CAR, CD46	Receptor-mediated endocytosis	JAM-A	CAR/ICAM1/DAF	Endocytosis	CD155	SLAM, CD46	Sialic acid	LDLR
Blood–brain barrier penetration	–	–	–	+	–	+	+	–	+	–
Advantages	Large genome to insert large fragments and multiple transgenes; drug to shut-off	Feasibility of manufacturing high viral titers; ease of genome manipulation; inherently potent lytic activity	Fast, efficient spreading virus; high-speed life cycle; up to 40kd large gene fragment insertion; enough knowledge due to smallpox	Good adaptability for intravenous injection; displaying no dose-limiting toxicity	Good adaptability for intravenous injection	Nonpathogenic in human	Clinical trial experience	Clinical trial experience	Nonpathogenic in human	High-speed life cycle; nonpathogenic in human
Disadvantages	Pathogenicity; ubiquitous nAbs	Extensive tissue tropism	Pathogenicity	Rarely gene-editing	Pathogenicity; ubiquitous nAbs	Clinical trials were not entirely satisfactory	Highly pathogenic in neurons of the human	Pathogenicity	Rarely gene-editing	Clinical trials were not entirely satisfactory; rarely gene-editing

*dsDNA* double-stranded DNA, *dsRNA* double-stranded RNA, *ssRNA* single-stranded RNA, *HVEM* herpesvirus entry mediator, *CAR* coxsackie adenovirus receptor, *JAM-A* junctional adhesion molecule A, *ICAM1* intercellular adhesion molecule 1, *DAF* decay-accelerating factor, *SLAM* signaling lymphocytic activation molecule, *LDLR* low-density lipoprotein receptor, *nAbs* neutralizing antibodies

### Herpesvirus

Herpes simplex virus (HSV), an enveloped virus with dsDNA protected by the nucleocapsid, and surrounded by the tegument, has two specific serotypes (HSV-1 and HSV-2).^16^ HSV contains a large genome of at least 150 kb and a complex structure, which provides the possibility for the insertion of relatively large fragments and multiple transgenes.^17^ Four major viral glycoproteins, gB, gD, gH and gL, are expressed on the surface of the HSV envelope enabling the binding with various cellular receptors.^18^ During infection, the envelope fuses with lipid bilayers of the cell membrane to expose the nucleocapsid to the nuclear membrane.^19^ The viral genome is then released into the cytosol, and transported into the nucleus where transcription initiates. The viral gene transcription and protein synthesis are strictly regulated by the herpesvirus genome. According to the order of transcription and translation, viral proteins are divided into immediate-early proteins, early proteins and late proteins,^20^ in which modifying the genes encoded by these proteins is a common method. As a cytolytic virus, HSV can infect multiple types of cancer cells and quickly replicate, spreading the progeny viruses easily within neoplasms.^21^ In addition, anti-HSV drugs like Acyclovir can be utilized to ensure the safety of oncolytic HSV (oHSV) to counteract virulence.^22^ Even though more than half of the population possesses neutralizing antibodies against HSV, it can still evade the host immunity through different mechanisms, rendering it a model for an ideal OV vector.^23^ Currently, HSV-1 is one of the most commonly used strains of OVs. The representative works include T-VEC,^24^ G207^25^ and G47Δ.^26^ The strain HSV-2 is also drawing increasing attention and is under investigation at the moment. An oHSV-2 named OH2 has launched phase I/II clinical trials in solid tumors recently, but its modification strategy is the same as that of T-VEC.^27^

### Adenovirus

Adenovirus is a 90–100 nm naked virus composed of approximately 26–45 kb dsDNA genome wrapped by an icosahedral capsid that is comprised of hexon trimers and penton bases (PB).^28^ The N-terminal of fiber knobs is attached to PB, and C-terminal is responsible for identifying cellular receptors, which is a desired place to be modified for selective targeting.^29^ Among a total of 57 different serotypes, Ad2 and Ad5 belong to subgroup C, being the most wildly used as oncolytic adenovirus (oAd).^30^ Most oAds infect cells by combining coxsackievirus and adenovirus receptor (CAR) except subtype B and some of subtype D that exploit CD46 for infection.^31^ Upon virus internalization through receptor-mediated endocytosis,^32^ the viral particles are disassembled and exposed capsids that enter the cytoplasm by lysis of endosomal membrane and are subsequently transported along microtubules to the nuclear envelope, where viral genomes import into the host nucleus.^33^ E1A and E1B are key early genes that activate the replication and transcription of subsequent viral genes of Ad2 and Ad5.^34^ The conserved region (CR) 2 of E1A proteins replaces retinoblastoma (Rb) proteins of the E2F transcription factor in infected cells and initiates the cell cycle of the quiescent cell to enter S-phase.^35^ The E1B-19 kDa protein and E1B-55 kDa protein encoded by the *E1B* gene prevent post-infection cell death, prolonging the viral replication. Specifically, the E1B-55 kDa protein binds to p53 and induces its degradation, and the E1B-19 kDa protein acts as an antiapoptotic factor.^36,37^ Adenoviruses are one of the most widely studied viruses because they provide several advantages, such as the feasibility of manufacturing high viral titers, ease of genome manipulation, and inherently potent lytic activity.^38^ However, adenovirus has an extensive tissue tropism, which addresses the significance of enhancing selective replication in tumor cells of oAds to ensure biosafety. For example, E1A and E1B gene deletion is a common method to generate replication-defective adenoviral vectors.^39^ Following the success of H101, the first oncolytic agent approved for clinical use in the history of oncolytic virotherapy,^40^ Onyx-015,^41^ CG0070,^42^ etc., has also achieved inspiring results consecutively in clinical trials.

### Vaccinia virus

Vaccinia virus (VV) is a dsDNA virus approximately 190 kb, belonging to the orthopoxvirus genus. The virus particle is about 270 × 350 nm in size and appears as brick shaped structure.^43^ Unlike other dsDNA viruses, intracellular mature virions (IMV), the main particle type of VV, has an asymmetric and complex structure that consists of a nucleoprotein core enclosed by a single lipoprotein membrane.^31,44^ VV enters host cells either by fusion with the host cell membrane at a neutral pH environment or through receptor-mediated endocytosis under acidic pH.^45^ The process is assisted by the entry-fusion protein complex consisting of eight viral proteins: A16, A21, A28, G3, G9, H2, J5 and L5,^46^ but no host cellular receptors have been clearly identified. VV contains enzymes required for initiation of viral post-infection transcription located in the viral core,^47^ and its replication and progeny assembly occur exclusively in endoplasmic reticulum (ER) surrounded cytoplasmic mini-nuclei.^48^ Its selective targeting is highly dependent on thymidine kinase (TK) gene, encoding the essential enzyme for viral replication. TK is usually overexpressed in malignant cells but rarely expressed in normal cells. In this way, scientists generated TK-knockout VV strain that only replicates in cancer cells.^49^ In addition, VV secretes viral proteins to activate EGFR-RAS pathway of host cells to further promote the synthesis of TK.^49^ Some prominent advantages of VV include fast and efficient spreading of the virus due to high-speed and active life cycle, as well as up to ~40 kd gene insertion capacity and well-studied genome due to the acknowledgment of smallpox.^43^ The most famous oncolytic VV, JX-594, in particular, shows potential for intravenous injection by resisting the effects of antibodies and complement.^50^

### Reovirus

Reovirus is a naturally occurring non-enveloped dsRNA virus that structurally consists of an outer capsid and an inner core.^51^ Reovirus enters the host cell primarily through receptor-mediated endocytosis by engaging with junctional adhesion molecule A (JAM-A),^52^ which served as a receptor for reovirus. Reovirus can be utilized as an OV to target cancer cells since JAM-A is overexpressed in a series of cancers, including breast cancer,^53^ non-small cell lung cancer,^54^ diffuse large B-cell lymphoma,^55^ and multiple myeloma.^56^ Upon infection, the outer capsid is acid-dependently cleaved in endosomes and the transcriptionally active core is subsequently released.^57^ The transcription and translation event for the assembly of progeny virus happen in viral inclusions located in the cytoplasm. Throughout the whole life cycle of virion production, maturation and egression, the virus does not enter the host nucleus.^58^ Another mechanism of reovirus to selectively target tumor cells is via the prevalent mutation of RAS signaling in tumors.^59^ The modulating RAS in cancer cells is related to PKR inactivation.^60^ In normal cells, PKR can bind to dsRNA of reovirus and arouse its autophosphorylation and activation, further phosphorylating eIR2 to be inactive, which prevents the translation of viral transcripts.^61^ Three serotypes have been identified; among them, the type 3 Dearing strain (T3D) has been adopted to manufacture OVs called Reolysin^®^.^62^ It shows considerably good adaptability for intravenous injection and potent antitumor effects, exhibiting no dose-limiting toxicity or irritation.^63^

### Other OVs

In addition to the four common types of OVs discussed above, other viruses have also shown efficacy in OV treatment, especially ssRNA viruses that are classified into ss(+)RNA and ss(-)RNA viruses. For ss(+)RNA, they are usually in a smaller size that come from *Picornaviridae* family as the name suggests, including coxsackievirus, Seneca Valley Virus (SVV) and poliovirus. These viruses are naked, representing icosahedral capsid in electron microscopy (EM) appearance. They replicated in the cytoplasm to avoid the insertion of foreign genes.^64^ Mechanically, coxsackievirus binds to the surface molecules, such as DAF and ICAM‑1 for cell entry, which is overexpressed in multiple cancers, like melanoma, multiple myeloma, and breast cancer cells.^65^ Although coxsackieviruses are facing the challenge of being neutralized by antibodies, different serotypes seem not likely to cross-react. In the case of SVV, mainly SVV-001 strain is nonpathogenic in humans and has been shown to infect neuroendocrine tumor.^66^ However, previous clinical trial results were not entirely satisfactory.^67^ Poliovirus is highly pathogenic in human anterior horn motor neurons; therefore, its toxicity must be attenuated. Gromeier et al. replaced the viral internal ribosome entry site (IRES) with an IRES of the related human rhinovirus type 2 (HRV2) to target glioblastoma multiforme (GBM), since the receptor of poliovirus CD155 is overexpressed on glioma cells.^68^

The ss(-)RNA OVs are unique in some aspects. Measles virus and Newcastle disease virus (NDV), which belong to the *Paramyxoviridae* family, have a relatively large viral particle size but a relatively short length of RNA. Measles virus utilizes the signaling lymphocytic activation molecule (SLAM) receptor or CD46 as the receptor for cell entry,^69^ while NDV infects via sialic acid on host cells.^70^ Upon cell entry, the two viruses exercise their life cycle in the cytoplasm, and propagate infection via cell‑to‑cell fusion, resulting in the formation of multicellular aggregates and cell death.^64^ However, the measles virus may cause measles through respiratory transmission, and attenuated strains (e.g., Edmonston strain) are recommended for use.^71^ For NDV, both attenuated and non-attenuated strains would be adopted for OV construction, because it is an avian virus that poses no harm to humans, and MEDI5395 has been studied for oncolytic activity.^72^ Another OV worth of being discussed would be vesicular stomatitis virus (VSV). VSV glycoproteins (G protein) attach and fuse with host cells via the non-specific expressed low-density lipoprotein (LDL) receptor. Following receptor-mediated endocytosis, internalization occurs within the endosomes at low pH condition.^73^ Although the infectious receptors for VSV do not appear specifically at the cancer cell surface, selective targeting is achieved due to the defects of the antiviral interferon (IFN) signaling pathway in those cells. Four VSV OVs including VSV-IFNβ-NIS have been evaluated in the clinical trials; however, most of them are in phase I at present.^74^

## The arsenal for OVs: modification strategies

### “Boot Camp”: training wild-type viruses into “qualified soldiers”

#### Improving the tumor-targeting selectivity of OVs

Training wild-type viruses into tumor-specific OVs is the prerequisite step that can be described as training civilians into recruits, which may happen either in the process of infection or replication. The training process needs to be carried out according to the characteristics of the viruses and tumor cells. Different types of viruses show different natural affinity and preferential replication tendencies in different tumor cells, while genetically engineered OVs are designed for enhanced targeting selectivity. There are two main modification strategies for improving the fidelity of OVs in tumor targeting. The first is to increase the affinity and the binding activity of the viruses to the overexpressed receptors at the tumor surface. Alternatively, the target accuracy could be enhanced by utilizing the characteristics (e.g., the abnormalities in the pathways/protein expressions in tumor cells) of the tumor cells to differentially improve the viral replication efficiency^75^ (Table 2).

**Table 2:** Improving the targeting selectivity of OVs

Modification types	Name	Viral type	Specific methods	Features of targeted tumor cells	Ref.
Natural tumor tropism	Poliovirus	Poliovirus	–	Via CD155	^72^
	Reolysin^®^	Reovirus	–	With activated RAS signaling	^77^
	M1	Alphavirus	–	Lack of ZAP	^97^
Viral-specific entry receptors	Ad5/F35	Ad	Chimeric Ad consisting of the knob and shaft of Ad35 combined with Ad5	Via CD46	^80^
	AdCMVLacZ 425-S11	Ad	A neutralizing anti-adenovirus fiber single-chain Fv (scFv) Ab (S11) fused to an scFv Ab directed against the epidermal growth factor receptor	Via EGFR	^81^
	HSV-1 P-V528LH	HSV-1	Modified with P-V528LH adapter fused to an EGFR-specific monoclonal antibody consisting of gD ectodomain binding region of nectin-1	Via EGFR and nectin-1	^82^
	R-LM113/R-115	HSV-1	Inserted an scFv HER2 into the gD of oHSV	Via HER2	^83^
	MV-PNP H^blind^antiCD20	MV	Fused to an scFv CD20	Via CD20	^84^
Dysregulations of genes or signaling pathways in tumor cells	VV WR strain	VV	B18R blocks the α subunit of the IFN receptor, inhibiting antiviral responses of the cells	With the α subunit of IFN receptor blocking	^85^
	H101	Ad	E1B 55KD mutation	With p53 mutation	^87^
	CG0070	Ad	The human E2F-1 promoter was engineered before the E1A gene	With Rb mutation	^90,91^
	T-VEC	HSV-1	γ34.5 gene deletion	Widely	^86,92–94^
	OA-4MREs	Ad	MREs of miR-124, miR-128, miR-146b and miR-218 controlling E1A gene	miRNAs should be downregulated by at least 50%	^106^
Overexpression genes or proteins in tumor cells	JX-594	VV	Inserting the human GM-CSF gene into the thymidine kinase (TK) gene loci	With TK overexpression	^98^
	GD55	Ad	Endogenous E1A promoter of E1B 55kD-deleted Ad was replaced by GOLPH2	With GP73 overexpression (HCC cells)	^100^
	CRAd-S.RGD CRAd-S.F5/3 CRAd-S.pk7	Ad	Ads with survivin promoter	With survivin promoter overexpression	^101^
Transcriptional/translational dually regulated (TTDR) OVs	AU27	HSV-1	ICP27 is regulated by prostate TSP ARR2PB and 5’UTRs of rFGF-2	With eIF4E overexpression	^108^

*OVs* oncolytic viruses, *Ad* adenovirus, *ZAP* zinc-finger antiviral protein, *HSV-1* herpes simplex virus 1, *VV* vaccinia virus, *IFN* interferon, *MREs* miRNA response elements, *GM-CSF* granulocyte-macrophage colony-stimulating factor, *TK* thymidine kinase, *HCC* hepatocellular carcinoma, *TSP* tumor-specific promoters

#### Improving the OV infection via tumor cellular receptors

First of all, the characteristics of the affinity of naturally occurring viruses have been perceived by using certain tumor-specific cellular proteins. Due to altered pathways within the tumor cells, these receptors have been upregulated. CD155 is widely overexpressed on the surface of many tumor cells, promoting tumor cell invasion and migration. It happens to be the natural receptor of poliovirus, rendering poliovirus the ability to selectively infect tumor cells.^76^ Reolysin^®^, a wild-type variant of reovirus (i.e., T3D strain), has been demonstrated to have oncolytic activity across a spectrum of malignancies depending on RAS signaling.^77^ HSV gD protein binds to herpesvirus entry mediator (HVEM), which has been reported upregulated in melanoma, gastric cancer and hepatocellular carcinoma (HCC).^78^

Since some receptors are still expressed in normal cells in a relatively lower amount, OVs are designed to recognize tumor-upregulated receptors, allowing the virus for an enhanced fidelity. For a typical example, subgroup C adenovirus (Ad), a commonly used OV, infects host cells by the combination of the fiber knob of Ad and coxsackie adenovirus receptor. However, the efficacy of targeting virotherapy remains limited for the differential expression levels of coxsackie adenovirus receptors on different tumor cells.^79^ To circumvent the deficiency, there are some strategies to transform Ad capsid for viral retargeting. The first way is to switch fiber knob serotype by reconstructing the chimeric fibers with knob domains derived from another serotype Ad. Based on the differences in receptor utilization, for example, Yang et al. summarized Ad5/F35 (chimeric Ad consisting of the knob and shaft of Ad35 combined with Ad5) enhancing targeting and oncolytic effects on multiple cancers via CD46, which is highly expressed in most tumor cells.^80^ Another strategy takes heterologous retargeting ligands that are bispecific in binding to the fiber knob domain and a tumor-associated antigen (TAA). Haisma et al. fused neutralizing anti-adenovirus fiber scFv Ab (S11) to EGF-mediated adenovirus retargeting to EGF receptor-positive cells.^81^ A similar strategy to identify potential bi-soluble adapters for targeting cognate tumor receptors has been adopted in HSV-1 modified with P-V528LH adapter fused to an EGFR-specific monoclonal antibody consisting of gD ectodomain binding region of nectin-1, which is found overexpressed in breast and colorectal cancer.^82^ OV can also be modified to accurately target human epidermal growth factor receptor 2 (HER2). Leoni et al. inserted an scFv HER2 into the gD of oHSV to target the primary HER2-Lewis lung carcinoma-1 (HER2-LLC1) tumor.^83^ CD20 is overexpressed in several hematological malignancies, such as CD20-positive non-Hodgkin’s lymphoma (NHL). A CD20-targeting measles virus (MV)-based vector was constructed to target lymphoma and showed promising tropism.^84^ The growing emergence of tumor-specific receptors or antigens would provide OVs with more attractive modification approaches for advanced targeting accuracy. To be noted, the sequences for the scFvs to be carried have to be evaluated carefully for optimal binding ability.

#### Enhancing the replication efficiency of OVs in tumor cytoplasm

Improving the replication capability of OVs is an effective approach to developing tumor targeting. Some viruses have their own mechanisms to promote replication. The B18R protein produced by some orthopoxviruses blocks the α subunit of the IFN receptor, inhibiting antiviral responses of the cells, promoting virus replication.^85^ In the case of Talimogene laherparepvec (T-VEC), a modified oncolytic herpes simplex virus 1 (oHSV-1), has been approved by the Food and Drug Administration (FDA) as the first oncolytic virotherapy for the treatment of melanoma. The mutation in α47 gene gives rise to an early expression of the US11 gene, which was reported to induce viral replication in tumor cells.^86^

Molecular engineering of viruses also makes it possible to modify viruses to allow their replication to be more efficient specifically in cancer cells. It has been proposed that both loss of the tumor-suppressor genes and dysregulations of signaling pathways in tumor cells would aid in viral replicative selectivity. In Ads, the gene encoding E1B 55kD, which may inactivate the tumor-suppressor p53 by ubiquitination and keep the virus alive in cells, was deleted in many oncolytic Ads such as H101 and ONYX-015.^87^ E1B 55kD-ablated adenoviruses are more sensitive to p53-induced apoptosis in normal cells versus malignancies where p53 is often mutant that allows high-efficiency viral replication in tumor cells.^40,88^ However, another study argued that the tumor-specific replication of ONYX-015 was later shown to be due to the loss of E1B-mediated late viral RNA export from nucleus to cytoplasm, rather than p53-inactivation.^89^ Nevertheless, a similar idea was applied to another oncolytic adenovirus, CG0070, which used the human E2F-1 promoter to drive the viral E1A gene.^90^ The retinoblastoma tumor-suppressor protein (Rb), commonly mutated in bladder cancer, contributes to transcriptionally active E2F-1 that enables the high-level expression of E1A for CG0070.^91^ T-VEC deficient in neurovirulence factor (γ34.5) leads to tumor-selective replication.^92–94^ The biofunction of γ34.5 is to block the shut-off of protein synthesis and interferon responses in host cells during virus infection.^95^ The γ34.5(-) HSV-1 is, therefore, more sensitive to the above antiviral responses in normal cells. Since tumor cells are often deficient in such host response mechanism, the γ34.5-deficient virus such as T-VEC can selectively replicate in cancer cells.^96^ Alphavirus M1, which belongs to the togavirus family, was isolated from culicine mosquitoes collected from Hainan, China. The expression of ZAP is high in normal cells, which is the mechanism of resisting virus-induced cell death.^97^ Lin et al. previously reported that M1 virus selectively killed tumor cells lacking zinc-finger antiviral protein (ZAP).

On the other hand, it is noteworthy to mention that some overexpressed genes or proteins yielded from tumor cells may happen to further support the biophysiological activities of OVs. For example, JX-594, a transgene-armed and targeted OV developed with vaccinia virus (VV), was modified by inserting the human granulocyte-macrophage colony-stimulating factor (GM-CSF) gene into the TK gene loci, thus destroying the inherent ability of the virus to transcribe TK.^98^ TK is required for the replication of JX-594; therefore, replication occurs only in cells that highly express TK, such as most tumor cells.^99^ Tumor-specific promoters (TSPs) convey high tumor-specific transcriptional activity in an array of cancer types, and thus may serve as genetic engineering sites of OVs for transcriptional targeting. GP73 is a better biomarker for HCC diagnosis than AFP. Taking advantage of this feature, adenovirus GD55, in which endogenous E1A promoter of E1B 55kD-deleted Ad was replaced by GOLPH2 (a Golgi membrane glycoprotein GP73) promoter, has been demonstrated to have more accurate targeting in HCC.^100^ In cholangiocarcinoma (CCA), Ads with survivin promoter were designed by Zhu et al., exhibiting higher activity. The survivin promoter shows greatly low expression levels in normal cells and indicates strong tumor specificity.^101^

Nevertheless, it raises the question whether the productivity of virions is the demanding factor influencing the outcomes of oncolytic therapy. According to the review by Davola and Mossman, infected cell protein 0 (ICP0)-defective oHSV-1 and oHSV-2 viruses showed a negative correlation between in vitro replication and in vivo antitumor activity.^102,103^ In another study, non-replicative VV Ankara (iMVA) was more effective not only in suppressing melanoma tumors but also in the growth of distant tumors than replicating MVA.^104^ The strong oncolytic efficiency has also been revealed in some oHSV-1 lacking neurovirulence with a much-impaired replication capability.^2^

Addressing for the issue, some researchers still insist that viral replication is equally important, and have invented a non-attenuated viral skeleton equipped with transcriptional or translational elements that control the regulation of viral essential genes. Knock-in of TSPs in OVs, such as ZD55, GD55, exhibit advanced replication efficiency in tumor cells for their transcriptional characteristics.^100^ Besides transcriptional regulatory elements, translational switches also provide means to control the replication. The miRNA response elements (MREs) that are able to combine with the corresponding miRNA can be engineered into 3’ UTR region of the essential gene expression of OVs.^105^ The matching standards of MREs and miRNAs are supposed to obey the following criteria: (1) miRNAs should be downregulated by at least 50% in malignant tissues compared with noncancerous tissues. (2) MREs should bind to miRNAs as little as possible in tumors but as much in normal cells, so that more targeted replication can be carried out. Yao et al. built an oncolytic Ad with MREs controlling E1A gene named OA-4MREs, including MREs of miR-124, miR-128, miR-146b and miR-218, which resulted in increased viral replication and oncolysis in primary glioma cells compared to ONYX-015.^106^ Similarly, the GC-rich 5’-UTR of genes is often associated with malignancies and metastasis in cancers (e.g., rFGF-2). These regions give rise to a wide range of secondary hairpin structures, thereby inhibiting the translation of downstream mRNA in normal cells. However, such hairpin structures can be untwisted when there is an overexpression of elF4E in cells. Coincidentally, eIF4E is often overexpressed in tumor cells. Therefore, if this type of 5’-UTR is constructed in OV backbone, it can make it difficult for OVs to replicate in normal cells.^107^ Based on the above discussion, transcriptional/translational dually regulated (TTDR) OVs that combine the advantages of transcriptional and translational elements could be ideal for keeping the balance between replication and oncolysis ability. For example, ICP27, an essential viral gene of oHSV-1, could be regulated by prostate TSP ARR_2_PB and 5’UTRs of rFGF-2 that enhanced both OV replication and tumor specificity.^108^ All in all, TTDR-OVs are a promising strategy in OV construction.

#### Enhance the security of OVs

The viruses have been long perceived as pathogenic microorganisms that may induce pathogenicity. For this consideration, the safety of OVs has been doubted and has attracted much attention from researchers, especially natural OVs, which have been proven to kill tumor cells, were thought to destroy normal cells at the same time, like chemotherapies. For this concern, many studies have demonstrated to transform wild viruses into attenuated OVs with improved targeting fidelity, although there are still concerns related to viral recombination, toxicity, cytotoxic products, and off-target possibilities.^109^ Therefore, appropriate modifications of OVs for safety improvement are urgent for their clinical applications.

As mentioned above, the improvement of the selectivity and fidelity can enhance the security accordingly. There is no denying that deleting the γ34.5 gene is indispensable in oHSV-1 because this OV would not infect normal neurons; however, the mutation of TK is optional. For example, VG161 is an oHSV-1-based OV-containing TK gene developed by Virogin Biotech Canada Ltd.^110^ Although keeping the native TK gene partially compromised targeting accuracy of OVs, it helps to keep the sensitivity of VG161 to acyclovir or ganciclovir. In this case, the virulence of VG161 and safety could be effectively controlled in clinical applications.^109^

Moreover, the off-target effect is another serious concern that leads to organ damage, especially for Ads, which have been reported to be enriched in liver^111^ and limit the adenoviral transduction in vivo. Based on this, Alba et al. found that Ad5-hexon binding to coagulation factor X (FX) mediated liver transduction. They developed genetically FX-binding-ablated Ad5-hexon vectors to alleviate the symptom.^112^ The retargeting strategy can also permit CAR-independent infection to prevent liver sequestration as described above.^113^

Some OVs have shown to present high safety in both animal experiments and clinical trials, such as NDV,^114^ VSV^115^ and SVV.^116^ None of them are human-contagious viruses and are not pathogenic to people. Meanwhile, they have potential natural targeting ability to some tumor cells and are receiving increasing attention in the field of OVs research works. However, the risks generated from environmental shedding and mutation or recombination of oncolytic agents with wildtypes should be noticed and assessed. For example, NDV strains pose a potential risk for animal infections, since birds are more vulnerable to engineered viruses.^113^ Likewise, the neuroticism-eliminated VSV strains tend to revert into virulent wild-type VSV upon passaging.^117^ These security issues exist not only in these seemingly secured OVs, but also in almost every other OV. In general, strain screening, enhancement of targeting capability and accuracy, decreased off-target toxic, mutation and recombination probability are inevitable methods for increased safety.

### “Cold and ancient weapons” of OVs: an oncolytic spear that pierces the target cells

When the “qualified soldiers” acquire the abilities of precision guidance, selective replication and reliable security, these OV soldiers are called upon take their weapons, that is, to be made express transgenes for further fighting against tumor cells. For the past decades, the antitumor mechanisms of OVs were mainly focused on directly infected cell oncolysis. Type I interferon and other antiviral signaling pathway are widely downregulated in cancers, making cancer cells more vulnerable to OVs that yield offspring through cell lysis.^8^ During cell lysis, the susceptibility of the cancer cell to the different forms of cell death depends on the types of viral vector and the corresponding transforming elements, which strongly influence the replication and efficacy of viruses.^64^ In this regard, an increased number of studies have considered the association between the factors influencing cell death and classifications of cell death, including apoptosis, necrosis, pyroptosis and autophagy, during OV development. However, as more is learned about OVs, we realize that this is a necessary but primitive aspect of OV construction, and thus the associated gene to be armed onto OV is termed as the “cold weapon” (Table 3).

**Table 3:** Different types of tumor cell death induced by OVs

Types of cell death	OVs name	Viral types	Modification methods	Tumor types	Ref.
Apoptosis	NDV La Sota strain	NDV	–	NSCLC	^124^
	NV1066	HSV-1	–	STAD	^125^
	H5CmTERT-Ad/TRAIL	Ad	H5CmTERT-Ad expressing secretable TRAIL	GBM	^126^
	FusOn-H3	HSV-2	FusOn-H3 armed with apoptosis activators Her2-COL-sFasL	BRCA	^127^
	Parvovirus H-1 (H-1PV)	PV	–	Glioma	^368^
	Semliki Forest virus (SFV)	SFV	–	OS and NSCLC	^369^
	CVB3	CV	–	LUAD	^370^
Necrosis/necroptosis	VV Lister-dTK	VV	Deleted TK	OC	^135^
	NDV Herts/33 strain	NDV	–	CCA	^136^
	WR/TK-/MLKL	VV	MLKL were inserted into VV vector	PCA	^137^
	M1	Alphavirus	–	TNBC	^138^
	MV-eGFP	MV	–	*h*Melanoma	^371^
Pyroptosis	ΔPK	HSV-2	Deleted ICP10PK	Melanoma	^148^
	VSV	VSV	–	*h*Melanoma and NSCLC and BRCA	^149^
	Ad-vp3	Ad	Armed with apoptin	CRC	^150^
	CVB3	CV	–	Colon cancer	^151^
	oHSV-1 RH2 strain	HSV-1	With γ34.5 gene-deficient	SCC	^152^
	NDV	NDV	–	PCA, such as GBM	^153^
Autophagy	OVV-BECN1	VV	oVV that expresses Beclin-1	Leukemia and myeloma	^156^
	OVV-Beclin-1	VV	oVV that expresses Beclin-1	Lymphoma	^157^
	oHSV-1 RH2 strain	HSV-1	With γ34.5 gene-deficient	SCC	^158^
	SVV-001	SVV	–	MB	^159^
	OBP-702	Ad	p53-expressing oAd	HOS	^163^
	Ad5/3Δ24hCG	Ad	Ad5/3 fiber-modified human chorionic gonadotropin (hCG)-expressing	HRPCa and LUAD	^372^
	NDV/FMW	NDV	–	LUNG	^373^
	HVJ-E	Sendai virus	–	*h*PRAD	^374^
	MV-Edm	MV	–	NSCLC	^375^

*OVs* oncolytic viruses, *NDV* Newcastle disease virus, *HSV-1* herpes simplex virus 1, *Ad* adenovirus, *PV* poliovirus, *SFV* Semliki Forest virus, *CV* coxsackievirus, *VV* vaccinia virus, *MV* measles virus, *VSV* vesicular stomatitis virus, *SVV* Seneca Valley virus, *TRAIL* TNF-related apoptosis-inducing ligand, *TK* thymidine kinase, *MLKL* mixed-lineage kinase-like protein, *oVV* oncolytic vaccinia virus, *NSCLC* non-small cell lung cancer, *STAD* stomach adenocarcinoma, *GBM* glioblastoma, *BRCA* breast cancer, *OS* osteosarcoma, *LUAD* lung adenocarcinoma, *OC* ovarian cancer, *CCA* cervical cancer, *PCA* pancreatic carcinoma, *TNBC* triple-negative breast cancer, *hMelanoma* human melanoma, *CRC* colorectal cancer, *SCC* squamous cell carcinoma, *MB* medulloblastomas, *HOS* human osteosarcoma, *HRPCa* hormone refractory prostate cancer, *LUNG* lung cancer, *hPRAD* human prostate adenocarcinoma

#### Apoptosis

Viral infection modulates cell death via death receptor-mediated pathways, where the death receptors, including Fas, TRAIL-R and TNF-R, form a death-inducing signaling complex (DISC) that mediates apoptosis.^118^ Viral infection regulates the binding of death receptors to their ligands (e.g., virally encoded proteins), which subsequently triggers caspase cascade and initiates extrinsic apoptosis,^119^ where tBID is cleaved from BID by Caspase-8 activation and mitochondria-mediated intrinsic apoptosis pathway is activated.^120^ The regulation of virus on death receptor-mediated apoptosis mainly stems from the overexpression of death receptors or their ligand on the cellular membrane of the infected hosts and the sensitivity increase of this apoptosis signaling.^121^ Death receptor-mediated apoptosis represents an efficient mechanism for virus-induced cell death and progeny dissemination.^122^ However, an interesting phenomenon may emerge where apoptosis is rapidly arrested at the onset of oncolysis, and as progression increases, apoptosis is enhanced, and tumor cells continue to divide. In the initial stage, different viruses can manipulate specific abnormal signaling factors within tumor cells to inhibit apoptosis, providing sufficient time and space for viral replication and reproduction. If cancer cells are highly susceptible to apoptosis, the number and the dose of the OV will be limited in the tumor.^123^ Mansour et al. observed that NDV La Sota strain could stably infect and there is a 2-log increase replicate in targeted cells with an overexpression of the antiapoptotic protein such as Bcl-xL, allowing the OV to propagate and form syncytia required for virus transmission.^124^ A study by Stanziale et al. also supported the finding that more NV1066 (an engineered oHSV-1) was found to be produced in OCUM-1 cells when exposed to an inhibitor of apoptosis named N-acetylcysteine (NAC) than in untreated cells, and the tumor lysis was also raised correspondingly.^125^ However, a series of studies confirmed the multiple roles of apoptosis in OV-induced cell death. An H5CmTERT-Ad expressing secretable trimeric tumor necrosis factor-related apoptosis-inducing ligand (H5CmTERT-Ad/TRAIL) was generated by Oh et al. and exhibited a more potent tumor-killing effect in contrast to a cognate control Ad by inducing strong apoptosis.^126^ Loya et al. armed FusOn-H3 (i.e., engineered oHSV-2) with apoptosis activators Her2-COL-sFasL to increase the caspase activation (especially caspase-3 and -8) in infected cells and bystander killing effect.^127^ NDV is one of the OVs that has been studied comprehensively in the mechanism of apoptosis. NDV-mediated induction of apoptosis includes the activation of endoplasmic reticulum (ER) stress,^128^ intrinsic and extrinsic apoptotic pathway.^129^ All in all, the switch-like modification of apoptosis is a noteworthy direction of OV transformation, which could work along OV replication and lysis in the future.

#### Necrosis/necroptosis

Necrosis is an irreversible and uncontrolled cell death manifested by rupture of the plasma membrane, swelling of organelles, leakage of intracellular contents and finally cell death.^130^ Necrosis occurs due to overwhelming deleterious stress from multiple responses, and it is almost always associated with an inflammatory response due to the release of ATP, heat-shock proteins, DNA, uric acid, and nucleoproteins, which lead to cascading inflammasome activation.^131^ For OV-induced cell death, other forms of death are usually prioritized, and uncontrolled necrosis is more likely to be an endpoint of the post-lytic signal transduction cascade. Modification strategies would not focus on necrosis, but might target downstream substances such as inflammasome release.

However, a form of programmed cell death with a morphology similar to necrosis has been found, termed necroptosis.^132^ As for a caspase-independent cell death, it requires the activation of the kinases RIPK1 and RIPK3 to assemble into necrosome. The necrosome then phosphorylates and activates mixed-lineage kinase-like protein (MLKL) for trimerization, leading to rapid membrane permeabilization and danger-associated molecular patterns (DAMPs) release.^131,133,134^ Although necroptosis is a common form of OV-induced cell death, there are few reports of modifications to enhance the effect. Oncolytic VV Lister-dTK was shown to induce necroptosis in ovarian cancer cells.^135^ The NDV Herts/33 strain triggered necroptosis in vitro.^136^ MLKL was inserted into VV vectors to induce necroptosis, conferring potent immunity to neoepitopes and antitumor properties.^137^ Transcriptomic analysis showed that M1 viruses activate necroptosis in triple-negative breast cancer (TNBC), but they amplified this effect not by modification but by binding to doxorubicin.^138^ A similar strategy was adopted by oHSV-1 + Mitomycin-C, which induced necroptosis to sensitize tumors to ICIs in an osteosarcoma model.^139^ Viruses also evolved necroptotic inhibited proteins to suppress pathogenesis during infection.^140,141^ During VV infection, the E3 protein of VV prevents the accumulation of Z-shaped RNA by competing with the N-terminal Zα domain, thereby inhibiting the recruitment of RIPK3 by ZBP1 and reducing necroptosis.^142^ As for the proper immunogenic death method, more attention should be paid to the modified OV with improved necroptosis.

#### Pyroptosis

For pyroptosis, OV can be directed to trigger and regulate pyroptosis in cancer cells, leading to tumor shrinkage or remission and eliciting a strong immune response.^143,144^ Modulation of the inflammatory pyroptotic cell death pathway has been shown to successfully inhibit the proliferation and metastasis of multiple cancer cell types and may become a prospective cancer treatment strategy.^143^ Faria et al. observed that activation of inflammatory vesicles consisting of NLR or ALR and a bipartite protein called ASC (apoptosis-associated speck-like protein containing caspase activation and recruitment domains), bind to caspase-1 and directly activate the caspase cascade,^145^ leading to pyroptosis by lysing the gas phase cortex. This then leads to the formation of pores in the cell membrane and membrane rupture, cell rupture, and death.^143,146^ In addition, they found that pyroptosis releases proinflammatory cytokines, such as IL-1β and IL-18, as well as various DAMPs, which initiate adjuvant antitumor immune responses. Furthermore, it cleaves Gasdermin D (GSDMD) to its active N-terminal fragment, which forms pores in the plasma membrane, leading to a form of inflammatory cell death known as pyroptosis.^145–147^ OVs also induce pyroptosis; for example, an HSV-2 mutant lacking ICP10PK (ΔPK) upregulates the secretion of inflammatory cytokines TNF-α, GM-CSF, and IL-1β through pyroptosis.^148^ Oncolytic VSV can trigger gasdermin E (GSDME)-mediated pyroptosis, leading to immune switching of the TME by recruiting cytotoxic T lymphocytes in the background and enhancing the efficacy of immune checkpoint therapy.^149^ The oAds-armed apoptotic protein, encoded by the VP3 gene of chicken anemia virus (CAV), induces pyroptosis by cleaving caspase-3 and GSDME, and significantly inhibits the growth of colorectal tumors.^150^ Other OVs, such as coxsackievirus B3,^151^ HSV-1,^152^ NDV,^153^ etc., have also adopted the death mode, and these pyroptosis-based anticancer drugs may open up new possibilities for OV therapy in the future.

#### Autophagy

Unlike apoptosis, autophagy level is continuously increasing in the whole process of oncolysis, and is the strongest during tumor cell lysis, which leads to autophagic cell death.^154,155^ Some experiments have tried to arm autophagy-related molecules on OVs to improve their effects, such as Beclin-1, the most commonly used protein in modification. Arming with Beclin-1 showed significant therapeutic efficacy of OVs through inducing autophagic cell death in hematological tumor-like leukemia and myeloma.^156,157^ Other strategies also indicated autophagy played a role in OV therapy. oHSV-1 RH2 strain with γ34.5 gene-deficient induced the formation of autophagosome and autophagic cell death in squamous cell carcinoma.^158^ SVV-001 could go through blood–brain barrier to eliminate intracerebellar xenografts from medulloblastoma by a subverted autophagy.^159^ Research aiming at Ads suggested a close relationship with autophagy. Several typical Ads proteins take part in the autophagy regulation, which is promoted by E1A and E1B but suppressed by E4.^155^ E1A links to the tumor-suppressor Rb to lose the E2F-1 from the Rb-E2F-1 complex. E2F-1 induces autophagy by upregulating autophagy-related proteins like ATG5 and LC3.^160,161^ On the other hand, E1B interacts with Beclin-1, resulting in the division of the Beclin-1-Bcl-2 complex and the induction of Beclin-1-dependent autophagy.^162^ Therefore, the transgenic Ads have aimed at these features. Besides the arming of Beclin-1, OBP-301 and its upgraded edition OBP-702 led to autophagic cell death through E2F-1 and downstream microRNAs (miRNAs).^163^ Nevertheless, autophagy is thought to be secular growth in OV therapy, but this result is more like a patchwork, lacking research into the whole process from the initial stage to the final cracking.

### “Hot and modern weapons” of OVs: drawing the magical immune gun

**Fig. 1:**
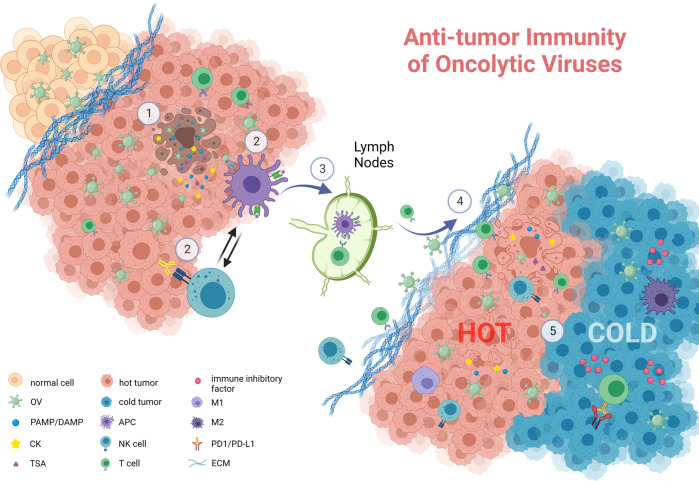
Stronger oncolytic immunogenicity of engineered OVs. ① When OVs cleave tumor cells, the viral progeny, TSAs, PAMPs, as well as DAMPs are released simultaneously, triggering ICD. ② Meanwhile, innate immunity is initiated, as DCs and NK cells collaborate for tumor clearance. ③ TSAs ingested by APCs soon migrate into lymph nodes, where T cells are activated, which infiltrate primary and metastatic foci to perform adaptive immunity. ④ In addition, engineered OVs are strengthened with the ability to break through ECM barriers, yielding inflammatory factors and chemokines, even reversing the immunosuppressive characteristic of TME. ⑤ In a collaborative effort, the engineered OVs may transform the immunologically “cold” tumor into “hot” tumor, also exerting an upgraded and more powerful antitumor immunity. Created with BioRender.com

With the in-depth research works on OVs regarding their underlying mechanisms, scientists have increasingly focused on OVs-mediated oncolytic immunogenicity. As soon as tumor cell lysis, the viral progeny is released along with TAAs, pathogen-associated molecular patterns (PAMPs) and DAMPs signals, accompanied by tumor ICD. PAMPs and DAMPs arouse innate immunity by binding toward the receptors such as the Toll-like receptor (TLRs). Furthermore, matured DCs and natural killer (NK) cells are stimulated, which are found to support OV-mediated tumor clearance.^164^ Specifically, TAAs and tumor neoantigens (TNAs) are caught by antigen-presenting cells (APCs) to set off adaptive immunity. Tumor-specific T cells prime from draining lymph nodes, CD4^+^ and CD8^+^ T cells are activated to exert tumor immune effect in the primary site. Meanwhile, OVs themselves or as platforms can stimulate the production of inflammatory factors (e.g., IL-2, IL-12, IL-15, TNF-α)^165^ and chemokines (e.g., CXCL9, CXCL10, CXCL11)^166,167^ in TME, where T-cell migration and infiltration is reinforced. Even though this is hinged by stromal barriers (e.g., extracellular matrix, ECM) in some tumors,^168^ OVs are expected to become a novel weapon to break through the structural barriers. Another difficulty encountered is that the infiltrated immune cells are challenged by immunosuppressive cells (e.g., tumor-associated macrophages; TAMs, myeloid-derived suppressor cells; MDSCs), inhibitory factors (e.g., IL-10, TGF-β) and upregulating immune checkpoints (ICs) on immune cells (e.g., PD-1, CTLA-4) in TME.^169^ Luckily, the counteract can significantly alter TME by inducing the immune response of proinflammatory T helper 1 (Th1) cell to combat immunosuppression have been proposed^170,171^ Even in some cases, the counteracts could deplete immunosuppressive cells, for example, to convert M2 macrophages into proinflammatory phenotypes.^172^ With such approaches, OVs turn the “cold” tumor into the inflamed, immunologically “hot” tumor,^1^ exerting the function of antitumor immunity. In general, the thought of updated OVs through the strengthening of tumor immunity is attractive, and the arming of related exogenous for these characteristics is worth of putting into efforts. The following sections will introduce the transformation of OVs in immunotherapy (Fig. 1).

#### ICD

Most forms of cancer cell death triggered by OVs belong to ICD, which has been regarded as a critical component in both OV development and tumor-specific immune responses in recent years.^173^ Specifically, cancer cells responding to oncolysis are allowed to mediate the signal of DNA damage responses, ER stress responses, autophagy, and necrotic plasma membrane permeabilization at the premortem stage, DAMPs such as surface-exposed calreticulin (ecto-CRT), heat-shock proteins (HSPs), extracellular ATP and high mobility group box 1 (HMGB1) are released, leading to the maturation of DCs and antigen presentation to T cells in TME.^174–176^ According to Guo et al., the right way of ICD is potent in elevating antitumor immune responses, thereby genetically engineered OVs can be armed with death-pathway modulating-associated genes to skew the infected cancer cells toward ICD as required.^177^ The majority of OV recombination for ICD promotion involves magnifying a particular form of cell death, some modifications to viral-specific genes may affect the occurrence of ICD.^177^ The OV *dl*922-947 is an adenoviral mutant with a 24 bp deletion in the E1A-Conserved region 2, which could induce ICD of malignant pleural mesothelioma (MPM) cells and trigger a cognate antitumor immune response.^178^ Generally speaking, ICD is like a key to open up oncolytic immunotherapy. However, there is still a lack of specific modifications for ICD recognition characteristics.

#### Promoting the function of antitumor immune cells

##### Proinflammatory cytokines

Cytokines are a large number of soluble proteins or glycoproteins with low molecular mass that regulate cell proliferation, cell differentiation and immune response by cell-to-cell communication. An increasing number of cytokines are perceived to play an active role in eliciting and reinforcing immune responses in tumors, thereby, various proinflammatory cytokines have been wildly studied as the accompanying transgene in OV modification. For example, GM-CSF, which is a hematopoietic growth factor that stimulates the proliferation of macrophages and granulocytes from bone marrow precursor cells,^179^ has been incorporated into the vectors of T-VEC,^180^ JX-594^98^ and other OVs. Such OVs carrying GM-CSF enhanced antigen presentation ability of DCs, thereby inducing the recruitment of NK cells and T cells and strengthening the immune responses.^98,180^ Interleukins are another type of cytokine that is initially thought to be restricted to leukocytes, but later is found to be produced by a wide variety of cells. IL-2, IL-12, IL-15, IL-23, etc., have been proven to have antitumor effects. Quixabeira et al. engineered an adenovirus coding for a human IL-2 variant (vIL-2) protein (Ad5/3-E2F-d24-vIL-2) aiming to uplift the antitumor response by enhancing the tumor-infiltrating lymphocyte (TIL) cytotoxicity in the context of immunosuppressive solid tumors,^181^ because vIL-2 can selectively activate CD8^+^ cytotoxic T cells and CD4^+^ helper T cells, not affecting Tregs.^182^ VG161 has been demonstrated to promote T cell and NK cell tumor infiltration by carrying IL-12 and IL-15.^110^ Oncolytic VV expressing IL-23 variants were generated by homologue recombination, resulting in activated T cells, and transforming the TME to be more conducive to antitumor immunity.^183^ TNF-α was also designed to be expressed by OV as an immune stimulant. Adenoviruses engineered to express tumor TNF-α and IL-2 were delivered in an anti-PD-1-resistant melanoma model, showing a prolonged survival time, an increased CD8^+^ T-cell infiltration and a reduced proportion of M2 macrophages and MDSCs.^184^ IFN-γ functions by promoting immune cell migration and propagation toward TME. The IFN-γ-encoding oncolytic VSV showed a better therapeutic effect in the lung cancer mouse model by droving more secretion of proinflammatory cytokines^185^ (Table 4).

**Table 4:** Transformations of OVs in immunotherapy

Modification aims	Name	Viral type	Specific methods	Ref.
Arming proinflammatory cytokines	T-VEC	HSV-1	GM-CSF	^180^
	JX-594	VV	GM-CSF	^98^
	Ad5/3-E2F-d24-vIL-2	Ad	IL-2	^181^
	HYPR-Ad-IL-4	Ad	IL-4	^376^
	VG161	HSV-1	IL-12 and IL-15	^110^
	RdB/IL-12/IL-18	Ad	IL-12 and IL-18	^377^
	vvDD-IL-23	VV	IL-23	^183^
	Ad armed with TNF-α and IL-2	Ad	TNF-α and IL-2	^184^
	VSV-IFNβ	VSV	IFN-β	^378^
	VSVΔ51-IFNγ	VSV	IFN-γ	^185^
Arming chemokines	VSV-CXCL9	VSV	CXCL9	^187^
	Adv-CXCL10	Ad	CXCL10	^188^
	vvDD-CXCL11	VV	CXCL11	^189^
	OV-Cmab-CCL5	HSV-1	scFv of cetuximab linked to CCL5	^190^
	NDV-MIP-3α	NDV	MIP-3α	^191^
	vv-CCL19	VV	CCL19	^379^
Expressing BiTE or TriTE	EphA2-TEA-VV	VV	Encoding a secretory BiTE which is targeted to EphA2 on lung cancer cells	^193^
	OAd-MUC16-BiTE	Ad	Ads armed MUC16-BiTE	^194^
	ICOVIR-15K-cBiTE	Ad	Engineered to express an EGFR-targeting BiTE (cBiTE) antibody under the control of the major late promoter	^195^
	MV-BiTEs	MV	MVs were generated to encode BiTEs targeting either human or murine CD3 and human CEA or CD20, respectively	^196^
	CAd-Trio	Ad	BiTE molecule specific for CD44 variant 6 incorporated into CAdDuo encoding IL-12 and PD-L1Ab to form CAd-Trio	^380^
The co-administration of OVs engineered to encode and secrete ICB	VG161	HSV-1	Encoded PD-L1 blockade that can block the upregulation of PD-L1	^110^
	CF-33-hNIS-antiPDL1	Poxvirus	Produce bioactive anti-PD-L1 antibody, which blocked PD-1/PD-L1 interaction	^204^
	ONCR-177	HSV-1	Armed both PD-1 and CTLA-4 antagonists	^205^
	LOAd703	Ad	Armed OX40L and 4-1BBL	^206^
	NDV-ICOSL	NDV	Expressing ICOSL	^208^
Arming immunosuppressive molecules inhibitors	AdLyp.sT	Ad	p32-binding LyP-1 peptide was genetically inserted into adenoviral fiber protein to inhibit TGF-β	^211^
	rAd.sT	Ad	Created a TERTp-regulated oncolytic Ads containing a soluble TGF-β receptor II‐Fc fusion (sTGFβRIIFC) gene	^212^
Aiming Tregs	VV-αCTLA-4	VV	VV-encoded αCTLA-4 were designed for CTLA-4+ Treg inhibition	^213^
	RdB/IL-12/DCN	Ad	Co-expressing IL-12 and decorin reduced Treg expression and overcomes Treg-mediated immunosuppression	^214^
Aiming TAMs	EnAd	Ad	TriTE-armed Ads to recognize M2, T cell and CD206, killing in M2 and a general increase in M1 marker expression	^215^

*HSV-1* herpes simplex virus 1, *VV* vaccinia virus, *Ad* adenovirus, *VSV* vesicular stomatitis virus, *NDV* Newcastle disease virus, *MV* measles virus, *GM-CSF* granulocyte-macrophage colony-stimulating factor, *IL-2* interleukin-2, *IL-4* interleukin-4, *IL-12* interleukin-12, *IL-15* interleukin-15, *IL-18* interleukin-18, *IL-23* interleukin-23, *TNF-α* tumor necrosis factor-α, *IFN-β* interferon-β, *IFN-γ* interferon-γ, *scFv* single-chain fragment variable, *CCL5* C-C motif chemokine ligand 5, *MIP-3α* macrophage inflammatory protein-3, *CCL19* C-C motif chemokine ligand 19, *BiTE* bispecific T cell engager, *CEA* carcinoembryonic antigen, *PD-L1Ab* programmed cell death 1 ligand 1 antibody, *PD-1* programmed cell death 1 ligand 1, *CTLA-4* cytotoxic T lymphocyte-associated antigen-4, *OX40L* OX40 ligand, *4-1BBL* 4-1BB ligand, *ICOSL* inducible co-stimulatory molecule ligand, *TGF-β* transforming growth factor-β

##### Chemokines

Chemokines are chemotactic cytokines that generate, recruit, and regulate the migration of immune cells. They coordinate the recruitment of immune cells to build a pro-tumorigenic microenvironment, and guide the cellular migration and interactions within TME for an effective antitumor immune response. Potent T-cell-attracting chemokines (i.e., CXCL9, CXCL10, CXCL11, CX3CL1, CCL2, and CCL5) play an important role in the activation of tumor immune contexture, and were considered to assemble into OVs.^186^ Eckert et al. engineered VSV to encode CXCL9 to mediate the recruitment of activated CD4^+^ and CD8^+^ T cells.^187^ In Adv-CXCL10, the chemokine CXCL10 is carried by an adenovirus, recruiting more CXCR3^+^ T cells into the TME to kill colorectal tumor cells via the CXCL10-CXCR3 signaling pathway.^188^ CXCL11-armed oncolytic poxvirus (vvDD-CXCL11) showed to enhance the infiltration of tumor-specific T cells and increase the number of local CD8^+^ cytotoxic T lymphocytes (CTLs) as well as granzyme B in TME of murine AB12 mesothelioma model.^189^ OV-Cmab-CCL5 is produced by expressing oHSV heterodimers consisting of a single-chain fragment variant (scFv) of cetuximab linked to CCL5. In GBM mice, OV-Cmab-CCL5 injections showed tumor shrinkage and prolonged survival due to enhanced migration and activation of NK cells, T cells, and macrophages.^190^ Other chemokines also contribute to oncolytic virotherapy; for example, Huang et al. constructed a recombinant NDV expressing macrophage inflammatory protein-3 alpha (MIP-3α) (NDV-MIP-3α) to elicit ICD and attract DCs in vitro and in vivo.^191^ OV-armed chemokine or cytokine strategies are effective in TME due to their short action distance and short half-life compared with cytokines alone, and also avoid the toxicity and risk of cytokine storm caused by high-dose systemic application of cytokines. However, cytokines may also induce stronger antiviral immunity, and whether they affect the subsequent effects of OVs remains to be explored (Table 4).

##### BiTE or TriTE

**Fig. 2:**
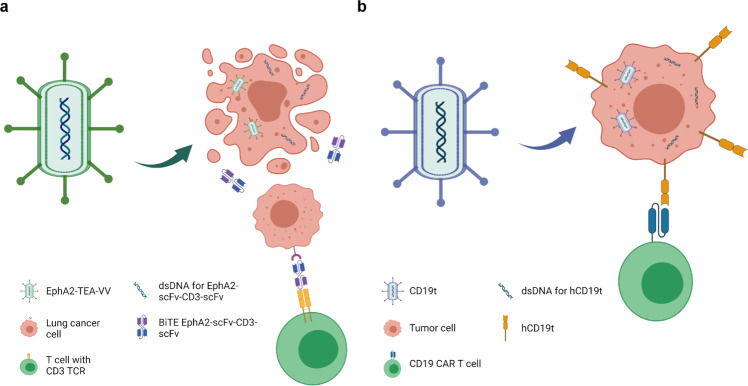
**a** Schematic diagram showing the mechanism of EphA2-TEA-VV EphA2-TEA-VV has been designed to express BiTE that targets EphA2 expressed on lung cancer cells and CD3 on T cells to stimulate T cells directly without antigen presentation by APCs. **b** CD19t for improving target identification and tumor control of CD19 CAR-T oVV was designed to express CD19 on the surface of infected tumor cells before oncolysis, which helps CD19 CAR T cells to probe and attack those CD19-marked tumor cells that could not be recognized and targeted essentially. Created with BioRender.com

Furthermore, the cutting-edge direction of OV immune-related genetic engineering is to combine bi- or tri-specific T cell engager (BiTE or TriTE) with OVs to directly stimulate T-cell immunity without antigen presentation by APCs. BiTE is a recombinant bispecific protein with two linked single-chain fragment variables (scFvs) produced by two individual antibodies, one targeting a TAA and the other targeting a cell-surface molecule (i.e., CD3) on T cells. On this basis, TriTE connects one more on T cell (i.e., CD3 and CD28).^192^ Like cytokines, these molecules remain drawbacks such as short biological half-life, rapid excretion, poor residence time in TME. Luckily, the problems could be solved when they become a team with OVs. A VV encoding a secretory BiTE, named EphA2-TEA-VV, has been designed to target against EphA2 in lung cancer cells (Fig. 2a). T cell activation, INF-γ and IL-2 secretion, as well as induced bystander killing of non-infected tumor cells were observed.^193^ Oncolytic Ads armed MUC16-BiTE targets highly glycosylated mucins that are overexpressed in ovarian cancers, leading to the improvement of MHC I antigen presentation, the proliferation and activation of T cells, the cytotoxicity against MUC16^+^ tumor cells, as well as remodulation of the TME.^194^ Other strategies such as ICOVIR-15K-cBiTE^195^ and MV-BiTEs^196^ have similar effects. The merging of two treatments complements each other, circumventing tumor heterogeneity, poor drug delivery and insufficient T cell infiltration. In a word, these modification strategies for antitumor immune cells are the mainstay, with the BiTE or TriTE technique being especially prospective in OV manipulation (Table 4).

#### Fighting against even converting antitumor immunosuppression

Even though antitumor immune cells could be found occasionally infiltrated into the tumor, they still encounter great challenges for the immunosuppressive characteristics of TME. Factors contributing to the immunosuppressive TME, such as low expression of antigen presentation molecules and neoantigens by tumor cells,^197^ the secretion of immunosuppressive cytokines,^198^ elevated expressions of ICs, as well as the recruitment and activation of immunosuppressive cells,^199^ are established via tumor autocrine or paracrine signaling network. Current immunotherapies have been developed to counteract such mechanisms, such as neoantigen vaccines, monoclonal antibody therapy and immune checkpoint blockade (ICB),^200^ which have been effective to some degree in hematologic cancers and some kinds of solid malignancies. However, tumors are always crafty opponents that adjust the cross-talks between immune and non-immune cells, as well as the ratio and constitution between effecter cells and tumor cells, thereby formatting a new TME that favors tumor growth, and inducing another round of tumor immune evasion and acquired drug resistance. In view of the main issues, OVs and the combination strategies have shown their potential in this field, and could be beneficial to overcome the resistance against immunotherapies to optimize the clinical outcomes of patients.^5^

**Fig. 3:**
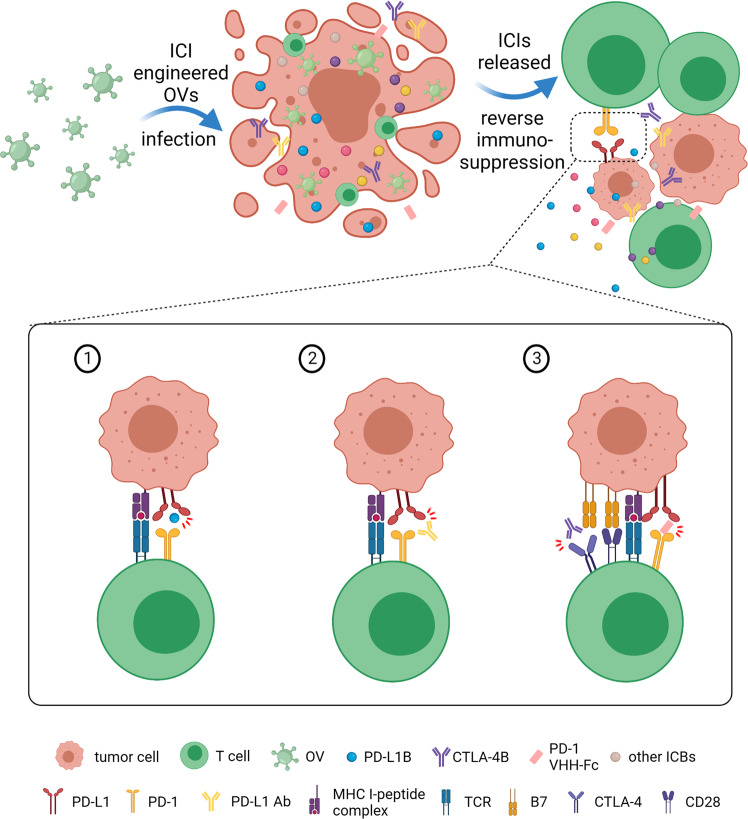
Three OV-engineered examples of ICIs expressing for reversing immunosuppression ICI-armed OVs infect tumor cells, subsequently releasing ICIs into TME to take effect. ① VG161 expresses PD-L1 blockade. ② CF-33-hNIS-antiPDL1 produces bioactive anti-PD-L1 antibody. ③ ONCR-177 secretes both anti-PD-1 VHH-Fc and anti-CTLA-4 mAbs. These strategies are used to block immune checkpoints and cooperate with OVs to enhance antitumor immunity. Created with BioRender.com

ICB therapy has been proven to be a remarkable strategy to restrict immunosuppressive signals and restore antitumor immune responses by targeting checkpoint receptors or ligand checkpoint molecules, such as PD-1/PD-L1 or CTLA-4, LAG-3 and TIGIT.^201^ In fact, limitations of ICB still exist in different tumors depending on the immunogenicity and components of TME.^202^ In another aspect, OV single treatment would cause upregulated expression of PD-L1.^203^ Therefore, the OVs engineered to encode and express ICB have provided a synergistic approach to overcome immunosuppression. VG161 has been manipulated to express PD-L1 blockade that refrains from interactions between PD-L1 and PD-1 expressed on T cells.^110^ CF-33-hNIS-antiPDL1 is another OV-producing bioactive anti-PD-L1 antibody, which blocked PD-1/PD-L1 interaction and was shown to reduce peritoneal tumor burden and improve the survival of xenograft mice.^204^ Interestingly, anti-PD-1 single variable heavy chain domain (VHH)-Fc and CTLA-4 monoclonal antibody armed ONCR-177 has been demonstrated potent antitumor activity in multiple immune-competent tumor models, which could be further improved by co-treatment with ICBs (Fig. 3).^205^ Meanwhile, OVs with other ICBs including OX40L (NCT02705196),^206^ VISTA^207^ and ICOS^208^ are under active investigation.

Solid tumors with immune-silent profiles are always accompanied by upregulated expressions of immunosuppressive molecules, causing immunotherapy failure. Preclinical studies have remarkably ameliorated the resistance by using TGF-β inhibitors, which provide inspiration for the modifications of OVs against tumor immunosuppression.^209^ In some studies, OVs are equipped with genes encoding TGF-β signaling pathway-related molecules to improve anti‐PD‐1 and anti‐CTLA‐4 responses,^210,211^ addressing the significance of ICB therapy in cancers of immune-desert phenotype. rAd.sT, a transforming TGF-β signaling-targeted oncolytic Ad, was combined with Meso-CAR T cells to treat breast cancer. The OV was found to reduce tumor burden at the initial stage, while CAR T played the role later during treatment.^212^ Tregs are immunosuppressive subsets of mainly CD4^+^ T cells that limit the proliferation and survival of T cells through different mechanisms. VV-encoded αCTLA-4 was engineered for CTLA-4^+^ Treg suppression, and a significant reduction in lung metastases was observed in the VV-αCEA TCE (αCEA BiTE-engineered VV) and VV-αCTLA-4 combination group.^213^ Oncolytic adenovirus co-expressing IL-12 and decorin reduced Treg expression and circumvent the Treg-mediated immunosuppression in the 4T1 orthotopic breast cancer model.^214^ TAMs, especially M2 in a narrow sense, are also one of the key cells to orchestrate the immunosuppression in TME. There was also a study that aimed to deplete M2-like macrophage subsets and developed TriTE-armed Ads to recognize M2, T cell and CD206. These were then cultured with DLD-1 tumor cells. Surviving macrophages are characterized by upregulated M1-associated markers and exhibited preferentially decreased M2 markers, suggesting TME repolarization toward a proinflammatory state. Further in vivo experiments of these agents are worthy of thorough exploration.^215^

Summarizing the above strategies on modified OVs that combat immunosuppression, the core idea is always to transform the “cold tumor” into the “hot tumor”. Plus, the combination of OV and ICB, especially PD-1/PD-L1 blockade, is one of the most frequently adopted approaches and most promising to enter clinical trials that may benefit more patients with “immune desert” tumors (Table 4).

As the main antitumor pathway of OVs, the genetic engineering strategy of OV based on antitumor immunity has been a research hotspot. Several aspects of OV immunotherapy, including ICD, immune stimulation, and immunosuppressive resistance, require multifaceted modifications. Based on the current understanding on TMEs and novel oncology drug development strategies such as bispecific antibodies, different types of transgene combinations are selected for accurate and flexible OV therapy.

#### Balance between the antiviral and the antitumor immunity

**Fig. 4:**
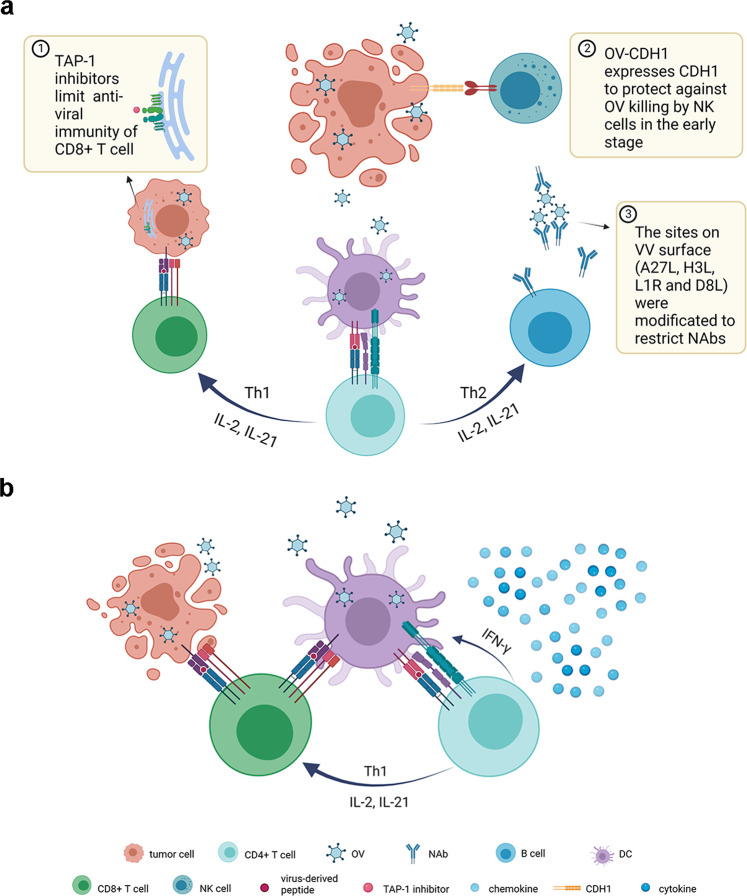
**a** OV-modification strategies against antiviral immune responses. ① Expression of TAP-1 inhibitor to limit antiviral CTLs for BV49.5. ② Expression of CDH1 to protect against NK cytotoxicity during the early stage of OV treatment. ③ Modification on enveloped membrane sites of VV (A27L, H3L, L1R and D8L) to restrict NAbs. **b** The possible mechanism of antitumor immunity benefiting from antiviral immune responses induced by OVs OV-induced antiviral immunological events may create an inflammatory TME. Besides that, IFN-γ produced by antiviral CD4^+^ T cells approves some DCs to cross-present specific epitopes to CTLs, resulting in ICD and more elicited TSAs that can reinforce antitumor immune responses. Created with BioRender.com

While antitumor immunity is a powerful weapon in OV therapy, the concomitant antiviral immune responses cannot be ignored. The existing theory holds that antiviral responses, including the clearance mediated by the early antiviral activity of NK cells, the viral antigens presentation to CD4^+^ helper T cells by mature DC, following the neutralizing antibody produced by B cells, and killing effect of CTLs, limit the infection and replication of OVs and then lead to the restriction of oncolytic effect.^216^ Researchers have attempted to inhibit antiviral immune responses by arming transgenes. Pourchet et al. created BV49.5 (an oHSV-1 with the bovine herpesvirus UL49.5 and US11 genes that replaced γ34.5 genes) to limit antiviral immune recognition of CD8^+^ T cells by inhibiting the transporter associated with antigen processing (TAP), which has shown significant efficacy in the bladder and breast cancer in murine models.^217^ OV-CDH1 was engineered to express CDH1, encoding E-cadherin, an inhibitory ligand for KLRG1 that expressed on NK cells, to protect against NK cytotoxicity during the early stage of OV treatment.^218^ For VV, there have been relatively mature modifications on antigenic epitopes on the viral surface to effectively restrict the cohesion with neutralizing antibodies (NAbs). The common sites that have been identified as major immunogenic proteins including A27L, H3L, L1R and D8L were imbedded into the viral enveloped membranes to reduce the immunogenicity elicited by the virus in vivo (Fig. 4a).^219^

However, some voices asserted that OV-induced antiviral immune responses hold antitumor benefits. If the rationale behind this could be appropriately understood and exploited, a stronger OV-based treatment can be developed.^216^ Following exposure to OVs, antiviral-related cytokines, chemokines, and signal molecules are activated and aggregated to induce proinflammatory antiviral responses, with a subsequent amplification of downstream cascades.^216,220^ These antiviral immunological events in TME may turn “cold” tumors into “hot” tumors, and even overturn tumor-associated immunosuppression to establish the basis for antitumor immunity in OVs.^216^ Besides, DCs exert phagocytic effects to integrate viral antigens.^221^ Although they present viral antigens to CD4^+^ T cells leading to the antiviral immune responses, IFN-γ produced by the antiviral CD4^+^ T cells approve some DCs to cross-present specific epitopes to CD8^+^ T cells, which in turn attack OV-infected tumor cells by lytic or non-lytic mechanisms,^222,223^ resulting in OV-induced ICD.^152^ As a result, it promotes another round of exposure to TSAs and the activation of CTLs in the process of OV transmission.^223^ Based on the above discussion, we should consider adjusting antiviral immunity in OV modification cautiously to maintain a delicate balance, thereby promoting and prolonging sustainable replication and infection while avoiding adverse effects on the desired anticancer immunity (Fig. 4b).

## “Auxiliary weapons” of OVs: anti-angiogenesis, reversing metabolic reprogramming and ECM barrier breakthrough

### Anti-angiogenesis

Sustained abnormal angiogenesis is one of the hallmarks characterized by most cancers, driven by the needs for nutrition transport and metabolic exchange.^224^ While TME resides in a hypoxic condition, “angiogenic switch” remains open and active, causing vessels to continuously sprout and expanding neoplastic growth by the abundance of pro-angiogenic factors such as vascular endothelial growth factor-A (VEGF-A).^225^ Such vasculature is typically aberrant and immature, characterized twisted and leaky, and accompanied by unstable endothelium, erratic blood flow, and insufficient pericyte coverage, causing a sustained hypoxia-regulated angiogenic vicious loop.^226^ Thus, this has become a hurdle for antitumor immune therapies, including OVs.^227^ The kinky neovasculature and pressure limit the delivery of antitumor agents (poor deposition of OVs in TME) and leukocytes including immune cell infiltration. The hypoxic and acidic TME also limits the replication and spread of OVs.^228^ These realities remind researchers to figure out novel approaches against anti-angiogenesis.

**Fig. 5:**
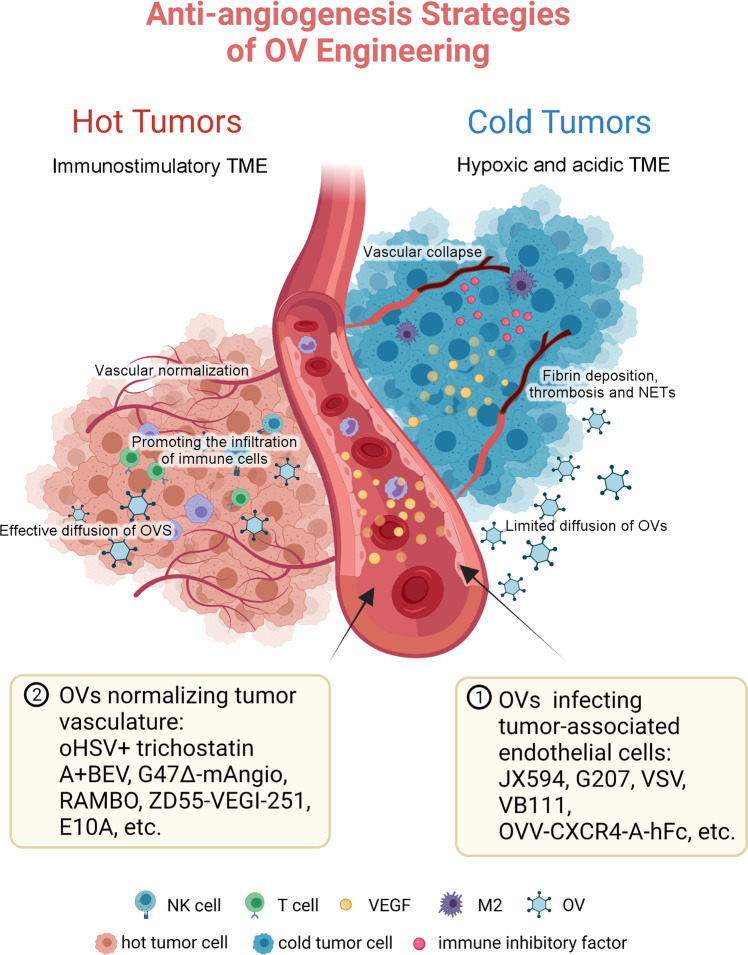
Different strategies of OV engineering for anti-angiogenesis and the possible induced phenotype of TME. ① Some OVs have been modified to attack tumor-associated endothelial cells, while the immunosuppressive TME provide a perfect niche for “cold” tumor development. ② Normalizing the tumor vasculature may promote immune cell infiltration and OV diffusion, giving rise to “hot” tumors. Created with BioRender.com

Conventional OV construction armed with anti-angiogenic weapons is dedicated to disseminate nascent vascularization. Some OVs have shown a preference for infecting tumor-associated endothelial cells themselves. For example, JX-594 can selectively infect endothelial cells in tumor-related vascular systems with increased VEGF and FGF-2 signals.^229^ G207, an oHSV-1, was found to replicate actively in CD31^+^ endothelial cells and reduced tumor neovasculature in malignant peripheral nerve sheath tumors (MPNSTs) model.^230^ Breitbach et al. adopted a three-dimensional (3D) reconstruction of infected tumors, revealing the direct infection and damage to the tumor vasculature by VSV.^231^ Other OVs were modified to target tumor vasculature or receptors of endothelial cells. VB111, an Ad5 containing a modified murine pre-proendothelin promotor (PPE-1-3X), Fas, and human TNF receptor 1, could infect tumor vessels and improve antitumor effects in the thyroid cancer xenograft model.^232^ In a triple-negative 4T1 breast carcinoma syngeneic mouse model, an oVV expressing CXCR4 antagonist was efficacious in destroying preformed tumor vasculature, inhibiting spontaneous metastasis and increasing overall tumor-free survival rate.^233^ However, classical anti-angiogenesis OVs may have adverse effects, including the reduction of blood flow inside the tumor, the aggregation of neutrophils in TME, and the accumulation of viruses on tumor rims.^234^ Although the outcome may be favored by the killed tumors’ starvation and apoptosis, some scientists doubted that the situation might be unfavorable for the infiltration and dissemination of OVs, which is undesired for the continued stage in oncolytic therapy. The loss of intratumoral blood flow was attributed to the recruitment of neutrophils and vascular collapse by OVs attacking endothelial cells, leading to the fibrin deposition and thrombosis, as well as neutrophil extracellular traps (NETs) that capture OVs and prevent them from spreading and delivering of subsequent drugs.^228^ On the other hand, elevated tumor hypoxia and acidic TME are disadvantaged to the survival and functions of some OVs, especially MVs and HSV that are extremely sensitive to pH changes.^235,236^ Nevertheless, there have been no long-term studies on OV targeting tumor vasculature so far, and the specific effects in the long run remain to be investigated (Fig. 5).

In consideration of the possible contradictions mentioned above, researchers never give up seeking for means to normalize tumor vasculature. Anti-angiogenic agents like vascular endothelial cell growth inhibitors have been proven the efficiency, and can reverse the immunosuppressive properties of hypoxia and acidic TME, aiding in increased CTLs infiltration and conversion of TAMs into antitumor M1 phenotype.^237^ However, there may be several limitations to the anti-angiogenic agents for single use. These agents are cytostatic, not cytotoxic, which means they cannot directly kill the tumor cells and be curative. During the later stage of treatment, acquired or inherent drug resistance possibly follows, accelerating in progression or recurrence. In view of the shortcomings, OVs have been used as engineering platforms or combination agents to combat anti-angiogenesis because of their multifaceted oncolytic properties and plasticity. oHSVs have been investigated to combine tumor vasculature targeting drugs such as trichostatin A^238^ and bevacizumab (BEV),^239^ and both showed a VEGF inhibition and antitumor enhancement. Likewise, OVs can be modified to express vasculostatin. G47Δ-mAngio improved tumor lysis, anti-angiogenesis, survive rate, and decreased VEGF expression and BEV-induced invasion markers during BEV combination treatment in mice-bearing human glioblastoma.^240^ RAMBO, another angiostatin-armed oHSV, has a similar effect to G47Δ-mAngio,^241^ and also in subcutaneously implanted sarcoma tumors.^242^ Besides oHSV, Ads have been studied and reformed. Furthermore, VEGI-251 was inserted into a ZD55 Ad and became ZD55-VEGI-251, which inhibited endothelial cell proliferation and increased mitochondria-mediated apoptosis.^243^ Ad-endo, also known as E10A, encoding secreted human endostatin, has been demonstrated to inhibit tumor growth through anti-angiogenesis, and the phase II clinical trial (NCT00634595) also shown improved outcomes of chemotherapy in advanced nasopharyngeal carcinoma.^244^ More radically, ICI or ACT treatments with concurrent use of anti-angiogenic OVs may facilitate CTL infiltration and activation via vasculature normalization and well-oxygenated TME (Fig. 5).^245,246^

Although the strategies targeting tumor vasculature are not the mainstay of OV modification, it can be a promising antitumor weapon if the tumors are greatly affected by abnormal neovascularization. Future research is supposed to be more meticulous and precise on the following perspectives, namely the timing of combination therapies, the dose of therapeutic agents, and the detection of blood perfusion. The core concept of modern anti-angiogenic therapy is to tame neovasculature, rather than terminating.

### Reversing metabolic reprogramming

Cancer metabolism has become an increasingly popular research issue in recent years. Biological activities are inseparable from metabolism, for which tumors are no exception. Metabolic alterations are closely associated with the occurrence and progression of neoplasms. Since nutrient uptake is directed by oncogenes, the deregulation of those genes causes an abnormal intake of glucose and amino acid, as well as the opportunistic and irregular modes of nutrient acquisition.^247^ In this regard, it initiates the reprogramming of intracellular metabolism in cancers, where Warburg effect, also known as aerobic glycolysis, was the first and classical to be discovered. Normally, in glycolysis, glucose is usually fermented into lactic acid under hypoxia, but in tumor cells, such fermentation can also happen under oxygen-sufficient conditions.^248^ Although this effect greatly sacrifices the efficiency of ATP production, it allows glycolysis and TCA cycle intermediate to participate in more biosynthetic physiobiological activities and yield more NADPH.^247^ Other forms of metabolic reprogramming, including oxidative phosphorylation (OXPHOS),^249^ glutamine metabolism,^250^ fatty acid synthesis,^251^ etc., would induce the alterations of metabolic-driven gene regulation, metabolic interactions with TME, finally resulting in cell behavioral and functional change.^247^ Interestingly, tumors with highly similar genetic backgrounds but different tissue origins have different metabolic patterns. On the contrary, tumors with different genetic backgrounds but similar TME have similar metabolic patterns.

Metabolisms of tumor cells or other components in TME play a vital role in OV-mediated antitumor effects. At first, OV replication makes use of the host cell metabolic pathway to acquire raw materials such as lipids, amino acids, and nucleotides.^252^ In some cancer cells, it has been found that glycolysis is upregulated during type I IFN production when infected by OVs.^253^ Thereby, inhibition of abnormally upregulated glycolysis may be a strategy to enhance the sensitivity and oncolysis for some OVs, such as Ads,^254^ NDV^255^ and reovirus.^256^ In pyruvate metabolism, the phosphorylation of pyruvate dehydrogenase (PDH) suppresses its activation and promotes the production of lactic acid, which is common in TME. The evidence suggested that VV^257^ and reovirus^256^ could inhibit PDH activation through upregulated PDH kinases (PDKs) when infecting cells, but these effects coincide with the initiation of antiviral pyruvate metabolism. Based on this, scientists employed dichloroacetate-induced inhibition of PDK to re-activate the activity of PDH and prolong the survival of OVs in tumors. Also, PDH catalyzes pyruvate oxidation to accelerate TCA cycle flux, which has been shown to be beneficial for OV replication and oncolysis.^252^ Thus, different OVs can better exercise their biological characteristics in tumors by taking advantage of their respective metabolic adaptation.

In addition, the metabolic state in TME partly determines the antitumor immune effects of OV, in which metabolic depletion of immune cells is the major barrier due to the limitation of essential nutrients and the accumulation of immunosuppressive metabolites like lactic acid.^165^ Tumor-infiltrating T-cell responses are significantly affected by glucose restriction and dysfunction of mitochondria.^258^ Lactic acid has been shown to polarize TAMs toward M2 phenotype, which made Tregs adapt to low glucose TME.^259^ There have been a few attempts to utilize OV as a metabolic regulatory platform. VV has been engineered to express adipokine leptin to reprogram tumor-infiltrating T cell metabolism through the persistence of mitochondrial function and a higher OXPHOS in activated CD8^+^ T cells, resulting in active immune responses and promotion of memory responses to secondary tumor challenge in melanoma-bearing mice.^260^ Recombinant OVs that express other metabolic modulating proteins, such as insulin or IGF-1, have also been patented and investigated for their role in promoting metabolic reprogramming and immune effects of T cells (WO2019148109). Therefore, it is suggested that a more comprehensive consideration of the effect of metabolic reprogramming on an antitumor immune response would be adopted when designing the optimal OVs.

### ECM barrier breakthrough

As the major constituent in the TME, ECM provides the growth niche for most solid tumors. It builds up a physical barrier and plays a key role in cancer initiation, progression, metastasis, and drug resistance. Among them, the immunosuppressive effects exerted by stroma are the main mechanism of tumor progression and treatment failure. The deposition formed by various ECM-secreted components (e.g., collagen and elastic fibers) and ECM remodeling negatively affects immune cell infiltrations.^261,262^ A classic example is the desmoplastic ECM of pancreatic ductal adenocarcinomas (PDACs), which is called “immune desert”. Traditional chemotherapeutic and molecular targeted therapy can only maintain a few months of median survival time for unresectable PDAC.^263^ Scientists have realized that the compositions of ECM could serve as promising targets for PDAC and other tumors with similar pathophysiological conditions, like HER2-positive breast cancer,^264^ high-grade gliomas,^265^ although no approved ECM-targeting therapeutic is available currently.

In this respect, OV can be a powerful weapon to break down the structural barrier between non-infiltrated immune cells and TME. OV is usually administered and autonomously transmitted intratumorally, which provides advantages for drug delivery and being independent of vein perfusion. The OVs carrying modifiers of ECM-related molecules cause significant changes in TME by producing a series of inflammatory mediators and cytotoxic proteases to facilitate ECM degradation.^266^ Tedcastle and his colleagues have cloned actin-resistant DNase (aDNAse I) and hyaluronidase (rhPH20) into conditionally replicating group B adenovirus that expresses ECM-degrading enzymes, which enhanced therapeutic efficacy against colorectal adenocarcinoma xenografts.^267^ In glioblastoma, hyaluronidase-expressing oncolytic Ad, ICOVIR17, combined with PD-1 blockade, successfully induced tumor-associated proinflammatory macrophages and T-cell cytotoxicity locally and systemically.^268^ For pancreatic cancer, neurotensin peptide (NT)-conjugated polyethylene glycol (PEG) has been armed with oncolytic Ad (oAd/DCN/LRP-PEG-NT), which has the capability of ECM-degrading efficacy by chemically cross-linking to the surface of ECM and disrupting Wnt signaling pathway. This chemical-engineered oAd has exerted reinforced oncolytic efficacy against neoplasms.^269^ The novel OV-modification strategies focusing on breaking down tough ECM barriers for more efficient drug delivery are worthy of more in-depth research works.

## Combination strategies with oncolytic virotherapy in preclinical research works

Single agent-based tumor immunotherapy strategies may lead to drug resistance due to the heterogeneity and complex genetic mutation burdens occurred in tumors, as well as the miscellaneous constitutions in TME; the efficacy of monotherapies including OVs usually fails to reach an optimal antitumor outcome on its own. Luckily, OVs are highly flexible agents that can directly bring the key factors influencing tumor immunity into the TME. These auxiliary agents are considered as potent partners in combination therapies. Indeed, most of the preclinical studies have seen better efficacies of OVs in combination approaches. The other therapies that work collaboratively with OVs, including immune checkpoint inhibitors (ICIs), adoptive cell transfer (ACT) therapies, cytotoxic chemotherapies, or targeted drugs, are summarized, respectively, in Table 5.

**Table 5:** Combination strategies with oncolytic virotherapy in preclinical research works

Combined therapeutic agents	OVs	Tumor types	Ref.
ICIs			
PD-1 monoclonal antibody	VG161	PDAC	^270^
Anti-PD-L1	CF-33	CC	^271^
PD-1 Blockade	GLV-1h68	STS	^381^
Anti-PD-1 antibody	WR.TK-HPGD+	RCC	^382^
CTLA-4 blockade	NDV	MEL	^383^
CTLA-4 antibody	G47Δ	ESCC	^274^
TIGIT blockade	OVH-aMPD-1	CC; HCC	^275^
TIM-3 antibody	vvDD	Lung cancer	^276^
LAG-3 blockade	VV-scfv-TIGIT	CC	^277^
Targeted drugs			
Ruxolitinib	VSV-IFNβ	NSCLC	^278^
	VSV-Δ51; HSV-1-dICP0	MEL	^279^
TPCA-1	VSV	Glioma	^280^
Bortezomib	HSV-1 (34.5ENVE)	OHNC; GBM	^281,287^
BKM120	G47Δ	PC	^282^
MEKi	T-VEC	MEL	^283^
A8301	HSV1617	RMS	^284^
IDO inhibitor	JD0G	GBM	^285^
Rituximab	Reovirus	CLL	^286^
3TSR	NDV	EOC	^289^
Axitinib	G47Δ-mIL12	GBM	^290^
Sorafenib	JX-594	HCC	^292^
Sunitinib	Reovirus	RCC	^293^
Bevacizumab	hrR3	STAD	^239^
	G47Δ-mAngio	Glioma	^240^
	RAMBO	Glioma	^241^
	HF10	BRCA	^294^
MS-275	VSV	MEL	^384^
Trametinib	HSV-1	Glioma	^385^
PLX4720	Reolysin®	MEL	^386^
Alisertib	MV	Lung cancer	^387^
Cetuximab	C-REV	CC	^388^
Olaparib	dl922-947	ATC	^389^
ACT therapies			
CAR-T and iNKT	rTTVΔTK-IL-21	Solid tumors	^297^
CD19 CAR-T	CD19t	Solid tumors	^298^
CAR-T and TCR-T	MYXV	EOC	^299^
Dual-specific CAR-T	VSVm-IFNβ or reovirus	MEL; GBM	^300^
HER2.CAR T cells	CAd-VECPDL1	PC	^390^
CD19 CAR-T	AdC68-TMC-tCD19	solid tumors	^391^
GD2.CAR-T	Ad5Δ24	NB	^392^
TILs	IL-2 armed oncolytic poxvirus	CC	^301^
CCR5-overexpressing NK cells	CCL5-modified oncolytic VACV	CC	^302^
EGFR CAR-NK	OV-IL15C	GBM	^303^
NK T cells	VSVΔM51; reovirus	EOC; BRCA	^393^
Chemotherapies			
Cisplatin	MYXV	EOC	^394^
Paclitaxel	Rhabdovirus Maraba-MG1	TNBC	^395^
Doxorubicin	CGTG-102	STS	^396^
Mitomycin-C	CV A21	BLCA	^397^
Gemcitabine	dl922-947	PDAC	^398^
Temozolomide	NDV	GBM	^399^
Cyclophosphamide	Ad-VT	BRCA	^400^
Irinotecan	VV	mCRC	^401^

*NDV* Newcastle disease virus, *G47Δ* oncolytic virus delytact, *HSV-1* herpes simplex virus 1, *VSV* vesicular stomatitis virus, *MV* measles virus, *MYXV* myxoma virus, *CD19t* truncated CD19, *VV* vaccinia virus, *PDAC* pancreatic ductal adenocarcinoma, *CC* colorectal cancer, *STS* soft-tissue sarcomas, *RCC* renal cell carcinoma, *MEL* melanoma, *STAD* stomach adenocarcinoma, *ESCC* esophageal squamous cell carcinoma, *HCC* hepatocellular carcinoma, *NSCLC* non-small cell lung cancer, *OHNC* head and neck squamous cell carcinoma, *GBM* glioblastoma, *PC* prostate cancer, *RMS* rhabdomyosarcoma, *CLL* chronic lymphocytic leukemia, *EOC* epithelial ovarian cancer, *BRCA* breast cancer, *ATC* anaplastic thyroid carcinoma, *NB* neuroblastoma, *TNBC* triple-negative breast cancer, *BLCA* bladder urothelial carcinoma, *mCRC* metastatic colorectal cancer

### Combined with ICIs

#### PD-1/PD-L1 inhibitors

As one of the most successful ICIs at present, PD-1/PD-L1 inhibitors have made a quantum leap in the treatment of a wide range of tumors. However, for those cancers that develop an immunosuppressive TME, such as PDAC, GBM, patients gain little benefit from the monotherapy. Combination therapies of OVs and PD-1/PD-L1 inhibitors may overcome this dilemma. Mechanically, following the PD-1/PD-L1 blockade, the T cell recruitment and immunity activation in TME would be ideally stimulated by the OVs, because the blockade helped to ameliorate the immunosuppression. In preclinical studies, the promise of this strategy has been adopted. Our previous results demonstrated that VG161, together with anti-PD-1 monoclonal antibody (mAb), provided better therapeutic performance in PDAC humanized mouse model, and that a significant growth of CD8^+^ T cells and NK cells were observed in the combination group.^270^ A combination of CF-33 and anti-PD-L1 therapy showed durable antigen-specific antitumor immunity and long-term survival against colon cancer in a syngeneic mouse model.^271^ Interestingly, Nguyen et al. addressed the significance of the timing of anti-PD-1 mAb to be administered in the combination treatment with OV.^272^ They have concluded and compared five major drug administration strategies: (i) Anti-PD-1 lead-in → OV; (ii) Concurrent administration of anti-PD-1 and OV; (iii) OV lead-in → anti-PD-1; (iv) Concurrent therapy lead-in → anti-PD-1; and (v) OV lead-in → concurrent therapy. The “OV lead-in → concurrent therapy approach” or the “OV lead-in → anti-PD-1” resulted in significantly improved outcomes compared to the other therapy approaches according to the data from preclinical and clinical trials, which is consistent with the rhythm of treatment-induced cancer-immunity cycle. The latter option may be adapted to cases with little chance of receiving repeated intratumoral injections.

#### CTLA-4, TIGIT, TIM-3 and LAG-3 inhibitors

In addition to PD-1, there are several other immune checkpoint molecules such as cytotoxic T lymphocyte antigen-4 (CTLA-4), T cell immune receptor with immunoglobulin and ITIM domains (TIGIT), T cell immunoglobulin protein and mucin domain-containing protein-3 (TIM-3) and lymphocyte activation gene 3 (LAG-3) that were also shown to be overexpressed on TILs and can cause the immunosuppression, as well as the exhaustion and depletion of activated CD8^+^ T cells.^273^ Intratumoral oHSV G47Δ working with a systemic CTLA-4 antibody-induced T cell recruitment and a broad gene pool associated with T cell activation, as well as restrained the production of Tregs, suggesting a healthy regulation of the TME.^274^ OVH-aMPD-1 synergizes with anti-TIGIT and showed reinforced immune responses in both MC38 and Hepa1-6 implanted subcutaneous tumor models.^275^ However, in refractory lung cancer, vvDD monotherapy with anti-PD-1 or anti-TIM-3 mAb showed no apparent therapeutic benefit, even though TIM-3 antibody helped to elevate the PD-1 expression on CD4^+^ and CD8^+^ T cells, while dual blocking combined with vvDD can improve the outcome.^276^ An engineered VV-scfv-TIGIT combined with LAG-3 blockade showed to uplift the complete response rate of CT26-bearing mice by exerting strong antitumor effects.^277^ It is worth mentioning that some strategies incorporating more than two ICIs often achieve better tumor therapeutic results. However, the side effects and biosafety issues in options of combination treatments require further careful and rigorous considerations.

### Combination with targeted drugs

The emergence of targeted drugs indicates that tumor therapy has entered an era of precision medicine. Broadly speaking, both OVs and some of the ICIs are regarded as targeted drugs. Various targeted drugs that have been combined with OVs in clinical use will be discussed in this case. According to different biofunction of the drugs, they are mainly classified as angiogenesis inhibitors that block the formation of new blood vessels (e.g., sorafenib, bevacizumab), monoclonal antibodies that have specific targets on cancer cells (e.g., trastuzumab, cetuximab), proteasome inhibitors (e.g., bortezomib), signal transduction inhibitors (e.g., imatinib), histone deacetylase inhibitors (HDACi, e.g., vorinostat, belinostat), DNA repair inhibitors (e.g., olaparib), etc. Alternatively, they can be divided into small molecular drugs or large molecular drugs based on their molecular weight. Small molecular drugs can enter cells and specifically block or compete for key molecules involved in the targeted signaling pathway to play a therapeutic role. Large molecular drugs usually target cell membrane proteins.

Usually, targeted drugs can be served as assistants of OVs, exerting their respective advantages and synergistically stimulating the antitumor efficacies. Some drugs can antagonize the antiviral immune pathway. Ruxolitinib, a specific JAK-1/2 inhibitor, enhances the replication and activity of VSV-IFNβ by antagonizing antiviral JAK/STAT signaling.^278^ A similar phenotype was shown in VSV-dM51-treated melanoma, where JAK-1/2 inhibition increased OV sensitivity.^279^ In VSV-treated glioma cell lines, blockade of IKK/NF-κB signaling by the NF-κB kinase (IKK) inhibitor TPCA-1 has been demonstrated to reduce type I IFN-mediated antiviral responses.^280^ Other drugs such as bortezomib,^281^ PI3K inhibitor BKM120,^282^ MEKi^283^ have been assessed for the capability to facilitate viral replication in virotherapies. Targeted agents facilitating OVs to activate the immune system or suppress immunosuppressive cytokines and cells have also been discovered. HSV1617 and TGF-β inhibitor A8301 were employed as combination therapy in immunocompetent models bearing murine rhabdomyosarcoma, resulting in the generation of an enhanced antitumor T cell response and significantly prolonged survival compared to the single agent administration.^284^ The inhibition of Indoleamine-2,3-dioxygenase (IDO), which is related to antiviral function and immune escape mechanism, was explored to improve the oncolytic ability of JD0G in glioblastoma cells.^285^ Rituximab combined with oncolytic reovirus enhanced NK cell-mediated antibody-dependent cellular cytotoxicity (ADCC) against chronic lymphocytic leukemia.^286^ Bortezomib united with oHSV strongly induced necroptotic cell death and NK activation.^287^

Normalization of tumor vasculature prior to the OV administration resulted in systemically enhanced immunotherapy. As a small bioactive recombinant peptide, 3TSR acquires anti-angiogenic properties by binding to the CD36 receptor on endothelial cells to inhibit the proliferation and migration of endothelial cells.^288^ In epithelial ovarian cancer, the idea had been tested with NDV, leading to tumor regression in preclinical models.^289^ The VEFGR tyrosine kinase inhibitor (TKI) axitinib combined with G47Δ-mIL12 was associated with a prominent reduction in vascularity, and increased infiltrated macrophage and tumor necrosis in the MGG123 GBM model.^290^ Anti-VEGF therapy can modulate the immune homeostasis of TME via regulating cytokine expression, such as IL-1β, IL-6, CXCL1 in tumors.^291^ Sorafenib combined with JX-594 was superior to single agents and showed objective tumor responses in three HCC patients.^292^ Combination of reovirus with sunitinib, another VEFGR inhibitor, in renal cell carcinoma demonstrated increased IFN-γ produced by tumor-specific CD8+ T and an establishment of protective immunity upon tumor rechallenge.^293^ Furthermore, the association with bevacizumab has been demonstrated to inhibit angiogenesis, and enhance the viral distribution and survival throughout the infected tissues from an assessed animal in various oncolytic virotherapies.^239–241,294^ However, the trend in combination with targeted drugs is that OVs are engineered to carry small molecule drugs or their derivatives to achieve antitumor effects. The different combination strategies can be adopted for preclinical or mechanistic exploration.

### Combination with ACT therapies

There are a considerable number of studies employing combination therapy with modified OVs and ACT.^295^ ACT is a process that involves transferring a desired amount of qualified and active antitumor lymphocytes that are cultured in vitro to the patients for tumor regression. The therapy includes chimeric antigen receptor (CAR) T/NK cell therapy, T cell receptor engineered T cells (TCR-T) therapy, TIL therapy, etc. Compared to other cancer immunotherapies, ex vivo amplification of active lymphocytes is easier to acquire and more effective in producing a therapeutic effect, because the ex vivo culture is less likely to be influenced by the immune inhibitory factors, and the in vivo initiation of the immune response is rapid.^296^ The strategy has shown encouraging outcomes in melanoma, lymphoma and certain leukemias; nevertheless, there are limitations in epithelial tumors due to difficulties in target identification, ACT cell infiltration, and tumor heterogeneity.^266^ Combining OVs with ACT may help overcome the obstacles in solid tumor treatment by reversing TME. Chen et al. incorporated IL-21 into VV Tian Tan strain to create rTTVΔTK-IL-21 and assessed the therapeutic efficacy of OV monotherapy, which works in combination with CAR-T and iNKT in humanized B-NDG mouse model, suggesting that the combination therapies outperformed the monotherapy.^297^ The two options of the combination strategy can compensate for each other according to their own characteristics.

Priceman’s team came up with an ingenious solution to this problem. They designed an oncolytic VV expressing a truncated CD19 (CD19t) to improve target recognition of CD19 CAR. Targeting the labeled tumor cells further induces local immunity. CAR T cell-mediated killing also resulted in the release of the virus from dying tumor cells, thereby inducing persistent infection of OV19t (Fig. 2b).^298^ In addition, adoptive cells can also serve as systemic vehicles to deliver OV to tumor sites. Zheng et al. used CAR T and TRP-1 T cells as a high-efficiency carrier to systemically deliver the myxoma virus (MYXV) to homologous antigen-expressing tumors (CAR/TCR-T10%MYXV), inducing specific tumor cell death, autophagy, and showing a potent form of bystander killing that eradicates antigen-negative tumor cells that contribute to tumor elimination and adaptive immunity with suppressed antigen escape.^299^ Bispecific CAR T cells were also loaded with VSVm-IFNβ or reovirus to treat B16/CT2AEGFRvIII tumor-bearing mice. Compared with unloaded CAR T cells, OV-infected CAR-T allowed further in vivo expansion and reactivation of T cells through homologous enhancement and prolonged survival of mice with subcutaneous melanoma and intracranial glioma tumors.^300^ Another novel idea was that IL-2-armed oncolytic poxvirus stimulates the accumulation of tumor-specific TILs in hypoimmunogenic tumor tissues. Meanwhile, such tumor-specific TILs are transferred into patients following the ex vivo expansion. These OV-induced TILs lead to colon tumor regression and longer survival in MC38-bearing mice.^301^

NK cells have some inherent advantages in immunotherapy compared to T cells. NK cells stem from a variety of sources. There is little worry about NK causing graft versus host disease (GvHD) because the recognition is independent of human leukocyte antigen (HLA) matching. The “off-the-shelf” characteristic of NK cells provides an opportunity for large-scale commercial production. However, adoptive NK or CAR-NK cell therapy with OVs is in its fledgling stage. The combination of CCR5-overexpressing NK cells with a CCL5-modified oncolytic VACV showed better efficacy than single agents in a colon cancer model, and greater infiltration of NK cells in the TME compared with the prototype virus.^302^ OV-expressing human IL-15/IL15Rα (OV-IL15C) and off-the-shelf EGFR-CAR-NK cells have elicited strong antitumor responses in an orthotopic GBM mouse model.^303^ Taken together, these studies suggest that once OVs are engineered to promote the migration, infiltration, and activation of ACT cells in solid tumors, they can be a powerful tool to break through the bottleneck of ACT therapy.

### Combination with chemotherapies

Chemotherapy remains the mainstay of first-line conventional cancer therapies. Therefore, a considerable number of combination research works have incorporated chemotherapy alone, as described in the comprehensively and systematically codified guidelines.^291,304^ Here, we will discuss the phenomenon of different curative effects resulting from the different orders of chemotherapy and OVs. We believe that effective and sufficient viral replication is the prerequisite for OV to exert full antitumor impact. Successful replication of OVs is dependent on viable tumor cells. The use of early chemotherapy makes it difficult for OVs to obtain an ideal living environment to complete the life cycle, and a large number of tumor cells are killed, resulting in unsatisfactory effects. On the contrary, there is also controversy regarding the administration order between the OV and the chemotherapy, because the antitumor immune cells activated by OV may be killed by chemotherapy drugs. On the other hand, chemotherapeutic drugs may act as antiviral agents and reduce viral replication in TME, largely compromising the efficacy of OV monotherapy or chemotherapy, leading to an impairment in the combination results. In our study, we explored the pharmacodynamics of VG161 combined with gemcitabine + nab-paclitaxel in a mouse model of pancreatic cancer, which produced the best effect in the VG161 post-chemotherapy group, while post-chemotherapy combined with OVs did not show any gain with OVs benefit compared with monotherapy.^270^ Likewise, treatment with ONYX-015 prior to cisplatin, or adding them concurrently, has been evaluated in earlier studies in the survival of HLaC xenograft tumor models over infection with ONYX-015 following drug treatment.^305^ However, the specialized mechanisms need to be explored by high-throughput methods such as single-cell RNA sequencing.

## Clinical trials of OVs

Currently, a total of four OV products have been approved for marketing: Rigvir (SND005), Oncorine (H101), Imlygic (Talimogene laherparepvec, T-VEC) and Delytact (teserpaturev/G47Δ). Rigvir is an unmodified enteric cytopathic human orphan virus type 7 (ECHO-7), approved for the treatment of melanoma^306–311^ in Latvia in 2004,^312^ making it the first approved oncolytic drug. Later in 2006, the adenovirus-H101 was approved in China,^13^ for squamous cell cancer of head and neck or esophagus.^313^ However, the treatment efficacy of these oncolytic drugs primarily stems from their intrinsic oncolysis characteristics rather than stimulating antitumor immunity. Therefore, the therapeutic effect of single-drug treatment is still limited, and the treatment strategy is more focused on combination therapies.

In 2015, the U.S. FDA approved T-VEC, an attenuated HSV-1 encoding GM-CSF for the local treatment of unresectable cutaneous, subcutaneous and nodal lesions in patients with recurrent melanoma after the initial surgery.^314^ It has been shown that GM-CSF may stimulate MDSCs, resulting in diminished innate and adaptive antitumor responses in numerous cancers.^234,315,316^ An insertion of a sole GM-CSF into the virus seems not an ideal strategy. Results from clinical trials indicated that the administration of T-VEC and ICIs present robust synergistic effects,^317,318^ suggesting the prospective potentials for the combination use of OVs and ICIs. The recently approved OV, Delytact (G47Δ), showed survival benefits in patients with residual or recurrent glioblastoma, with a good safety profile.^26,319^ Interestingly, there were no transgenes used as payloads in G47Δ. It thus raised the question whether the modification should be made within the viral vector or the viral genome to be made carry more exogenous regulatory genes would give rise to an optimal effect?

**Fig. 6:**
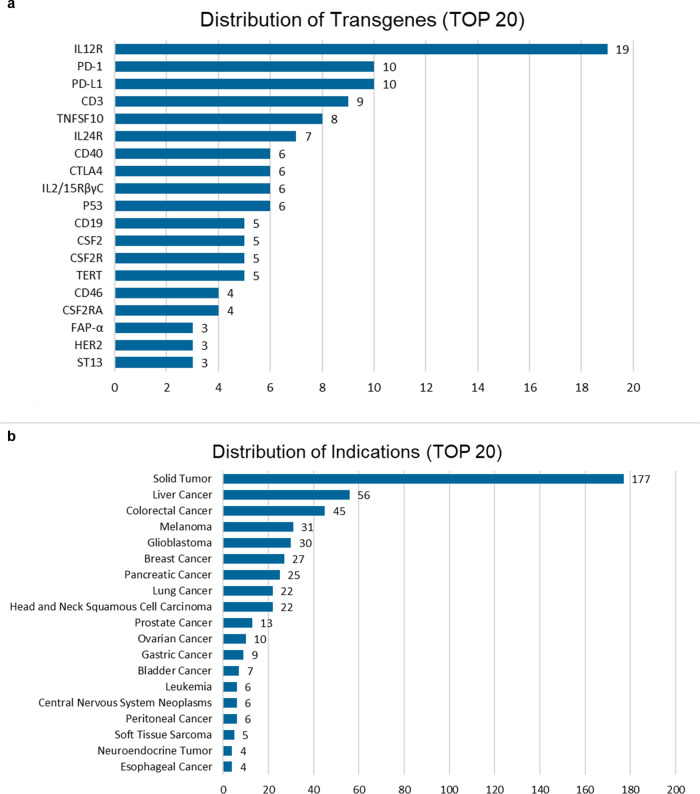
**a** The TOP 20 distribution of transgenes in clinical trials until 2022 **b** The TOP 20 distribution of indications in clinical trials until 2022

By 2022, there will be a total of 329 OV-related clinical trials registered in ClinicalTrials.gov. The majority of the clinical trials were phase I (*n* = 171; 52.0%) There were an additional 60 (18.2%) studies that were reported as phase I/II, 84 (25.5%) as phase II, 12 (3.6%) as phase III, and only 2 (0.6%) as phase II/III clinical trials. Details are listed in Supplementary Table 1. The TOP 20 distribution of transgenes and indications are listed in Fig. 6.

### Monotherapy of OVs

The significance of monotherapy is undoubtedly greater than that of combination therapy, but its success is also more difficult. At present, the performance of OV monotherapy has demonstrated good safety, but it has not yet shown amazing data in terms of efficacy. In 2015, Andtbacka et al. disclosed important data from OPTiM research,^314^ distinct performance of T-VEC in patients with advanced melanoma made it approved by FDA. In 2019, they reported the final results of the OPTiM research.^320^ A total of 436 patients with advanced melanoma were enrolled in the research and were arranged randomly into the group of the T-VEC treatment (*n* = 295) or the GM-CSF treatment (*n* = 141). As a result, the overall survival (OS) for the T-VEC group and GM-CSF group was 23.3 and 18.9 months; and durable response rate (DRR) was 19% and 1.4%; objective response rate (ORR) was 31.5% and 6.4%, respectively. Also, in the T-VEC group, 50 patients (16.9%) showed complete response (CR), while only one patient (0.7%) in the GM-CSF group achieved CR. Among patients with CR, 88.5% were estimated to survive at a 5-year landmark analysis. T-VEC also showed satisfying results in melanoma treatment in several other clinical trials.^321,322^ Andtbacka et al. reported Phase I clinical trial results of a coxsackie virus V937 in advanced melanoma,^323^ suggesting the DRR of 21.1%, the 12-month progression-free survival (PFS) and 12-month OS hit 32.9% and 75.4%, respectively. In addition, the phase I clinical data of PVSRIPO for patients with unresectable, treatment-refractory melanoma showed that the ORR of patients reached 33%.^324^ It can be seen that the overall performance of OV monotherapy in melanoma is fairly good, but it is largely because melanoma is highly immunogenic and more sensitive to immunotherapy.

Nevertheless, the efficacy of OV monotherapy in early HCC, which also represents better immunogenicity, is not as satisfactory as in melanoma. For example, JX-594 showed good safety in early clinical trials, and found that the tumor response and patient survival were related to the dose.^325^ However, the TRAVERSE study, which reported the results of the Phase IIb clinical trial of JX-594 in 2019, showed that there was no significant difference in median OS, overall response rate (RR), and time to progression (TTP) of the experimental group compared with the control group.^326^ To be noted, we have recently disclosed Phase I clinical data of a new OV VG161 in advanced liver cancer, showing that two patients with HCC in the first and second cohort had prolonged PFS of 3.7 and 11.5 months, respectively. In addition, all patients had received ICIs and had progressed before the enrollment, and significantly prolonged OS was seen in 5 patients who received ICIs after the trial (*P* = 0.025). It indicates that VG161 helped to re-construct the immunity in the TME of HCC, thus improving the sensitivity to ICIs.^327^ Furthermore, VG161 carries genes coding for IL-12, IL-15, and IL-15 receptor alpha subunit, along with a peptide fusion protein capable of disrupting PD-1/PD-L1 interactions.^110^ Compared to the JX-594 that only incorporates GM-CSF, VG161 stimulates a much more powerful antitumor immunity via various signaling pathways. This also implies that arming OVs with appropriate and adequate exogenous transgenes with mutual synergistic capabilities would be a desired option in future development directions.

Several clinical studies have reported the cases of OVs in malignant glioma treatment,^26,319,328–330^ among which the performance of G47Δ and G207 has drawn a lot attention. Todo et al. reported the phase II clinical results of G47Δ in residual or recurrent glioblastoma,^26^ showing that the 1-year survival rate was 84.2% and the median OS was 20.2 (16.8–23.6) months after G47Δ initiation. According to the report by Friedman et al., the median OS was 12.2 months in 12 patients with pediatric high-grade glioma treated with G207.^330^ Interestingly, both G47Δ and G207 are oHSV-1, and neither carries any transgene.

In addition to the above-mentioned clinical trials, OV monotherapy has been used in solid tumors,^331–335^ head and neck cancer,^336^ pancreatic cancer,^337–340^ epithelial cell carcinoma,^341^ bladder cancer,^342^ etc. They have shown encouraging outcomes in an array of cancer types. However, in some phase II clinical trials with larger samples, the therapeutic efficacy of OV monotherapy was relatively weak.^343–345^ In terms of safety, the most common treatment-related adverse events in OVs clinical trials are fever, chills, nausea, flu-like symptoms, fatigue and injection site pain. The overall safety is better than other immunotherapy products. In general, OV monotherapy has a certain therapeutic effect against cancers with better immunogenicity, but the overall performance is not as good as expected. Part of the reason is that the reported OVs are mainly products that do not carry or only carry a single transgene. These viruses have a limited ability to stimulate antitumor immunity, while the new generation of OV products that carry multiple immune-stimulating transgenes are expected to augment antitumor efficacy. Most of the new-generation OVs are being evaluated for the efficacy and safety in trials at present, and their ongoing clinical results are worth looking forward to.

### Combination therapy of OVs

In this field, the combination of OV and other drugs is one of the key development directions in the future. At present, the most common combination strategy with OV in clinical trials includes chemotherapy, immunotherapy, radiation, and targeted therapy. Chemotherapy was the earliest combination therapy with OV, but compared with others, the efficacy of this combination strategy is more controversial. Although several clinical trials have reported encouraging results,^333,346–349^ others with large sample sizes have found the combination strategy to be ineffective.^350,351^ For example, Eigl et al.^350^ found that the median survival time of patients with metastatic castration-resistant prostate cancer who received a combination docetaxel plus pelareorep was 19.1 months, while patients receiving docetaxel monotherapy had a median survival time of 21.1 months. It was suggested that the combination of OV and chemotherapy exhibited a “1 + 1 < 1” effect. However, Jonker et al.^351^ found that the combination of pelareorep with FOLFOX/Bevacizumab was tolerable with an increased ORR, but PFS was inferior.

The combination of OV and immunotherapy is currently the most concerning combination strategy. In 2017, Ribas et al. reported the results of their phase Ib clinical trial. The ORR of T-VEC combined with pembrolizumab in patients with advanced melanoma was as high as 62%, and the CR reached 33%.^318^ This encouraging result also confirmed that OV combined with ICIs has extremely broad application prospects. The final results of the KEYNOTE-034 study will be announced in 2022.^352^ Unfortunately, T-VEC with pembrolizumab failed to significantly enhance PFS or OS compared with placebo + pembrolizumab. The ORR was 48.6% for T-VEC + pembrolizumab and 41.3% for placebo + pembrolizumab. In another phase Ib clinical trial of T-VEC combined with ipilimumab in the treatment of melanoma, the combination therapy was also more effective than T-VEC or Ipilimumab monotherapy, with an ORR of 50%.^317^ In the updated results of the follow-up phase II clinical trial, 39% patients (38/98) in the combination arm and 18% patients (18/100) in the ipilimumab arm had an objective response (*P* = 0.02).^352^ In phase II clinical trial of T-VEC combined with pembrolizumab for locally advanced or metastatic sarcoma, the combined therapy also showed positive efficacy, with an overall ORR of 35%.^353^ Currently, a large number of clinical trials on the combination of OV and various immunotherapies are being carried out around the world, but most of the results are mainly reported in conferences, and the overall performance is encouraging. Among various combination strategies, the prospect of OV combined with immunotherapy is the most promising.

Shirakawa et al.^354^ reported the results of a phase I trial of OPB-301 combined with radiotherapy in the treatment of oesophageal cancer patients. The ORR reached 91.7%, and the combination strategy showed good safety. In contrast, in phase III clinical trial of JX-594 combined with sorafenib in the treatment of advanced HCC, the ORR of the combination group was only 19.2%, which was lower than the 20.9% of the sorafenib alone group. The study was terminated early because the OS endpoint was not reached. It is suggested that OV combined with targeted therapy has a high risk of failure. Notably, pelareorep combined with atezolizumab and chemotherapy (gemcitabine/nab-paclitaxel) has demonstrated encouraging results as first-line treatment in advanced or metastatic PDAC patients.^355^ The trial found that the ORR of patients reached 70%, which was almost three times (25%) the average ORR of the historical control, and the results are amazing. However, the trial did not report data on patient survival, such as median PFS, median OS, etc. The follow-up results deserve further attention. Although the overall efficacy of the three-drug combination is acceptable, its potential side effects also call for vigilance.

## Concluding remarks and future perspectives

From the accidental discovery of tumor shrinkage after infection with the natural virus to the widespread use of engineered OVs for targeted tumor therapy, OV therapy has gradually shown its powerful and magical antitumor ability, especially in solid tumors. It is not only a demon that can only invade and attack, but rather serve as a powerful tool of a genetically modified vector. It has displayed its targeting fidelity and antitumor immunity in many ways if it is properly harnessed. Our review classifies OVs in terms of variant functionalities, as well as comprehensively elaborates on the genetic engineering transformation of OVs regarding their functions and characteristics. Firstly, transforming the viruses into “qualified soldiers” so that they can selectively and safely destruct tumors. Next, equipping the “cold weapons” onto OVs improves the ability of replication and direct oncolysis. The following strategy focuses on arming the “hot weapons” to enhance the antitumor immunity of OVs, which is the most popular and critical perspective in OV modification. Finally, anti-angiogenesis, reversing metabolic reprogramming and ECM breakthrough can be effective auxiliary and novel weapons to elevate antitumor immune effect. These different engineering methods and ideas have provided references and directions for future research works on OV transformation research. Although a limited therapeutic effect was seen on OV monotherapy in the clinical, we summarized the combination results that incorporated OV treatment. Its collaboration with ICI provides the hope of curing tumors, and the combination with ACT even allows researchers to witness the most cutting-edge, top-notch, and most imaginative antitumor strategies. Finally, we summarize the progress of OV clinical trials, and put forward our thoughts and suggestions on the current trial results.

A perfectly versatile OV is still under inquisitive investigation. For example, capsid modification has been shown to enhance Ads infection but decrease the replication,^101^ improving HSV-2 replication but impairing the antitumor effect.^103^ Accordingly, it is expected to consider the balance of their function and the various characteristics of corresponding tumors. If the modification of the backbone enables the virus to replicate in large quantities in a short period of time and rapidly lyse tumor cells, and releasing adequate amount of transgenes while rapidly infecting new tumor cells, it would be an interesting point of OV-modification strategy in the future. Furthermore, a TTDR viral essential gene expression can increase both viral lytic activity and tumor specificity, and this provides a basis for the development of a novel tumor-specific OV for systemic treatment of locally advanced and metastatic prostate cancers.^108^

Another issue to be addressed is the intratumoral injection of OV, which is the most common delivery method for OV therapy. However, there are certain drawbacks associated with this method, including (1) the need for puncture to achieve intratumoral drug delivery, which poses a risk of bleeding and undesired metastasis at the lesion site; (2) technical difficulties in puncturing deep tumor tissues, which greatly reduces the number of applicable cases; and (3) requirement for skilled and experienced technicians to handle and administer the drug. Even though some naturally occurring OV, such as reovirus^356^ and alphavirus M1,^97,357^ are capable of being delivered through intravenous injection, the viruses in the circulation are at risk of being wiped out by the neutralizing antibodies. Also, the viral RNA only provides limited space for modification, making them less ideal for virus vector engineering. Shielding modifications by changing capsid, adding polymer coats,^358^ or enriching the extracellular envelope of OVs^359^ may apply to counteract. Another more feasible method is to achieve intravenous injection by encapsulating or loading OV onto special biomaterials. Unfortunately, the volume of nanoparticles is too small for OV encapsulation. In such cases, it is feasible to package the OVs with normal human cells and transfer those cells back to the patients.^360^ For example, there was a study using mesenchymal stem cells (MSCs) as the carrier for OV delivery, as they not only serve as the carrier, but also provide factories for producing virions, expressing additional transgenes, and modulating the immune system.^361–365^ In addition, due to the complex distributions of arteries, veins and bile ducts across the liver, underlying risks have to be carefully evaluated prior to intratumoral drug delivery to liver tumors. In this case, transarterial chemoembolization (TACE) is an effective solution.^366^ OV products are currently liquid preparations, which require high-cost frozen storage and cold chain transportation. Therefore, the development of lyophilized preparations of OVs is also in demand. Last but not least, the preclinical and clinical studies for potentially effective or limited clinical outcome OVs should focus on in-depth mechanisms.

Tumor heterogeneity appears to be an insurmountable obstacle for any existing antitumor treatments, and there is no exception for the OV treatment. Despite the fact that BiTE or TriTE innovatively assembled with OVs are very attractive, the combination medication strategy may be more advantageous in future research and development; however, 2/3 of current clinical studies have used monotherapy. The ideal combinations are not a simple superposition but rather a prudent consideration of the reagent options that functionally compensate for each other, and an accurate selection of targeting strategy by classification according to the type and stage of cancer, as well as the mechanism of each drug. For example, the hNIS gene inserted into CF-33^367^ promotes the opening of sodium iodide channels and facilitates I^129^ uptake by tumor cells, making CF-33 a natural coordinating synergist for radiotherapy. In another case, CAR-T/CAR-NK targets are carried by OVs, thereby increasing the tumor tropism of CAR-T/CAR-NK, and finally achieving a synergistic effect. The work of Priceman’s group that we have mentioned above reflects the view well,^298^ and the combination with CAR-NK is also worth further exploration. “Oncolytic virus-like” drugs that enhance the efficacy of other treatments are also a promising direction. With the advanced understanding of antitumor mechanisms on OVs and the related experiments carried out actively, the weapons depot for OVs will be comprehensively established so that different OV weapons and other antitumor therapies can be selected for individualized treatment. In short, OVs carry our expectations of personalized and precision medicine in the future.

## Supplementary information

S-table 1 Summary of global clinical trials
