# Supplementary material for: Oncolytic virotherapy: basic principles, recent advances and future directions
Source: Signal Transduct Target Ther. 2023 Apr 11;8:156. doi: 10.1038/s41392-023-01407-6 (PMC10090134; doi:10.1038/s41392-023-01407-6)
Supplement: Supplementary file 1 — S-table 1 Summary of global clinical trials [file 41392_2023_1407_MOESM1_ESM.docx]

S-table 1 Summary of global clinical trials

| Identifier | Tittle | Interventions | Status | Phases | Indications | Drug targets |
| --- | --- | --- | --- | --- | --- | --- |
| NCT02140996 | Ad-sig-hMUC-1/ecdCD40L Vector Vaccine for Immunotherapy of Epithelial Cancers | OV: Ad-sig-hMUC-1/ecdCD40L vector vaccine | Unknown | I | Epithelial Cancers of the Lung, Breast, Ovary, Prostate and Colon | CD40 |
| NCT01017601 | A Randomized Double-Blinded Phase II Study of NTX-010, a Replication-Competent Picornavirus, After Standard Platinum-Containing Cytoreductive Induction Chemotherapy in Patients With Extensive Stage Small Cell Lung Cancer | OV: NTX-010;  Other: Placebo | Terminated | II | Lung Cancer | ANTXR1 |
| NCT04391049 | Phase I Trial With Expansion Cohort of OBP-301 (Telomelysin™) and Definitive Chemoradiation for Patients With Locally Advanced Esophageal and Gastroesophageal Cancer Who Are Not Candidates for Surgery | OV: OBP-301;  Other: Carboplatin, Paclitaxel, Radiation | Recruiting | I | Solid Tumor | TERT |
| NCT03015922 | VIRel: Viral Immunotherapy in Relapsed/Refractory Multiple Myeloma - A Phase I Study to Assess the Safety and Tolerability of REOLYSIN® (Pelareorep) in Combination With Lenalidomide or Pomalidomide | OV: Pelareorep; Other: Lenalidomide or Pomalidomide | Unknown | I | Multiple Myeloma | -，IKZF3、IKZF1、CSNK1A1，IKZF3、IKZF1 |
| NCT05234905 | Oncolytic Virus(H101) Combined With Camrelizumab for Recurrent Cervical Cancer: a Prospective, Multicenter Study | OV: Ad5;  Other: Camrelizumab | Not yet recruiting | II | Uterine Cervical Neoplasms | - |
| NCT00788307 | Phase I Trial of In Situ Gene Therapy for Locally Recurrent Prostate Cancer Following Radiation Therapy Failure Using Sodium/Iodide Symporter and Radioiodine | OV: Ad5-CMV-NIS; Radiation | Terminated | I | Prostate Cancer | - |
| NCT00003167 | A Phase I Trial of Intravesical Ad-p53 Treatment in Locally Advanced and Metastatic Bladder Cancer | OV: Ad5CMV-p53 | Completed | I | Recurrent Bladder Cancer，Stage I Bladder Cancer，Stage II Bladder Cancer，Stage III Bladder Cancer，Stage IV Bladder Cancer，Transitional Cell Carcinoma of the Bladder | p53 |
| NCT00003649 | Phase I Pilot Trial of Adenovirus p53 in Bronchioloalveolar Cell Lung Carcinoma (BAC) Administered by Bronchoalveolar Lavage | OV: Ad5CMV-p53 | Completed | I | Lung Cancer | p53 |
| NCT00003147 | Phase I Study of Percutaneous Injections of Adeno-Virus p53 Construct (ADENO-p53) for Hepatocellular Carcinoma | OV: Ad5CMV-p53 | Terminated | I | Liver Cancer | p53 |
| NCT00004225 | Phase I Pilot Trial of Adenovirus p53 and Radiotherapy on Nonsmall Cell Lung Cancer | OV: Ad5CMV-p53;  Other: Radiation | Completed | I | Lung Cancer | p53 |
| NCT00004041 | Phase I Trial of Adenovirus-Mediated Wild-Type P53 Gene Therapy for Malignant Gliomas | OV: Ad5CMV-p53;  Other: Surgery | Completed | I | Brain and Central Nervous System Tumors | p53 |
| NCT00003588 | A Phase I Study of Ad-p53 (NSC# 683550) for Patients With Platinum- and Paclitaxel-Resistant Epithelial Ovarian Cancer | OV: Ad5CMV-p53;  Other: Surgery | Completed | I | Ovarian Cancer | p53 |
| NCT03896568 | Phase I Clinical Trial of Allogeneic Bone Marrow Human Mesenchymal Stem Cells Loaded With A Tumor Selective Oncolytic Adenovirus, DNX-2401, Administered Via Intra-Arterial Injection in Patients With Recurrent High-Grade Glioma | OV: Ad5-DNX-2401;  Other: Therapeutic Conventional Surgery | Recruiting | I | IDH1 wt Allele，Recurrent Anaplastic Astrocytoma，Recurrent Glioblastoma，Recurrent Gliosarcoma，Recurrent Malignant Glioma | Integrin αvβ5、Integrin αvβ3 |
| NCT02762045 | Phase I Clinical Trial of Recombinant Adenovirus Type 5 Therapeutic AIDS Vaccine Expressing Gag | OV: Ad5-gag;  Other: Placebo | Unknown | I | Acquired Immunodeficiency Syndrome | - |
| NCT02894944 | Clinical Trial Phase I for Evaluation of Tolerability and Safety of Replication-competent Adenovirus-mediated Double Suicide Gene Therapy in Combination With Chemotherapy for the Locally Advanced Pancreatic Cancer | OV: Ad5-yCD/mutTKSR39rep-ADP | Completed | I | Pancreatic Cancer | - |
| NCT00415454 | Phase I Study Combining Replication-Competent Adenovirus-Mediated Suicide Gene Therapy With Chemoradiotherapy for the Treatment of Non-Metastatic Pancreatic Adenocarcinoma | OV: Ad5-yCD/mutTKSR39rep-ADP | Terminated | I | Pancreatic Cancer | - |
| NCT03029871 | Phase 1 Trial of Oncolytic Adenovirus-Mediated Cytotoxic Gene Therapy in Combination With Stereotactic Body Radiation Therapy (SBRT) in Clinical Stage 1/11A (T1A-T2B) Non-Small Cell Lung Cancer | OV: Ad5-yCD/mutTKSR39rep-ADP Adenovirus | Terminated | I | Non-small Cell Lung Cancer Stage I | - |
| NCT03281382 | PHASE 1 TRIAL OF ONCOLYTIC ADENOVIRUS-MEDIATED CYTOTOXIC AND IL-12 GENE THERAPY IN COMBINATION WITH CHEMOTHERAPY FOR THE TREATMENT OF METASTATIC PANCREATIC CANCER | OV: Ad5-yCD/mutTKSR39rep-hIL12 | Completed | I | Metastatic Pancreatic Cancer | - |
| NCT02555397 | Phase 1 Trial of Oncolytic Adenovirus-Mediated Cytotoxic and Interleukin 12 Gene Therapy for Locally Recurrent Prostate Cancer After Definitive Radiotherapy | OV: Ad5-yCD/mutTKSR39rep-hIL12 | Active, not recruiting | I | Prostate Cancer | -，- |
| NCT04673942 | A Phase I, First in Human, Study to Evaluate the Safety and Tolerability of AdAPT-001 in Subjects With Refractory Solid Tumors | OV: AdAPT-001 | Recruiting | I | Solid Tumor | - |
| NCT00850057 | A Phase 1, Open Label, Dose-escalation, Pharmacodynamic Study of Intranodal Injection of Adenovirus-CD154 (Ad-ISF35) in Patients With Chronic Lymphocytic Leukemia/Small Lymphocytic Lymphoma | OV: Ad-ISF35 | Completed | I | Chronic Lymphocytic Leukemia，Small Lymphocytic Lymphoma | CD40L |
| NCT03544723 | A Phase 2, Multi-Center, Open Label Study to Evaluate Adenoviral p53 (Ad-p53) in Combination With Immune Checkpoint Inhibitor Therapy in Patients With Recurrent or Metastatic Head and Neck Squamous Cell Carcinoma and Other Tumors Approved for Anti-PD-1 or Anti-PD-L1 Therapy | OV: Ad-p53 | Recruiting | II | Solid Tumor，Lymphoma | p53 |
| NCT01869088 | Phase Ⅲ Trial of Transcatheter Arterial Chemoembolization(TACE) Plus Recombinant Human Adenovirus Type 5 Injection for Unresectable Hepatocellular Carcinoma (HCC) | OV: Ad-p53;  Other: Trans-catheter embolization | Recruiting | III | Hepatocellular Carcinoma | - |
| NCT02842125 | Phase 1/2 Evaluation of Adenoviral p53 (Ad-p53) in Combination With Capecitabine (Xeloda) or Anti-PD-1 in Patients With Unresectable Liver Metastases of Colorectal Carcinoma(CRC) and Other Solid Tumors, Recurrent Head and Neck Squamous Cell Carcinoma (HNSCC) and Primary Hepatic Cancers With Known Disease Progression on Standard Therapy | OV: Ad-P53;  Other: Xeloda, Keytruda, Opdivo | Terminated | I/II | Metastatic Solid Tumor Cancer，Recurrent Head and Neck Cancer | TYMS，PD-1，PD-1，p53 |
| NCT02749331 | Study of Recombinant Adenovirus (AdVince) in Patients With Neuroendocrine Tumors; Safety and Efficacy | OV: AdVince | Recruiting | I/II | Neuroendocrine Tumors | - |
| NCT01997190 | A Phase I Study of Intrapleural AdV-tk Therapy in Patients With Malignant Pleural Effusion | OV: AdV-tk | Completed | I | Malignant Pleural Effusion，Lung Cancer，Mesothelioma，Breast Cancer，Ovarian Cancer | - |
| NCT02768363 | A Randomized Controlled Trial Of AdV-tk + Valacyclovir Administered During Active Surveillance For Newly Diagnosed Prostate Cancer | OV: AdV-tk + Valacyclovir;  Other: Valacyclovir, Placebo | Active, not recruiting | II | Prostate Cancer | DNA Pol，- |
| NCT00751270 | A Phase 1b Study of AdV-tk + Valacyclovir Gene Therapy in Combination With Standard Radiation Therapy for Malignant Gliomas | OV: AdV-tk;  Other: Valacyclovir | Completed | I | Malignant Glioma，Glioblastoma Multiforme，Anaplastic Astrocytoma | - |
| NCT00638612 | AdV-tk + Valacyclovir Therapy in Combination With Surgery and Chemoradiation for Pancreas Cancer | OV: AdV-tk;  Other: Valacyclovir | Completed | I | Pancreatic Adenocarcinoma | DNA Pol，- |
| NCT03131037 | Intratumoral Gene Mediated Cytotoxic Immunotherapy (GMCI) For Resectable Non-Small Cell Lung Cancer | OV: AdV-tk;  Other: Valacyclovir | Active, not recruiting | I | Lung Cancer | -，DNA Pol |
| NCT00589875 | A Phase 2a Study of AdV-tk + Valacyclovir Gene Therapy in Combination With Standard Radiation Therapy for Malignant Glioma | OV: AdV-tk;  Other: Valacyclovir | Completed | II | Malignant Glioma，Glioblastoma Multiforme，Anaplastic Astrocytoma | - |
| NCT00634231 | A Phase I Study of AdV-tk + Prodrug Therapy in Combination With Radiation Therapy for Pediatric Brain Tumors | OV: AdV-tk;  Other: Valacyclovir, Radiation | Completed | I | Malignant Glioma，Recurrent Ependymoma | -，DNA Pol |
| NCT03576612 | Phase I Study of Neoadjuvant GMCI Plus Immune Checkpoint Inhibitor Combined With Standard of Care for Newly Diagnosed High-Grade Gliomas | OV: AdV-tk;  Other: Valacyclovir, Radiation, Temozolomide, Nivolumab | Active, not recruiting | I | Glioma, Malignant | PD-1，-，DNA Pol，- |
| NCT04758533 | Phase IB Clinical Trial to Assess the Safety, Tolerability, and Preliminary Efficacy of AloCELYVIR (Mesenchymal Allogenic Cells + ICOVIR-5) in Children, Adolescent and Young Adults With Newly Diagnosed Diffuse Intrinsic Pontine Glioma (DIPG) in Combination With Radiotherapy or Medulloblastoma in Relapse/Progression in Monotherapy | OV: AloCELYVIR | Recruiting | I/II | Diffuse Intrinsic Pontine Glioma，Medulloblastoma, Childhood, Recurrent | - |
| NCT05047276 | Phase I/II Study of AloCelyvir in Patients With Metastatic Uveal Melanoma | OV: AloCelyvir | Not yet recruiting | I/II | Uveal Melanoma, Metastatic | - |
| NCT03954067 | A Phase 1, Open-label Study of ASP9801, an Oncolytic Virus, Administered by Intratumoral Injection as a Single Agent and in Combination With Pembrolizumab in Subjects With Advanced/Metastatic Solid Tumors | OV: ASP9801;  Other: Pembrolizumab | Recruiting | I | Solid Tumor | IL7R、IL12R |
| NCT05155332 | Phase I Open-label, Dose Escalation Trial of BI 1831169 Monotherapy and in Combination With Ezabenlimab in Patients With Advanced or Metastatic Solid Tumors | OV: BI 1831169;  Other: Ezabenlimab | Recruiting | I | Solid Tumor | -，PD-1 |
| NCT05393440 | A First-in-human Phase I Two-stage Clinical Trial for Intratumoral Injection of Recombinant Oncolytic Type II Herpes Simplex Virus (BS-006) in Patients With Recurrent Cervical Cancer | OV: BS-006 | Not yet recruiting | I | Uterine Cervical Neoplasms | CD3、PD-L1 |
| NCT04725331 | A Phase I/IIa Study of Intra-tumoral BT-001 (TG6030) Administered Alone and in Combination With Pembrolizumab in Patients With Cutaneous or, Subcutaneous Lesions or Easily Injectable Lymph Nodes of Metastatic/Advanced Solid Tumors. | OV: BT-001;  Other: Pembrolizumab | Recruiting | I/II | Solid Tumor | CTLA4、CSF2R，PD-1 |
| NCT03657576 | A Phase I Trial of IRS-1 HSV C134 Administered Intratumorally in Patients With Recurrent Malignant Glioma | OV: C134 | Active, not recruiting | I | Glioblastoma Multiforme of Brain，Anaplastic Astrocytoma of Brain，Gliosarcoma of Brain | - |
| NCT05095441 | A Phase 1 Open-Label Study of Genetically Engineered Oncolytic HSV-1 (C5252) Expressing IL-12 and Anti-PD-1 Antibody in Patients With Recurrent or Progressive Glioblastoma | OV: C5252 | Not yet recruiting | I | Solid Tumor，Glioblastoma，Glioblastoma Multiforme，Glioblastoma Multiforme of Brain | PD-1、IL12R |
| NCT03740256 | A First in Human Phase I Trial of Binary Oncolytic Adenovirus in Combination With HER2-Specific Autologous CAR T Cells in Patients With Advanced HER2 Positive Solid Tumors | OV: CAdVEC | Recruiting | I | Bladder Cancer，Head and Neck Squamous Cell Carcinoma，Cancer of the Salivary Gland，Lung Cancer，Breast Cancer，Gastric Cancer，Esophageal Cancer，Colorectal Cancer，Pancreatic Adenocarcinoma，Solid Tumor | HER2 |
| NCT04495153 | CAN-2409 Plus Prodrug With Standard of Care Immune Checkpoint Inhibitor for Stage III/IV NSCLC Patients | OV: CAN-2409 | Recruiting | II | Non Small Cell Lung Cancer | - |
| NCT02565992 | Phase I Study of Intratumoral CAVATAK® (Coxsackievirus A21) and Pembrolizumab in Subjects With Advanced Melanoma (VLA-011 CAPRA) | OV: CAVATAK;  Other: Pembrolizumab | Completed | I | Melanoma | ICAM1、CD55，PD-1 |
| NCT01844661 | Phase 1 Trial of Celyvir in Children and Adults With Metastatic and Refractory Solid Tumors. | OV: CELYVIR | Completed | I/II | Solid Tumor | - |
| NCT05081492 | A Phase I, First-in-Human Study of Intratumoral Administration of CF33-hNIS-antiPDL1, A Novel Chimeric Oncolytic Poxvirus Encoding Human Sodium Iodide Symporter (HNIS) in Patients With Metastatic Triple Negative Breast Cancer | OV: CF33 | Recruiting | I | Anatomic Stage IV Breast Cancer，Metastatic Triple-Negative Breast Carcinoma，Prognostic Stage IV Breast Cancer | PD-L1 |
| NCT05346484 | A Phase I, Dose Escalation Safety and Tolerability Study of VAXINIA (CF33-hNIS), Administered Intratumorally or Intravenously as a Monotherapy or in Combination With Pembrolizumab in Adult Patients With Metastatic or Advanced Solid Tumors (MAST). | OV: CF33;  Other: Pembrolizumab | Recruiting | I | Solid Tumor | PD-1，- |
| NCT00109655 | A Phase 1 Dose-Escalation Trial of Intravesical CG0070 for Superficial Transitional Cell Carcinoma of the Bladder After Bacillus Calmette-Guerin Failure | OV: CG0070 | Unknown | I | Carcinoma, Transitional Cell，Bladder Neoplasms | - |
| NCT02143804 | Phase II Safety and Efficacy Study of CG0070 Oncolytic Vector Regimen in Patients With High Grade Non-Muscle Invasive Bladder Carcinoma Disease (NMIBC) Who Have Failed BCG and Refused Cystectomy | OV: CG0070 | Terminated | II | Bladder Cancer，High Grade，Non Muscle Invasive | CD55、TNFSF10 |
| NCT02365818 | An Open Label, Single Arm, Phase II, Multicenter Study of the Safety and Efficacy of CG0070 Oncolytic Vector Regimen in Patients With Non-Muscle Invasive Bladder Carcinoma Who Have Failed BCG (Bacillus Calmette-Guerin) Therapy and Refused Cystectomy | OV: CG0070 | Completed | II | Bladder Cancer | - |
| NCT01438112 | An Integrated Phase II/III, Open Label, Randomized and Controlled Study of the Safety and Efficacy of CG0070 Adenovirus Vector Expressing GM-CSF in Patients With NMIBC With Carcinoma In Situ Disease Who Have Failed BCG | OV: CG0070 | Terminated | II/III | Transitional Cell Carcinoma，Bladder Cancer，Carcinoma in Situ，Carcinoma in Situ Concurrent With Papillary Tumors | - |
| NCT04452591 | A Phase 3 Study of CG0070 in Patients With Non-Muscle Invasive Bladder Cancer (NMIBC) Unresponsive to Bacillus-Calmette-Guerin (BCG) | OV: CG0070;  Other: n-dodecyl-B-D-maltoside | Recruiting | III | Non Muscular Invasive Bladder Cancer | - |
| NCT04610671 | A Phase 1 Study of CG0070 Combined With Nivolumab in Cisplatin Ineligible Patients With Muscle Invasive Bladder Cancer (MIBC) | OV: CG0070;  Other: Nivolumab | Recruiting | I | Bladder Cancer | -，PD-1 |
| NCT04387461 | A Phase 2, Single Arm Study of CG0070 Combined With Pembrolizumab in Patients With Non Muscle Invasive Bladder Cancer (NMIBC) Unresponsive to Bacillus Calmette-Guerin (BCG) | OV: CG0070;  Other: Pembrolizumab, n-dodecyl-B-D-maltoside | Active, not recruiting | II | Non Muscle Invasive Bladder Cancer | -，PD-1 |
| NCT00116155 | A Phase I/II Dose Finding Trial of the Intravenous Injection of CV787, a Prostate-Specific Antigen Cytolytic Adenovirus, in Patients With Hormone Refractory Metastatic Prostate Cancer | OV: CG7870 | Completed | I/II | Prostate Cancer | - |
| NCT00103428 | A Phase 1/2a Dose-Escalation Trial of Intravenous CG7870 in Combination With Docetaxel in Chemotherapy-Naïve Patients With Metastatic Hormone-Refractory Prostate Cancer | OV: CG7870 | Terminated | I/II | Prostate Cancer | -，Tubulin |
| NCT01437280 | GOAT; Phase I Single-Center Open Label Dose Escalation Study of CGTG-102, a GM-CSF Encoding Oncolytic Adenovirus, for Therapy of Advanced Cancers | OV: CGTG-102 | Terminated | I | Solid Tumor | CSF2R、DSG2、CD46 |
| NCT02053220 | A Phase 1 Clinical Study of Intra-tumoural Injection or Intravenous Infusion of a Group B Oncolytic Adenovirus (ColoAd1) in Patients With Cancer Who Are Candidates for Resection of Primary Tumour | OV: Colo-Ad1 | Completed | I | Resectable Colon Cancer，Resectable Non-small Cell Lung Cancer，Resectable Bladder Cancer，Resectable Renal Cell Carcinoma | - |
| NCT00438009 | A Phase I, Open Label, Cohort Study of Two Doses of Cavatak (Coxsackievirus Type A21) Given Intratumourally in Stage IV Melanoma Patients. | OV: Coxsackievirus A21 | Completed | I | Stage IV Melanoma | ICAM1、CD55 |
| NCT00235482 | Intratumoural Administration of Coxsackievirus A21 for the Control of Malignant Melanoma (PXS-X02) | OV: Coxsackievirus A21 | Completed | I | Malignant Melanoma | ICAM1、CD55 |
| NCT05139056 | A Phase I Study of Multiple Doses of Neural Stem Cell-Based Oncolytic Virotherapy (NSC-CRAd-S-pk7) Administered Intracerebrally to Patients With Recurrent High-Grade Gliomas | OV: CRAd-S-pk7 | Not yet recruiting | I | Recurrent Anaplastic Astrocytoma，Recurrent Anaplastic Oligoastrocytoma，Recurrent Anaplastic Oligodendroglioma，Recurrent Glioblastoma，Recurrent Gliosarcoma，Recurrent Malignant Glioma，Recurrent WHO Grade II Glioma，Recurrent WHO Grade III Glioma | - |
| NCT00636558 | A Phase I, Open-Label, Cohort Study of Multiple Doses of Cavatak™ (Coxsackie Virus A21) Given Intravenously to Stage IV Solid Tumour Cancer Patients Bearing ICAM-1 With or Without DAF Expressing Tumours (PSX-X04) | OV: CVA21 | Completed | I | Melanoma，Breast Cancer，Prostate Cancer | ICAM1、CD55 |
| NCT00832559 | A Phase I, Open-label, Dosage Escalation, Study of Multiple Doses of CAVATAKTM (CVA21; Coxsackievirus A21) Administered Intratumourally in the Treatment of Squamous Cell Carcinoma of the Head and Neck Bearing ICAM-1 Receptors (VLA-X06) | OV: CVA21 | Terminated | I | Head and Neck Cancer | ICAM1、CD55 |
| NCT01636882 | A Phase 2 Study of the Efficacy and Safety of Intratumoral CAVATAK™ (Coxsackievirus A21, CVA21) in Patients With Stage IIIc and Stage IV Malignant Melanoma to Extend Dosing for up to 48 Weeks Total (VLA-008 CALM Ext) | OV: CVA21 | Completed | II | Melanoma | ICAM1、CD55 |
| NCT01227551 | A Phase 2 Study of the Efficacy and Safety of Intratumoral CAVATAK™ (Coxsackievirus A21, CVA21) in Patients With Stage IIIc and Stage IV Malignant Melanoma (VLA-007 CALM ) | OV: CVA21 | Completed | II | Melanoma | ICAM1、CD55 |
| NCT03408587 | An Open-Label Phase 1b Clinical Study of Intravenous CAVATAK® (Coxsackievirus A21, CVA21), in Combination With Ipilimumab in Subjects With Uveal Melanoma Metastatic to Liver (VLA-024 CLEVER) | OV: CVA21;  Other: Ipilimumab | Completed | I | Uveal Melanoma，Liver Metastases | ICAM1、CD55，CTLA4 |
| NCT02316171 | A Phase 1 Study to Evaluate the Safety and Clinical Activity of Intravesicular CAVATAK (Coxsackievirus A21, CVA21) Alone and in Sequential Combination With Low Dose Mitomycin C in Patients With Non-Muscle Invasive Bladder Cancer (VLA-012 CANON) | OV: CVA21;  Other: Mitomycin C | Completed | I | Non-muscle Invasive Bladder Cancer | ICAM1、CD55 |
| NCT02043665 | A Phase 1, Dose-finding and Signal-seeking Study of the Safety & Efficacy of Intravenous CAVATAK® Alone and in Combination With Pembrolizumab in Patients With Late Stage Solid Tumours (VLA-009 STORM / KEYNOTE-200) | OV: CVA21;  Other: Pembrolizumab | Completed | I | Non-small Cell Lung Cancer，Bladder Cancer | ICAM1、CD55，PD-1 |
| NCT02824965 | A Phase I/II Open-label Trial of Intravenous CAVATAK^TM in Combination With Pembrolizumab for the Treatment of Patients With Advanced NSCLC | OV: CVA21;  Other: Pembrolizumab | Active, not recruiting | I | Non-Small Cell Lung Cancer | ICAM1、CD55，PD-1 |
| NCT03514836 | A Phase I/II, Clinical Trial to Evaluate the Safety and Immune Activation of the Combination of DCVAC/PCa, and ONCOS-102, in Men With Advanced Metastatic Castration-resistant Prostate Cancer. | OV: DCVac/Pca;  Other: Cyclophosphamide | Terminated | I/II | Castration-resistant Prostate Cancer | CSF2R、DSG2、CD46 |
| NCT03178032 | Phase I Trial of DNX-2401 for Diffuse Intrinsic Pontine Glioma Newly Diagnosed in Pediatric Patients. | OV: DNX-2401 | Unknown | I | Brainstem Glioma | Integrin αvβ5、Integrin αvβ3 |
| NCT02197169 | A Phase 1b, Randomized, Multi-center, Open-label Study of a Conditionally Replicative Adenovirus (DNX-2401) and Interferon Gamma (IFN-γ) for Recurrent Glioblastoma or Gliosarcoma (TARGET-I) | OV: DNX-2401;  Other: Interferon-gamma | Completed | I | Glioblastoma or Gliosarcoma | Integrin αvβ5、Integrin αvβ3 |
| NCT02798406 | A Phase II, Multi-center, Open-label Study of a Conditionally Replicative Adenovirus (DNX-2401) With Pembrolizumab (KEYTRUDA®) for Recurrent Glioblastoma or Gliosarcoma (CAPTIVE/KEYNOTE-192) | OV: DNX-2401;  Other: Pembrolizumab | Completed | II | Brain Cancer，Brain Neoplasm，Glioma，Glioblastoma，Gliosarcoma，Malignant Brain Tumor，Neoplasm, Neuroepithelial，Neuroectodermal Tumors，Neoplasm by Histologic Type，Neoplasm, Nerve Tissue，Nervous System Diseases | PD-1，Integrin αvβ5、Integrin αvβ3 |
| NCT00805376 | Phase I Trial of Conditionally Replication-Competent Adenovirus (DNX-2401, Formerly Known as Delta-24-RGD-4C) for Recurrent Malignant Gliomas | OV: DNX-2401;  Other: Surgery | Completed | I | Brain Cancer，Central Nervous System Diseases | Integrin αvβ5、Integrin αvβ3 |
| NCT01956734 | Phase I Trial of Combination of DNX-2401 (Formerly Named Delta-24-RGD) Oncolytic Adenovirus With a Short Course of Temozolomide for Treatment of Glioblastoma at First Recurrent | OV: DNX2401;  Other: Temozolomide | Completed | I | Glioblastoma Multiforme，Recurrent Tumor | Integrin αvβ5、Integrin αvβ3，- |
| NCT04714983 | A Phase I Safety and Window-of-opportunity Study of Preoperative Intratumoral Injection of OX40-ligand Expressing Oncolytic Adenovirus (DNX-2440) in Patients With Resectable Liver Metastasis | OV: DNX-2440 | Recruiting | I | Liver Metastases，Liver Metastasis Colon Cancer，Colorectal Cancer，Breast Cancer，Gastric Cancer，Periampullary Cancer，Melanoma，Renal Cell Cancer，Sarcoma，Squamous Cell Carcinoma，Gastrointestinal Stromal Tumors | Integrin αvβ5、Integrin αvβ3 |
| NCT03714334 | Phase I Trial of DNX-2440 Oncolytic Adenovirus in Patients With Recurrent Glioblastoma | OV: DNX-2440 | Recruiting | I | Glioblastoma，Glioblastoma, Adult | Integrin αvβ5、Integrin αvβ3 |
| NCT02028117 | A Clinical Study Of Enadenotucirev: Dose Finding and Proof of Concept in Platinum-Resistant Epithelial Ovarian Cancer. | OV: Enadenotucirev | Completed | I | Recurrent Platinum Resistant Ovarian Cancer | - |
| NCT02028442 | A Clinical Study Of Enadenotucirev Administered by Sub-Acute Fractionated Intravenous Injection: Dose Escalation in Metastatic Epithelial Solid Tumours and Randomised Controlled Trial in Metastatic Colorectal Cancer | OV: Enadenotucirev | Completed | I/II | Solid Tumours of Epithelial Origin，Metastatic Colorectal Cancer，Metastatic Bladder Cancer | - |
| NCT03916510 | A Phase 1 Trial of the Safety, Tolerability and Biological Effects of Intravenous Enadenotucirev, a Novel Oncolytic Virus, in Combination With Chemoradiotherapy in Locally Advanced Rectal Cancer | OV: Enadenotucirev;  Other: Capecitabine, Radiation | Recruiting | I | Locally Advanced Rectal Cancer | - |
| NCT02636036 | A Phase I Multicenter, Open Label Study of Enadenotucirev Combined With PD-1 Inhibitor in Subjects With Metastatic or Advanced Epithelial Tumors | OV: Enadenotucirev;  Other: Nivolumab | Completed | I | Colorectal Cancer，Squamous Cell Carcinoma of the Head and Neck，Epithelial Tumor | - |
| NCT03911388 | Phase 1 Trial of Engineered HSV G207 in Children With Recurrent or Refractory Cerebellar Brain Tumors | OV: G207 | Recruiting | I | Solid Tumor | - |
| NCT00157703 | A Staged Phase 1 Study of the Treatment of Malignant Glioma With G207, a Genetically Engineered HSV-1, Followed by Radiation Therapy | OV: G207 | Completed | I | Malignant Glioma | - |
| NCT02457845 | Phase I Clinical Trial of HSV G207 Alone or With a Single Radiation Dose in Children With Recurrent Supratentorial Brain Tumors | OV: G207 | Active, not recruiting | I | Supratentorial Neoplasms, Malignant，Malignant Glioma，Glioblastoma，Anaplastic Astrocytoma，PNET，Cerebral Primitive Neuroectodermal Tumor，Embryonal Tumor | - |
| NCT00028158 | An Open-Label Phase Ib/II Study of the Safety, Tolerability and Efficacy of G207, a Genetically Engineered Herpes Simplex Type-1 Virus, Administered Intracerebrally to Patients With Recurrent Malignant Glioma | OV: G207 | Completed | I/II | Glioma，Astrocytoma，Glioblastoma | - |
| NCT04482933 | Phase II Clinical Trial of HSV G207 With a Single 5 Gy Radiation Dose in Children With Recurrent High-Grade Glioma | OV: G207 | Not yet recruiting | II | Neoplasms，High Grade Glioma，Glioblastoma Multiforme，Malignant Glioma of Brain，Anaplastic Astrocytoma of Brain，High-grade Glioma，Anaplastic Glioma，Giant Cell Glioblastoma | - |
| NCT01584284 | Phase I Trial Of Attenuated Vaccinia Virus (GL-ONC1) Delivered Intravenously With Concurrent Cisplatin and Radiotherapy in Patients With Locoregionally Advanced Head and Neck Carcinoma | OV: GL-ONC1 | Completed | I | Cancer of Head and Neck | - |
| NCT00794131 | Phase I Study of the Safety, Tolerability,and Tumor-Specific Replication of the Intravenous Administration of Green Fluorescent Protein Encoded Genetically Engineered Attenuated Vaccinia Virus, GL-ONC1, in Patients With Advanced Solid Organ Cancers. | OV: GL-ONC1 | Completed | I | Solid Tumors | - |
| NCT02714374 | An Open Label, Non-randomized Phase 1b Study to Investigate the Safety and Effect of the Oncolytic Virus GL-ONC1 Administered Intravenously Prior to Surgery to Patients With Solid Organ Cancers Undergoing Surgery for Curative-Intent or Palliative Resection | OV: GL-ONC1 | Terminated | I | Solid Tumor | - |
| NCT01766739 | Phase I Study of Intra-pleural Administration of GL-ONC1, a Genetically Modified Vaccinia Virus, in Patients With Malignant Pleural Effusion: Primary, Metastases and Mesothelioma | OV: GL-ONC1 | Active, not recruiting | I | Lung Cancer | - |
| NCT01443260 | Phase I/II Study of Intraperitoneal Administration of GL-ONC1, a Genetically Modified Vaccinia Virus, in Patients With Peritoneal Carcinomatosis | OV: GL-ONC1 | Completed | I/II | Peritoneal Carcinomatosis | - |
| NCT02759588 | Phase 1b & 2 Study With GL-ONC1 Oncolytic Immunotherapy in Patients With Recurrent or Refractory Ovarian Cancer (VIRO-15) | OV: GL-ONC1;  Other: Chemotherapy, Bevacizumab | Active, not recruiting | I/II | Ovarian Cancer，Peritoneal Carcinomatosis，Fallopian Tube Cancer | - |
| NCT02446093 | Neoadjuvant GMCI Plus Chemoradiation for Advanced Non-Metastatic Pancreatic Adenocarcinoma | OV: GMCI (aglatimagene besadenovec + valacyclovir);  Other: Chemotherapy, Radiation, Surgery | Recruiting | II | Pancreatic Adenocarcinoma | DNA Pol，- |
| NCT04771676 | A Phase II Study of Intraperitoneal Injection of Oncolytic Viruses H101 for Patients With Refractory Malignant Ascites | OV: H101 | Recruiting | II | Refractory Malignant Ascites | - |
| NCT02579564 | Systemic Chemotherapy Combined With Thoracic Cavity Perfusion of Recombinant Human Adenovirus Type 5 and Endostatin Injections Versus Cisplatin for Treatment Malignant Hydrothorax in Non Small Cell Lung Cancer (NSCLC) Patients: A Multi-center, Randomized, Controlled Clinical Trial | OV: H101：  Other: Gemcitabine, Vinorelbine, Paclitaxel, Pemetrexed, Endostar, Cisplatin | Recruiting | III | Malignant Hydrothorax，Non Small Cell Lung Cancer | RNR，Tubulin-β、Tubulin-α，Tubulin，DHFR、GART，-，Endostatin，- |
| NCT05564897 | Phase II Single Center Open-Label Single-Arm Study of the Safety and Efficacy of Oncolytic Adenovirus H101 Combined With PD-1 Inhibitor in Patients With Non-muscle-invasive Bladder Cancer Who Failed BCG Therapy | OV: H101;  Other: Camrelizumab | Recruiting | II | Bladder Cancer | -，PD-1 |
| NCT03780049 | Hepatic Artery Infusion Chemotherapy Plus Recombinant Human Type-5 Adenovirus vs Hepatic Artery Infusion Chemotherapy Alone for Unresectable Hepatocellular Carcinoma at Barcelona Clinic Liver Cancer A-B Stage | OV: H101;  Other: HAIC of FOLFOX, Placebos | Recruiting | III | Hepatocellular Carcinoma | - |
| NCT05303844 | A Phase 1b Dose-escalation and Cohort-expansion Study of the Safety/Tolerability, and Efficacy of Oncolytic Virotherapy Plus PD-1 Inhibitor for Patients With Refractory Malignant Ascites (OPTIONS-02) | OV: H101;  Other: Tislelizumab | Recruiting | I | Refractory Malignant Ascites | -，PD-1 |
| NCT05303090 | A Phase 1b Dose-escalation and Cohort-expansion Study of the Safety/Tolerability, and Efficacy of Oncolytic Virotherapy Plus PD-1 Inhibitor and Lenvatinib for Patients With Advanced Pancreatic Cancer | OV: H101;  Other: Tislelizumab, lenvatinib | Recruiting | I | Pancreatic Ductal Adenocarcinoma | -，PD-1，PDGFA、KIT、RET、VEGFR、FGFR |
| NCT02653313 | A Non-controlled, Single Arm, Open Label, Phase II Study of Intravenous and Intratumoral Administration of ParvOryx in Patients With Metastatic, Inoperable Pancreatic Cancer | OV: H-1PV | Completed | I/II | Carcinoma, Pancreatic Ductal | - |
| NCT01301430 | Phase I/IIa Study of Intratumoral/Intracerebral or Intravenous/Intracerebral Administration of Parvovirus H-1 (ParvOryx) in Patients With Progressive Primary or Recurrent Glioblastoma Multiforme. | OV: H-1PV | Completed | I/II | Glioblastoma Multiforme | - |
| NCT02428036 | A Phase I Study of Repeated Intratumoral Administration of TBI-1401(HF10), a Replication Competent HSV-1 Oncolytic Virus, in Patients With Solid Tumors With Superficial Lesions | OV: HF10 | Completed | I | Solid Tumor | - |
| NCT01017185 | Phase I Trial of Intratumoral Administration of HF10, A Replication Competent Herpes Simplex Virus Type 1, in Patients With Refractory Head and Neck Cancer or Solid Tumors With Cutaneous and/or Superficial Lesions | OV: HF10 | Completed | I | Refractory Head and Neck Cancer，Squamous Cell Carcinoma, Skin，Carcinoma of the Breast，Malignant Melanoma | - |
| NCT03252808 | Phase I Study of Combination With TBI-1401(HF10), a Replication-competent HSV-1 Oncolytic Virus, and Chemotherapy in Patients With Stage III or IV Unresectable Pancreatic Cancer. | OV: HF10;  Other: Gemcitabine, Nab-paclitaxel, TS-1 | Active, not recruiting | I | Pancreatic Cancer | - |
| NCT03153085 | A Phase II Study of Combination Treatment With TBI-1401(HF10), a Replication-competent HSV-1 Oncolytic Virus, and Ipilimumab in Japanese Patients With Stage IIIB, IIIC, or IV Unresectable or Metastatic Malignant Melanoma | OV: HF10;  Other: Ipilimumab | Completed | II | Melanoma | -，CTLA4 |
| NCT02272855 | A Phase II Study of Combination Treatment With HF10, a Replication-competent HSV-1 Oncolytic Virus, and Ipilimumab in Patients With Stage IIIB, Stage IIIC, or Stage IV Unresectable or Metastatic Malignant Melanoma | OV: HF10;  Other: Ipilimumab | Completed | II | Melanoma | -，CTLA4 |
| NCT03259425 | Phase II Neoadjuvant Trial of Nivolumab in Combination With HF10 Oncolytic Viral Therapy in Resectable Stage IIIB, IIIC, IVM1a Melanoma (Neo-NivoHF10) | OV: HF10;  Other: Nivolumab | Terminated | II | Melanoma | PD-1，- |
| NCT00931931 | A Phase I Dose Escalation Study of Intratumoral or Intravenous Herpes Simplex Virus-1 Mutant HSV1716 in Patients With Refractory Non-Central Nervous System (Non-CNS) Solid Tumors | OV: HSV1716 | Completed | I | Rhabdomyosarcoma，Osteosarcoma，Ewing Sarcoma，Soft Tissue Sarcoma，Neuroblastoma，Wilms Tumor，Malignant Peripheral Nerve Sheath Tumor，Clival Chordoma，Non-CNS Solid Tumors | - |
| NCT01721018 | A Phase I/IIa Study of the Safety, Tolerability and Biological Effect of Single and Repeat Administration of the Selectively Replication-competent Herpes Simplex Virus HSV1716 Into the Tumor-bearing Pleural Cavity (Intrapleural) in Patients With Inoperable Malignant Pleural Mesothelioma. | OV: HSV1716 | Completed | I/II | Malignant Pleural Mesothelioma | - |
| NCT02031965 | A Phase I Study of Intratumoral/Peritumoral Herpes Simplex Virus-1 Mutant HSV1716 in Patients With Refractory or Recurrent High Grade Gliomas (HGG) | OV: HSV-1716;  Other: Dexamethasone | Terminated | I | Recurrent Childhood Anaplastic Astrocytoma，Recurrent Childhood Anaplastic Oligoastrocytoma，Recurrent Childhood Anaplastic Oligodendroglioma，Recurrent Childhood Giant Cell Glioblastoma，Recurrent Childhood Glioblastoma，Recurrent Childhood Gliomatosis Cerebri，Recurrent Childhood Gliosarcoma | - |
| NCT01864759 | Phase I Clinical Trial of Endovenous Administration of Conditionally Replicative Adenovirus ICOVIR-5 in Patients With Locally Advanced or Metastatic Melanoma | OV: ICOVIR-5 | Completed | I | Locally Advanced or Metastatic Melanoma | - |
| NCT00116363 | Phase II Study Examining the Biological Efficacy of Intratumoral INGN 241 (Ad-mda7) Administration in Patients With In Transit Melanoma | OV: INGN 241 | Unknown | II | Melanoma，Neoplasm Metastasis | IL24 |
| NCT00783588 | A Phase 1B Extension Trial to Allow Repeat Dosing of Autologous CLL B Cells Transduced to Express Chimeric CD154 (ISF35) in Subjects Previously Treated in MDACC Protocol 2004-0914 | OV: ISF35 | Completed | I | Chronic Lymphocytic Leukemia | CD40L |
| NCT00779883 | A Phase I Trial of Autologous CLL B Cells Transduced to Express Chimeric CD154 (ISF35) | OV: ISF35 | Completed | I | Chronic Lymphocytic Leukemia | CD40L |
| NCT00772486 | A Phase 1b Study of Repeated Doses of Autologous CLL B Cells Transduced to Express Chimeric CD154 (ISF35) in Combination With Fludarabine, Cyclophosphamide and Rituximab (FCR) in Subjects With Chronic Lymphocytic Leukemia (CLL) | OV: ISF35 | Completed | I | Chronic Lymphocytic Leukemia | CD40L |
| NCT00942409 | A Phase II Study of Repeat Intranodal Injections of Adenovirus-CD154 (Ad-ISF35) in Patients With Non-Hodgkin's Lymphoma (Follicular, Diffuse Large Cell, Mantle Cell and Small Lymphocytic Lymphoma/Chronic Lymphocytic Leukemia) | OV: ISF35 | Terminated | II | Non-Hodgkin's Lymphoma，Follicular Lymphoma，Diffuse Large Cell Lymphoma，Mantle Cell Lymphoma，Small Lymphocytic Lymphoma，Chronic Lymphocytic Leukemia | CD40L |
| NCT00849524 | A Phase II Study of Repeat Intranodal Injections of Adenovirus-CD 154 (Ad-ISF35) in Patients With Chronic Lymphocytic Leukemia/ Small Lymphocytic Lymphoma | OV: ISF35 | Terminated | II | Chronic Lymphocytic Leukemia，Small Lymphocytic Lymphoma | CD40L |
| NCT05427487 | A Phase 1 Open-label, Non-randomized, Multi-cohort Clinical Study of Intratumoral IVX037 in Patients With Advanced or Metastatic Solid Tumours | OV: IVX037 | Not yet recruiting | I | Colorectal Cancer，Gastroesophageal Cancer，Ovarian Cancer | - |
| NCT01469611 | A Phase 1b Dose Escalation Study of JX-594 (Thymidine Kinase-Inactivated Vaccinia Virus Plus GM-CSF) Administered by Biweekly (Every Two Weeks) Intravenous Infusion in Patients With Metastatic, Refractory Colorectal Carcinoma | OV: JX-594 | Completed | I | Colorectal Carcinoma | CSF2R |
| NCT01380600 | A Phase 1b Dose Escalation Study of JX-594 (Thymidine Kinase-Inactivated Vaccinia Virus Plus GM-CSF) Administered by Biweekly (Every Two Weeks) Intravenous Infusion in Patients With Metastatic, Refractory Colorectal Carcinoma | OV: JX-594 | Completed | I | Carcinoma, Colorectal | CSF2R |
| NCT01169584 | A Phase I, Open-Label, Dose Escalation Study of JX-594 (Vaccinia GM-CSF/Thymidine Kinase-Deactivated Virus) Administered by Intratumoral Injection in Pediatric Patients With Unresectable Refractory Solid Tumors. | OV: JX-594 | Completed | I | Neuroblastoma，Rhabdomyosarcoma，Lymphoma，Wilm's Tumor，Ewing's Sarcoma | CSF2R |
| NCT00629759 | Phase 1 Clinical Study for Evaluating the Safety and Efficacy of a Transdermal Injection of JX-594 (Thymidine Kinase (-)/GM-CSF(+) Vaccinia Virus) Within the Tumor of Patients With Hepatic Carcinoma | OV: JX-594 | Completed | I | Neoplasms | CSF2R |
| NCT00625456 | A Phase I Dose Escalation Study of JX-594 (Thymidine Kinase-deleted Vaccinia Virus Plus GM-CSF) Administered by Intravenous Infusion in Patients With Refractory Solid Tumors | OV: JX-594 | Completed | I | Melanoma，Lung Cancer，Renal Cell Carcinoma，Squamous Cell Carcinoma of the Head and Neck | CSF2R |
| NCT00429312 | A Phase I/II, Open-Label Study of JX-594 (Thymidine Kinase-deleted Vaccinia Virus Plus GM-CSF) Administered by Intratumoral Injection in Patients With Unresectable Stage 3 or Stage 4 Malignant Melanoma | OV: JX-594 | Completed | I/II | Melanoma | CSF2R，KLK3 |
| NCT02017678 | A Single-arm, Open-label, Phase 2 Study of JX-594 (Thymidine Kinase-Deactivated Vaccinia Virus Plus GM-CSF) Administered by 5 Weekly Intravenous (IV) Infusions in Patients With Peritoneal Carcinomatosis of Ovarian Cancer Origin | OV: JX-594 | Terminated | II | Ovarian Cancer | CSF2R |
| NCT01636284 | A Single-Arm, Open-Label Phase 2 Study of JX 594 (Thymidine Kinase-Deactivated Vaccinia Virus Plus GM-CSF) Administered by Weekly Intravenous (IV) Infusions in Sorafenib-naïve Patients With Advanced Hepatocellular Carcinoma (HCC) | OV: JX-594 | Completed | II | Hepatocellular Carinoma | CSF2R |
| NCT01387555 | A Phase 2b Randomized Trial of JX-594 (Vaccinia GM-CSF / TK-deactivated Virus) Plus Best Supportive Care Versus Best Supportive Care in Patients With Advanced Hepatocellular Carcinoma Who Have Failed Sorafenib Treatment | OV: JX-594 | Completed | II | Hepatocellular Carcinoma | CSF2R |
| NCT00554372 | A Phase II-a, Open-Label, Randomized Study of JX-594 (Thymidine Kinase-deleted Vaccinia Virus Plus GM-CSF) Administered by Intratumoral Injection in Patients With Unresectable Primary Hepatocellular Carcinoma | OV: JX-594 | Completed | II | Hepatocellular Carcinoma | CSF2R |
| NCT01329809 | A Phase IIa Study of Neoadjuvant JX-594 (Thymidine Kinase-Deactivated Vaccinia Virus Plus GM-CSF) Administered by Intravenous Infusion or Intratumoral Injection Followed by Surgical Resection in Patients With Metastatic Colorectal Tumors Within the Liver | OV: JX-594 | Terminated | II | Colorectal Carcinoma | CSF2R |
| NCT02630368 | A Phase I/II Study of Metronomic Cyclophosphamide and Oncolytic Poxvirus JX-594 in Patients With Advanced Hormone-receptor Positive and Triple Negative Breast Cancer and Advanced Soft Tissue Sarcoma (METROmaJX) | OV: JX-594;  Other: Cyclophosphamide | Recruiting | I/II | Solid Tumors，Soft-tissue Sarcoma，Breast Cancer | PD-L1，CSF2R |
| NCT01394939 | A Phase 1/2a Dose-escalation Study of JX 594 Administered by Multiple Intravenous (IV) Infusions Alone and in Combination With Irinotecan in Patients With Metastatic, Refractory Colorectal Carcinoma. | OV: JX-594;  Other: Irinotecan | Completed | I/II | Colorectal Carcinoma | CSF2R，TOP1 |
| NCT01171651 | A Phase 2 Open-Label Pilot Safety Study of JX-594 (Vaccinia GM-CSF/Thymidine Kinase-Deactivated Virus) Administered by IV Infusion Followed by Intratumoral Injection Prior to Standard Sorafenib Treatment in Patients With Unresectable Primary Hepatocellular Carcinoma | OV: JX-594;  Other: Sorafenib | Completed | II | Hepatocellular Carcinoma | CSF2R |
| NCT04577807 | Lerapolturev (Formerly Known as PVSRIPO) With or Without Immune Checkpoint Blockade in Advanced PD-1 Refractory Melanoma | OV: Lerapolturev; Other: Anti-PD-1 Checkpoint Inhibitor | Recruiting | II | Melanoma | PVR |
| NCT04479241 | A Phase 2, Open-label, Single-arm Study Evaluating the Efficacy, Safety and Tolerability of Lerapolturev (PVSRIPO) and the Immune Checkpoint Inhibitor Pembrolizumab in the Treatment of Patients With Recurrent Glioblastoma | OV: Lerapolturev; Other: Pembrolizumab | Active, not recruiting | II | Glioblastoma，Recurrent Glioblastoma，Supratentorial Glioblastoma，Brain Tumor | PVR，PD-1 |
| NCT04690699 | LUMINOS-103: A Basket Trial Evaluating the Safety and Efficacy of Lerapolturev (PVSRIPO) and Lerapolturev in Combination With Anti-PD-1/L1 Checkpoint Inhibitors in Patients With Advanced Solid Tumors | OV: Lerapolturev; Other:5% DDM | Recruiting | I/II | Solid Tumor | PVR |
| NCT03225989 | Phase I/II Trial Investigating an Immunostimulatory Oncolytic Adenovirus for Cancer | OV: LOAd703 | Recruiting | I/II | Pancreatic Adenocarcinoma，Ovarian Cancer，Biliary Carcinoma，Colorectal Cancer | CD46、CD40、4-1BB |
| NCT04123470 | A Phase I/II Trial Investigating LOAd703 in Combination With Atezolizumab in Malignant Melanoma | OV: LOAd703;  Other: Atezolizumab | Recruiting | I/II | Malignant Melanoma | CD46、CD40、4-1BB，PD-L1 |
| NCT02705196 | Phase I/IIa Trial Evaluating Safety of LOAd703, an Armed Oncolytic Adenovirus for Pancreatic Cancer | OV: LOAd703;  Other: Gemcitabine, Nab-paclitaxel, Atezolizumab | Recruiting | I/II | Pancreatic Cancer | CD46、CD40、4-1BB，RNR，Tubulin，PD-L1 |
| NCT03555149 | A Phase Ib/II, Open-Label, Multicenter, Randomized Umbrella Study Evaluating the Efficacy and Safety of Multiple Immunotherapy-Based Treatment Combinations in Patients With Metastatic Colorectal Cancer (Morpheus-CRC) | OV: LOAd703;  Other: Regorafenib, Atezolizumab, Imprime PGG, Bevacizumab, Isatuximab, Selicrelumab, Idasanutlin, AB928 | Active, not recruiting | I/II | Colorectal Cancer | PD-L1，-，VEGF，CD38，CD40，MDM2，ADORA2A、ADORA2B，CD46、CD40、4-1BB |
| NCT02062827 | A Phase 1 Study of M032 (NSC 733972), a Genetically Engineered HSV-1 Expressing IL-12, in Patients With Recurrent/Progressive Glioblastoma Multiforme, Anaplastic Astrocytoma, or Gliosarcoma | OV: M032 | Active, not recruiting | I | Recurrent Glioblastoma Multiforme，Progressive Glioblastoma Multiforme，Anaplastic Astrocytoma or Gliosarcoma | - |
| NCT05084430 | A Phase I/II Study of Pembrolizumab and M032 (NSC 733972), a Genetically Engineered HSV-1 Expressing IL-12, in Patients With Recurrent/Progressive Glioblastoma Multiforme, Anaplastic Astrocytoma, or Gliosarcoma | OV: M032;  Other: Prembrolizumab | Active, not recruiting | I/II | Glioblastoma Multiforme，Anaplastic Astrocytoma，Gliosarcoma | PD-1，- |
| NCT04665362 | A Single-arm Study to Evaluate the Safety and Efficacy of Recombinant Oncolytic Virus M1 (M1-c6v1) Combined With Anti-PD-1 Antibody SHR-1210 and Apatinib for Treatment of Patients With Advanced / Metastatic Hepatocellular Carcinoma | OV: M1;  Other: anti PD-1 antibody, Apatinib | Not yet recruiting | I | Advanced/Metastatic Hepatocellular Carcinoma | -，PD-1，VEGFR-2 |
| NCT03889275 | An Open-label Phase 1 Study to Assess the Safety, Tolerability, Pharmacokinetics, Pharmacodynamics and Preliminary Efficacy of MEDI5395 in Combination With Durvalumab in Subjects With Select Advanced Solid Tumors. | OV: MEDI5395;  Other: Durvalumab | Active, not recruiting | I | Solid Tumor | CSFR，PD-L1 |
| NCT04613492 | An Open-label, Phase 1 Study to Assess the Safety, Tolerability, Pharmacokinetics, Pharmacodynamics and Preliminary Efficacy of MEDI9253, a Recombinant Newcastle Disease Virus Encoding Interleukin-12, in Combination With Durvalumab in Participants With Select Advanced/Metastatic Solid Tumors | OV: MEDI9253;  Other: Durvalumab | Recruiting | I | Solid Tumor | IL12R，PD-L1 |
| NCT05076760 | Phase I Study of MEM-288 Oncolytic Virus in Solid Tumors Including Non-Small Cell Lung Cancer (NSCLC) | OV: MEM-288 | Recruiting | I | Solid Tumor，Advanced Cancer，Metastatic Cancer，Non Small Cell Lung Cancer，Cutaneous Squamous Cell Carcinoma，Merkel Cell Carcinoma，Melanoma，Pancreatic Cancer，Triple Negative Breast Cancer，Head and Neck Cancer | CD40、IFNAR |
| NCT04521764 | Phase I Trial of Intratumoral Administration of a Measles Virus Derivative Expressing the Helicobacter Pylori Neutrophil-Activating Protein (NAP) (MV-s-NAP) in Patients With Metastatic Breast Cancer | OV: MV-s-NAP | Recruiting | I | Anatomic Stage IV Breast Cancer ，Invasive Breast Carcinoma，Metastatic Breast Adenocarcinoma，Prognostic Stage IV Breast Cancer，Recurrent Breast Carcinoma | - |
| NCT03852511 | A Multicentre, Open Label, Non-randomised First in Human Study of NG-350A (Monotherapy), and NG-350A With a Check Point Inhibitor in Patients With Metastatic or Advanced Epithelial Tumours | OV: NG-350A | Completed | I | Metastatic Cancer，Epithelial Tumor | CD40 |
| NCT05165433 | A Multicentre, Open-label, Non-randomized, Phase 1a/1b Study of NG-350A, a Tumour-selective Anti-CD40-expressing Adenoviral Vector, in Combination With Pembrolizumab in Patients With Metastatic or Advanced Epithelial Tumours | OV: NG-350A;  Other: Pembrolizumab | Recruiting | I | Epithelial Tumor，Metastatic Cancer | CD40 |
| NCT04053283 | A Multicentre, Open-label, Non-randomised First in Human Study of NG-641, an Oncolytic Transgene Expressing Adenoviral Vector, in Patients With Metastatic or Advanced Epithelial Tumours (STAR) | OV: NG-641 | Recruiting | I | Metastatic Cancer，Epithelial Tumor | FAP-α、CD3、CXCR3、IFNAR |
| NCT05043714 | A Multicentre, Open-label, Non-randomized, Phase 1a/1b Study of NG-641, a Tumour-selective and Transgene-expressing Adenoviral Vector, in Combination With Nivolumab in Patients With Metastatic or Advanced Epithelial Tumours (NEBULA) | OV: NG-641;  Other: Nivolumab | Recruiting | I | Metastatic Cancer，Epithelial Tumor | FAP-α、CD3、CXCR3、IFNAR |
| NCT04830592 | A Multicentre, Open-label, Dose-escalating, Phase Ib, Study of Intravenous Dosing of NG-641, as Monotherapy or in Combination With Pembrolizumab in Patients With Surgically Resectable Squamous Cell Carcinoma of the Head and Neck | OV: NG-641;  Other: Pembrolizumab | Recruiting | I | Squamous Cell Carcinoma of the Head and Neck | FAP-α、CD3、CXCR3、IFNAR |
| NCT01048892 | A Phase 1 Dose Escalation Study of Seneca Valley Virus (NTX-010), A Replication-Competent Picornavirus, in Relapsed/Refractory Pediatric Patients With Neuroblastoma, Rhabdomyosarcoma, or Rare Tumors With Neuroendocrine Features | OV: NTX-010;  Other: Cyclophosphamide | Completed | I | Adrenocortical Carcinoma，Gastrointestinal Carcinoid Tumor，Kidney Cancer，Neuroblastoma，Retinoblastoma，Sarcoma | ANTXR1，- |
| NCT00012155 | A Phase I, Open-Label, Dose-Escalating Study Of The Safety, Tolerability, And Anti-Tumor Activity Of A Single Intrahepatic Arterial Injection Of Genetically Engineered Herpes Simplex Virus, NV1020, In Subjects With Adenocarcinoma Of The Colon With Metastasis To The Liver | OV: NV1020 | Completed | I | Colorectal Cancer，Metastatic Cancer | - |
| NCT00149396 | A Phase I/II, Open-label Study to Evaluate the Safety and Anti-tumor Effects of NV1020 Administered Repeatedly Via Hepatic Artery Infusion Prior to Second-line Chemotherapy, in Patients With Colorectal Adenocarcinoma Metastatic to the Liver | OV: NV1020 | Completed | I/II | Colorectal Cancer，Liver Neoplasms | - |
| NCT02293850 | A Phase I Study to Evaluate the Safety and Efficacy of Telomelysin (OBP-301) in Patients With Hepatocellular Carcinoma | OV: OBP-301 | Unknown | I | Carcinoma, Hepatocellular | TERT |
| NCT03190824 | Open-label, Multi-center Phase IIa Study to Evaluate the Efficacy, Safety, and Immunological Response of OBP-301, Telomerase Specific Replication-competent Oncolytic Adenovirus in Patients With Unresectable Metastatic Melanoma | OV: OBP-301 | Unknown | II | Melanoma | TERT |
| NCT03172819 | An Open Label Phase I Study to Evaluate the Safety and Efficacy of OBP-301 With Pembrolizumab in Patients With Advanced Solid Tumors | OV: OBP-301;  Other: Pembrolizumab | Completed | I | Advanced Solid Tumor | TERT，PD-1 |
| NCT04685499 | Phase 2 Study of OBP-301 (Telomelysin ™) in Combination With Pembrolizumab and Stereotactic Body Irradiation in Patients With Head and Neck Squamous Cell Carcinoma With Inoperable, Recurrent or Progressive Disease | OV: OBP-301;  Other: Pembrolizumab | Terminated | II | Head and Neck Squamous Cell Carcinoma With Inoperable Recurrent or Progressive Disease | TERT，PD-1 |
| NCT03213054 | A Phase 1 Study to Evaluate the Safety of OBP-301, Telomelysin in Combination With Radiation Therapy in Patients With Esophageal Cancer Not Applicable for Standard Therapy | OV: OBP-301;  Other: Radiation | Unknown | I | Esophageal Cancer | TERT |
| NCT05232136 | Oncolytic Virus (OH2) Adjuvant Therapy After Transurethral Resection of Bladder Tumor in Non-Muscle-Invasive Bladder Cancer Who Have Failed First-line Prophylactic Intravesical Instillation Therapy: a Phase Ⅰb/Ⅱ Clinical Trial | OV: OH2 | Recruiting | I/II | Non-muscle-invasive Bladder Cancer | CSF2R |
| NCT05235074 | A Clinical Study of Oncolytic Virus (OH2) Injection in the Treatment of Patients Undergoing Surgery After Recurrence of Central Nervous System Tumors | OV: OH2 | Recruiting | I/II | Central Nervous System Tumors | CSF2R |
| NCT04637698 | Phase Ib/II Study of OH2 Injection, an Oncolytic Type 2 Herpes Simplex Virus Expressing Granulocyte Macrophage Colony-Stimulating Factor, in Pancreatic Cancer | OV: OH2 | Recruiting | I/II | Pancreatic Cancer | CSF2R |
| NCT05248789 | Efficacy and Safety Study of Oncolytic Virus (OH2) Intratumoral Injection in Locally Advanced or Metastatic Bladder Cancer a Phase Ⅱ Clinical Trial | OV: OH2 | Recruiting | II | Advanced Bladder Carcinoma | CSF2R |
| NCT03866525 | Phase I/II Study of OH2 Injection, an Oncolytic Type 2 Herpes Simplex Virus Expressing Granulocyte Macrophage Colony-Stimulating Factor, in Malignant Solid Tumors | OV: OH2 injection;  Other: HX008 | Recruiting | I/II | Solid Tumor | CSF2R |
| NCT04616443 | Phase Ib Study of the Combination Use of Recombinant Human GM-CSF Type II Herpes Simplex Virus (OH2) Injection (Vero Cells) and HX008 Injection in the Treatment of Melanoma | OV: OH2;  Other: HX008 | Recruiting | I/II | Melanoma | CSF2R，PD-1 |
| NCT04386967 | Open and Incremental Phase I Clinical Trial of Recombinant Human GM-CSF Type II Herpes Simplex Virus (OH2) Injection (Vero Cells) in the Treatment of Advanced Solid Tumors | OV: OH2;  Other: Pembrolizumab | Recruiting | I/II | Solid Tumor | CSF2R |
| NCT05281471 | A Randomized Phase 3 Study Assessing the Efficacy and Safety of Olvi-Vec Followed by Platinum-doublet Chemotherapy and Bevacizumab Compared With Platinum-doublet Chemotherapy and Bevacizumab in Women With Platinum-Resistant/Refractory Ovarian Cancer (OnPrime, GOG-3076) | OV: Olvi-Vec;  Other: Non-platinum chemotherapy: Physician's Choice of gemcitabine, taxane (paclitaxel, docetaxel or nab-paclitaxel) or pegylated liposomal doxorubicin, Bevacizumab (or biosimilar) | Recruiting | III | Platinum-resistant Ovarian Cancer，Platinum-refractory Ovarian Cancer，Fallopian Tube Cancer，Primary Peritoneal Cancer，High-grade Serous Ovarian Cancer，Endometrioid Ovarian Cancer，Ovarian Clear Cell Carcinoma | - |
| NCT03072134 | Neural Stem Cell Oncolytic Adenoviral Virotherapy of Newly Diagnosed Malignant Glioma | OV: Oncolytic adenovirus | Completed | I | Glioma，Anaplastic Astrocytoma，Anaplastic Oligodendroglioma，Anaplastic Oligoastrocytoma，Glioblastoma Multiforme，Astrocytoma, Grade III，Astrocytoma, Grade IV，Brain Cancer | - |
| NCT01598129 | Exploratory Open Label Study of GM-CSF Coding Oncolytic Adenovirus CGTG-102, With Low Dose Cyclophosphamide in Patients With Refractory Injectable Solid Tumours | OV: ONCOS-102 | Completed | I | Solid Tumor | CSF2R、DSG2、CD46 |
| NCT05561491 | An Open-Label, Two-Part, Dose-Exploration and Multiple Expansion, Phase 2 Study of ONCOS-102 in Combination With Novel Immune-Targeted Anti-Cancer Agents in Patients With Unresectable or Metastatic Cutaneous Melanoma Resistant to Anti-PD-(L)1 Treatment | OV: ONCOS-102;  Other: Balstilimab | Not yet recruiting | II | Melanoma | CSF2R、DSG2、CD46，PD-1 |
| NCT03003676 | A Pilot Study of Sequential ONCOS-102, an Engineered Oncolytic Adenovirus Expressing GMCSF, and Pembrolizumab in Patients With Advanced or Unresectable Melanoma Progressing After PD1 Blockade | OV: ONCOS-102;  Other: Cyclophosphamide, Pembrolizumab | Completed | I | Advanced or Unresectable Melanoma Progressing After PD1 Blockade | CSF2R、DSG2、CD46 |
| NCT02963831 | A Phase 1/2 Dose Escalation Study With Expansion Cohorts to Investigate the Safety, Biologic and Anti-tumor Activity of ONCOS-102 in Combination With Durvalumab in Subjects With Advanced Peritoneal Malignancies | OV: ONCOS-102;  Other: Durvalumab | Completed | I/II | Colorectal Cancer，Platinum-resistant Ovarian Cancer，Appendiceal Cancer | CSF2R、DSG2、CD46 |
| NCT04348916 | A Phase 1, Open-Label, Multicenter, Dose Escalation and Expansion Study of ONCR-177, an Oncolytic Herpes Simplex Virus for Intratumoral Injection, Alone and in Combination With PD-1 Blockade in Adult Subjects With Advanced and/or Refractory Cutaneous, Subcutaneous or Metastatic Nodal Solid Tumors or With Liver Metastases of Solid Tumors | OV: ONCR-177;  Other: Pembrolizumab | Recruiting | I | Solid Tumor | FLT3、PD-1、CTLA4、IL12R、CCL4，PD-1 |
| NCT00006106 | Phase I Study of Intraarterial ONYX-015 in Combination With Intravenous Cisplatin and Fluorouracil in Patients With Advanced Squamous Cell Cancer of the Head and Neck | OV: ONYX-015;  Other: Cisplatin, Fluorouracil | Terminated | I | Lip and Oral Cavity Cancer，Head and Neck Cancer，Oropharyngeal Cancer | p53，-，TYMS |
| NCT04097002 | A Phase I/IIa Study Evaluating the Safety and Tolerability of Intratumoral Administration of ORCA-010 in Treatment-Naïve Patients With Localized Prostate Cancer. | OV: ORCA-010 | Recruiting | I/II | Adenocarcinoma of the Prostate | - |
| NCT03048253 | Recombinant Human GM-CSF Herpes Simplex Virus Injection (OrienX010) Standard Injection in Tumor Treatment Scheme Failed M1c Ⅳ Period Malignant Melanoma Spread to the Liver Open I-c Phase of Clinical Trial | OV: OrienX010 | Unknown | I | Melanoma | CSF2 |
| NCT04200040 | A Phase II Clinical Study to Evaluate the Safety and Efficacy of OrienX010 in Previously Untreated With Dacarbazine Patients in Unresectable Stage IIIb/IIIc or Stage IV(Mla/Mlb) Malignant Melanoma | OV: OrienX010;  Other: Dacarbazine | Recruiting | II | Melanoma (Skin) | CSF2 |
| NCT04206358 | An Open Phase Ib Clinical Study to Evaluate OrienX010 in Combination With JS001 in the Treatment of Stage IV (M1c) Liver Metastasis From Melanoma | OV: OrienX010;  Other: JS001 | Recruiting | I | Melanoma | CSF2，PD-1 |
| NCT04197882 | An Open-Label, Phase Ib Clinical Study to Evaluate OrienX010 in Combination With Toripalimab as Neoadjuvant Treatment in the Patients With Complete Resectable Stage III and Stage IV (M1a) Melanoma | OV: OrienX010;  Other: Toripalimab | Active, not recruiting | I | Melanoma | CSF2，PD-1 |
| NCT04787003 | A Single-arm, Open-label Clinical Study to Evaluate the Safety, Tolerability and Preliminary Efficacy of Oncolytic Virus (OVV-01) Injection Combined With or Without Immune Checkpoint Inhibitors in the Treatment of Patients With Advanced Solid Tumors | OV: OVV-01 | Recruiting | I | Neoplasms | - |
| NCT00528684 | A Phase I/II Clinical Trial to Evaluate Dose Limiting Toxicity and Efficacy of Intralesional Administration of REOLYSIN® for the Treatment of Patients With Histologically Confirmed Recurrent Malignant Gliomas | OV: Pelareorep | Completed | I | Malignant Glioma | - |
| NCT05519059 | A Multicenter, Single-Arm, Open-Label Phase I Clinical Study of Pelareorep With Paclitaxel in Advanced or Metastatic Breast Cancer | OV: Pelareorep | Active, not recruiting | I | Advanced or Metastatic Breast Cancer | - |
| NCT00503295 | A Phase 2 Study of Intravenous REOLYSIN® (Wild-Type Reovirus) in the Treatment of Patients With Bone and Soft Tissue Sarcomas Metastatic to the Lung | OV: Pelareorep | Completed | II | Osteosarcoma，Ewing Sarcoma Family Tumors，Malignant Fibrous Histiocytoma，Sarcoma, Synovial，Fibrosarcoma，Leiomyosarcoma | - |
| NCT01199263 | A Randomized Phase II Evaluation of Weekly Paclitaxel (NSC# 673089) Versus Weekly Paclitaxel With Oncolytic Reovirus (Reolysin NSC # 729968) in the Treatment of Recurrent or Persistent Ovarian, Fallopian Tube or Primary Peritoneal Cancer | OV: Pelareorep,  Other: Paclitaxel | Completed | II | Recurrent Fallopian Tube Carcinoma，Recurrent Ovarian Carcinoma，Recurrent Primary Peritoneal Carcinoma | - |
| NCT04215146 | A Study to Assess Overall Response Rate by Inducing an Inflammatory Phenotype in Metastatic BReast cAnCEr With the Oncolytic Reovirus PeLareorEp in CombinaTion With Anti-PD-L1 Avelumab and Paclitaxel - BRACELET-1 Study | OV: Pelareorep;  Other: Avelumab, Paclitaxel | Active, not recruiting | II | Breast Cancer Metastatic | PD-L1，- |
| NCT05514990 | A Phase 1b/2 Study of Standard Doses of Bortezomib and Pembrolizumab ± Reovirus (Pelareorep) Combination Therapy in Patients With Relapsed Multiple Myeloma (AMBUSH Study) | OV: Pelareorep;  Other: Bortezomib, Dexamethasone, Pembrolizumab | Not yet recruiting | I/II | Recurrent Plasma Cell Myeloma，Refractory Plasma Cell Myeloma | 26S Proteasome，ANXA1、NR3C1、NR1I2、NOS2、NR0B1，-，PD-1 |
| NCT00984464 | A Phase 2 Study of Intravenous Administration of REOLYSIN (Reovirus Type 3 Dearing) in Combination With Paclitaxel and Carboplatin in Patients With Metastatic Melanoma | OV: Pelareorep;  Other: Carboplatin, Paclitaxel | Completed | II | Metastatic Melanoma | - |
| NCT00861627 | Phase 2 Study of Intravenous Administration of Reovirus Serotype 3 - Dearing Strain (REOLYSIN®) in Combination With Paclitaxel and Carboplatin in Patients With Metastatic or Recurrent Non-Small Cell Lung Cancer Who Have KRAS or EGFR Activated Tumors | OV: Pelareorep;  Other: Carboplatin, Paclitaxel | Completed | II | Non-small Cell Lung Cancer | - |
| NCT00753038 | Phase 2 Study of Intravenous Administration of a Wild-Type Reovirus (REOLYSIN®) in Combination With Paclitaxel and Carboplatin in Patients With Platinum-Refractory Metastatic and/or Recurrent Squamous Cell Carcinoma of the Head and Neck. | OV: Pelareorep;  Other: Carboplatin, Paclitaxel | Completed | II | Head and Neck Cancer | - |
| NCT01166542 | Randomized, Double-blind, Multicenter Two-Stage Adaptive Phase 3 Study of Intravenous Administration of REOLYSIN (Reovirus Type 3 Dearing) in Combination With Paclitaxel and Carboplatin Versus the Chemotherapy Alone in Patients With Metastatic or Recurrent Squamous Cell Carcinoma of the Head and Neck Who Have Progressed on or After Prior Platinum-Based Chemotherapy | OV: Pelareorep;  Other: Carboplatin, Paclitaxel, Placebo | Recruiting | III | Squamous Cell Carcinoma，Head and Neck Cancer | - |
| NCT02101944 | Pilot Trial Evaluating Viral Protein Production From the Combination of Reolysin and Carfilzomib in Multiple Myeloma | OV: Pelareorep;  Other: Carfilzomib, Dexamethasone | Active, not recruiting | I | Anemia，Recurrent Plasma Cell Myeloma，Refractory Plasma Cell Myeloma | 20S Proteasome，ANXA1、NR3C1、NR1I2、NOS2、NR0B1，- |
| NCT03605719 | PD1 Blockade and Oncolytic Virus in Relapsed Multiple Myeloma | OV: Pelareorep;  Other: Carfilzomib, Dexamethasone, Nivolumab | Active, not recruiting | I | Recurrent Plasma Cell Myeloma | PD-1，- |
| NCT02620423 | A Phase 1b Study of Pembrolizumab (KEYTRUDA®) in Combination With REOLYSIN® (Pelareorep) and Chemotherapy in Patients With Advanced Pancreatic Adenocarcinoma | OV: Pelareorep;  Other: Chemotherapy, Gemcitabine, Irinotecan, Leucovorin, 5-fluorouracil, Pembrolizumab | Completed | I | Pancreatic Adenocarcinoma | PD-1，- |
| NCT01619813 | A Randomized Phase II Study of Reolysin in Combination With Docetaxel and Prednisone or Docetaxel and Prednisone Alone in Patients With Metastatic Castration Resistant Prostate Cancer | OV: Pelareorep;  Other: Docetaxel, Prednisone | Completed | II | Prostate Cancer | - |
| NCT01622543 | A Randomized Phase II Study of Reolysin in Combination With FOLFOX6 and Bevacizumab or FOLFOX6 and Bevacizumab Alone in Patients With Metastatic Colorectal Cancer. | OV: Pelareorep;  Other: Folfox, Bevacizumab | Completed | II | Colorectal Cancer | VEGF，- |
| NCT00998322 | A Phase 2 Study of REOLYSIN in Combination With Gemcitabine for Patients With Advanced Pancreatic Adenocarcinoma | OV: Pelareorep;  Other: Gemcitabine | Completed | II | Metastatic Pancreatic Adenocarcinoma | - |
| NCT02723838 | A Phase 1b Study of Intratumoral REOLYSIN® in Combination With Gemcitabine and Cisplatin as Neoadjuvant Therapy in Muscle-invasive Transitional Cell Carcinoma of the Bladder | OV: Pelareorep;  Other: Gemcitabine, Cisplatin | Terminated | I | Muscle-invasive Transitional Cell Carcinoma of the Bladder | - |
| NCT03282188 | A Clinical Study to Evaluate the Biological Effects of Intravenous Wild-type Reovirus (Reolysin®), With of Without GM-CSF, in Advanced Melanoma | OV: Pelareorep; Other: GM-CSF | Terminated | I/II | Melanoma，Cancer of Skin | CSF2R，- |
| NCT01656538 | A Randomized Phase II Study of Reolysin For Patients Receiving Standard Weekly Paclitaxel Therapy as Therapy For Advanced/Metastatic Breast Cancer | OV: Pelareorep;  Other: Paclitaxel | Completed | II | Metastatic Breast Cancer | - |
| NCT00998192 | A Phase 2 Study of Intravenous Administration of REOLYSIN® (Reovirus Type 3 Dearing) in Combination With Paclitaxel and Carboplatin in Patients With Squamous Cell Carcinoma of the Lung | OV: Pelareorep;  Other: Paclitaxel, Carboplatin | Completed | II | Metastatic or Recurrent Squamous Cell Carcinoma of the Lung | - |
| NCT01708993 | A Randomized Phase II Study of Reolysin in Patients With Previously Treated Advanced or Metastatic, Non Small Cell Lung Cancer Receiving Standard Salvage Therapy. | OV: Pelareorep;  Other: Pemetrexed, Docetaxel | Completed | II | Non Small Cell Lung Cancer | - |
| NCT04445844 | IRENE Study: Phase 2 Study of INCMGA00012 and the Oncolytic Virus Pelareorep in Metastatic Triple Negative Breast Cancer | OV: Pelareorep;  Other: Retifanlimab | Recruiting | II | Anatomic Stage IV Breast Cancer AJCC v8，Locally Advanced Breast Carcinoma，Metastatic Triple-Negative Breast Carcinoma，Prognostic Stage IV Breast Cancer AJCC v8，Triple-negative Breast Cancer | -，PD-1 |
| NCT01274624 | A Multicenter Phase 1 Study of Intravenous Administration of REOLYSIN® (Reovirus Type 3 Dearing) in Combination With Irinotecan/Fluorouracil/Leucovorin (FOLFIRI) and Bevacizumab in FOLFIRI Naive Patients With KRAS Mutant Metastatic Colorectal Cancer | OV: Pelareorep;  Other: Irinotecan, Leucovorin, Fluorouracil, Bevacizumab | Completed | I | KRAS Mutant Metastatic Colorectal Cancer | - |
| NCT05492682 | A Study to Evaluate the Safety and Immune Activity of PeptiCRAd-1 in Combination With Pembrolizumab in Patients With Injectable Solid Tumors in Indications Known to Express NY-ESO-1 and MAGE-A3 | OV: PeptiCRAd-1; Other: Cyclophosphamide, Pembrolizumab | Not yet recruiting | I | Melanoma (Skin)，Triple-Negative Breast Cancer，Non-Small Cell Lung Cancer | - |
| NCT03071094 | A Phase I/IIa Trial to Evaluate the Safety and Efficacy of the Combination of the Oncolytic Immunotherapy Pexa-Vec With the PD-1 Receptor Blocking Antibody Nivolumab in the First-line Treatment of Advanced Hepatocellular Carcinoma (HCC) | OV: Pexa Vec;  Other: Nivolumab | Terminated | I/II | Hepatocellular Carcinoma | CSF2R，PD-1 |
| NCT02562755 | A Phase 3 Randomized, Open-Label Study Comparing Pexa Vec (Vaccinia GM CSF / Thymidine Kinase-Deactivated Virus) Followed by Sorafenib Versus Sorafenib in Patients With Advanced Hepatocellular Carcinoma (HCC) Without Prior Systemic Therapy | OV： Pexa Vec;  Other: Sorafenib | Recruiting | III | Hepatocellular Carcinoma | CSF2R |
| NCT03294083 | A Phase 1b/2a Dose-escalation and Safety/Efficacy Evaluation Study of Pexa-Vec (Thymidine Kinase-Deactivated Vaccinia Virus Plus GM-CSF) in Combination With Cemiplimab (REGN2810; Anti-PD-1) in Patients With Metastatic or Unresectable Renal Cell Carcinoma (RCC) | OV: Pexa-Vec;  Other: Cemiplimab | Active, not recruiting | I/II | Renal Cell Carcinoma | CSF2R，PD-1 |
| NCT02977156 | A Phase I Dose Escalation Trial Evaluating the Impact of an in Situ Immunization Strategy With Intra-Tumoral Injections of Pexa-Vec in Combination With Ipilimumab in Metastatic / Advanced Solid Tumors With Injectable Lesions. | OV: Pexa-Vec;  Other: Ipilimumab | Completed | I | Solid Tumor | CTLA4，CSF2R |
| NCT04849260 | Phase Ib/II, Open Label, Controlled Clinical Study of Pexa-Vec (Vaccinia GM CSF / Thymidine Kinase-Deactivated Virus) Combined With Recombinant Whole Human Anti-PD-L1 Monoclonal Antibody (ZKAB001) in Metastatic Melanoma After First-line Treatment Failure | OV: Pexa-Vec;  Other: ZKAB001 | Recruiting | I/II | Local Progression or Metastatic Melanoma With Failed First-line Treatment | CSF2R，PD-L1 |
| NCT05061537 | A PHASE 1, OPEN-LABEL, DOSE ESCALATION AND EXPANSION STUDY EVALUATING THE SAFETY AND PHARMACODYNAMICS OF PF-07263689, EITHER ALONE OR IN COMBINATION WITH AN ANTI-PD-1 ANTIBODY, IN PREVIOUSLY TREATED PARTICIPANTS WITH SELECTED LOCALLY ADVANCED OR METASTATIC SOLID TUMORS | OV: PF-07263689; Other: Sasanlimab | Active, not recruiting | I | Renal Cell Cancer，Melanoma，Non-Small-Cell Lung Cancer，Hepatocellular Cancer，Bladder Cancer，Sarcoma，Head and Neck Cancer，Colorectal Cancer，Ovarian Cancer，Squamous Cell Carcinoma | -，PD-1 |
| NCT00055705 | A Phase I Clinical Trial To Assess The Safety And Efficacy Of Intraperitoneal PV701 Administrations In Patients With Advanced Or Recurrent Malignancy Largely Confined To The Peritoneal Cavity | OV: PV701 | Completed | I | Solid Tumor | - |
| NCT00081211 | A Phase I Study Of PV701 In Patients With Head And Neck Squamous Cell Carcinoma | OV: PV701 | Terminated | I | Solid Tumor | - |
| NCT03712358 | A Phase I Trial of PVSRIPO for Patients With Unresectable Melanoma | OV: PVSRIPO | Completed | I | Melanoma | PVR |
| NCT03043391 | Phase Ib Study of Oncolytic Polio/Rhinovirus Recombinant Against Recurrent Malignant Glioma in Children | OV: PVSRIPO | Active, not recruiting | I | Malignant Glioma，Anaplastic Astrocytoma，Anaplastic Oligoastrocytoma，Anaplastic Oligodendroglioma，Glioblastoma，Gliosarcoma，Atypical Teratoid/Rhabdoid Tumor of Brain，Medulloblastoma，Ependymoma，Pleomorphic Xanthoastrocytoma of Brain，Embryonal Tumor of Brain | PVR |
| NCT02986178 | A Multicenter Phase 2 Study of Oncolytic Polio/Rhinovirus Recombinant (PVSRIPO) in Recurrent WHO Grade IV Malignant Glioma Patients | OV: PVSRIPO | Active, not recruiting | II | Malignant Glioma | PVR |
| NCT03973879 | A Phase 1b/2 Trial of PVSRIPO in Combination With Atezolizumab in Recurrent WHO Grade IV Malignant Glioma | OV: PVSRIPO;  Other: Atezolizumab | Terminated | I/II | Malignant Glioma | PD-L1，PVR |
| NCT04125719 | A Phase IB Trial of PVSRIPO in Combination With Nivolumab in Patients With Recurrent PD-1 Refractory Melanoma | OV: PVSRIPO;  Other: Nivolumab | Terminated | I | Melanoma | PVR，PD-1 |
| NCT02719015 | Phase I/II Dose Escalation and Cohort Expansion of Safety and Tolerability Study of Intratumoral rAd.CD40L (ISF35) in Combination of Systemic Pembrolizumab in Patients With Refractory Metastatic Melanoma | OV: rAd.CD40L;  Other: Pembrolizumab | Terminated | I/II | Melanoma | CD40L，PD-1 |
| NCT02435186 | Recombinant Adenoviral p53 Human Gene Combined With Chemotherapy in Treatment of Recurrent Ovarian Epithelial Cancer, Fallopian Tube Cancer, and Primary Peritoneal Cancer | OV: rAd-p53;  Other: Cisplatin, Paclitaxel | Unknown | II | Ovarian Epithelial Cancer，Fallopian Tube Cancer，Primary Peritoneal Cancer | p53 |
| NCT02429037 | Recombinant Adenoviral Human p53 Gene Combined With Radio- and Chemo-therapy in Treatment of Unresectable, Locally Advanced Head and Neck Cancer - a Open-labeled Randomized Phase 2 Study | OV: rAd-p53;  Other: Radiation, Cisplatin | Unknown | II | Advanced Head and Neck Cancer | p53 |
| NCT01574729 | Phase II Study of Surgery Combined With Recombinant Adenoviral Human p53 Gene Therapy in Treatment Advanced Non-small-cell Carcinoma | OV: rAd-p53;  Other: Surgery | Unknown | II | Non-small Cell Lung Cancer | p53 |
| NCT02561546 | A Phase II Study to Investigate Preliminary Efficacy Using p53 Gene Therapy for Treatment of Diabetes Concurrent With Hepatocellular Carcinoma | OV: rAd-p53;  Other: Trans-catheter embolization | Unknown | II | Hepatocellular Carcinoma | p53 |
| NCT02418988 | Multicenter, Open-labeled, Controlled Phase II Study: Trans-catheter Chemo-embolization Combined With rAd-p53 Gene Injection in Treatment of Advanced Hepatocellular Carcinoma | OV: rAd-p53;  Other: Trans-catheter embolization | Unknown | II | Hepatocellular Carcinoma | p53 |
| NCT01935453 | A Phase I Study of Recombinant hGM-CSF Herpes Simplex Virus | OV: Recombinant hGM-CSF Herpes Simplex Virus | Completed | I | Melanoma，Liver Cancer，Pancreatic Cancer，Lung Cancer | CSF2 |
| NCT04887025 | Exploratory Study of a Novel Oncolytic Vaccinia Virus Expressing Bispecific Antibody in the Treatment of Refractory/Relapsed B-cell Lymphoma | OV: RGV004 | Not yet recruiting | I | Relapsed or Refractory B-cell Lymphoma | CD19、CD3 |
| NCT05068453 | Safety and Efficacy Study of Oncolytic Virus（Intratumoral Injection）in Combination With HX-008（Intravenous Injection）and Radiotherapy for Liver Metastasis in Melanoma Patients With Liver Metastasis Who Lack or Become Refractory to Standard Treatment | OV: rHSV2hGM-CSF);  Other: anti-PD-1, Radiation | Not yet recruiting | I | Melanoma Stage IV | CSF2R，PD-1 |
| NCT05070221 | Safety and Efficacy Study of Oncolytic Virus（Intratumoral Injection）in Combination With HX-008（Intravenous Injection）and Axitinib in Melanoma Patients With Liver Metastasis Who Lack or Become Refractory to Standard Treatment | OV: rHSV2hGM-CSF);  Other: anti-PD-2, Axitinib | Not yet recruiting | I | Melanoma Stage IV | CSF2R，PD-1 |
| NCT04349436 | An Open-Label, Multicenter, Phase 1B/2 Study of RP1 in Solid Organ Transplant Recipients With Advanced Cutaneous Malignancies | OV: RP1 | Recruiting | I/II | Cutaneous Squamous Cell Carcinoma，Merkel Cell Carcinoma，Basal Cell Carcinoma，Melanoma | CSFR |
| NCT04050436 | A Randomized, Controlled, Open-Label, Phase 2 Study of Cemiplimab as a Single Agent and in Combination With RP1 in Patients With Advanced Cutaneous Squamous Cell Carcinoma | OV: RP1;  Other: Cemiplimab | Recruiting | II | Cutaneous Squamous Cell Carcinoma，Advanced Cutaneous Squamous Cell Carcinoma，Metastatic Cutaneous Squamous Cell Carcinoma | CSFR，PD-1 |
| NCT03767348 | An Open-Label, Multicenter, Phase 1/2 Study of RP1 as a Single Agent and in Combination With PD1 Blockade in Patients With Solid Tumors | OV: RP1;  Other: Nivolumab | Recruiting | II | Cancer，Melanoma (Skin)，Mismatch Repair Deficiency，Microsatellite Instability，Non-melanoma Skin Cancer，Cutaneous Melanoma，NSCLC | CSFR，PD-1 |
| NCT04336241 | An Open-Label, Multicenter, Phase 1 Study of RP2 as a Single Agent and in Combination With PD1 Blockade in Patients With Solid Tumors | OV: RP2;  Other: Nivolumab | Recruiting | I | Solid Tumor | CTLA4，PD-1 |
| NCT04735978 | An Open-Label, Multicenter, Phase 1 Study of RP3 as a Single Agent and in Combination With PD-1 Blockade in Patients With Solid Tumors | OV: RP3;  Other: Nivolumab | Recruiting | I | Solid Tumor | CD40、4-1BB、CTLA4，PD-1 |
| NCT03152318 | A Phase I Study of the Treatment of Recurrent Malignant Glioma With rQNestin34.5v.2, a Genetically Engineered HSV-1 Virus, and Immunomodulation With Cyclophosphamide | OV: rQNestin;  Other: Cyclophosphamide | Active, not recruiting | I | Malignant Glioma of Brain | - |
| NCT05205421 | A Single-Arm, Open-Label, Exploratory Study to Evaluate Safety and Efficacy of Oncolytic Virus Injection (RT-01) in Patients With Extensive-Stage Small Cell Lung Cancer | OV: RT-01 | Recruiting | I | Solid Tumor | - |
| NCT05228119 | A Single-Arm, Open-Label, Exploratory Study to Evaluate Safety and Efficacy of Oncolytic Virus Injection (RT-01) in Combination With PD-1 Inhibitor (Nivolumab) in Patients With Advanced Solid Tumors | OV: RT-01 | Recruiting | I | Solid Tumor | - |
| NCT05122572 | An Open-label Clinical Study to Evaluate the Safety and Efficacy of Intravenous With and Without Intratumoral Oncolytic Virus Injection (RT-01) Combined With or Without Immune Checkpoint Inhibitors (Nivolumab) in the Treatment of Patients With Advanced Solid Tumors | OV: RT-01 | Recruiting | I | Solid Tumor | - |
| NCT05136937 | An Open-Label, Dose Escalation Study of the Safety and Tolerability of Oncolytic Virus Injection(RT-01) When Administered Via Intratumoral Injection in Patients With Advanced Solid Tumors | OV: RT-01 | Recruiting | I | Solid Tumor | - |
| NCT05387226 | A Sing-Arm, Open Clinical Pharmacology Study of Intravenous Injection of Oncolytic Virus Injection (RT-01) in Patients With Relapsed or Refractory T-cell Lymphoma | OV: RT-01 | Not yet recruiting | I | T-cell Lymphoma | - |
| NCT05205408 | A Single-Arm, Open-Label, Dose Escalation Study to Evaluate Safety and Efficacy of Intratumoral Injection of Oncolytic Virus Injection (RT-01) in Patients With Advanced Solid Tumors | OV: RT-01 | Not yet recruiting | I | Solid Tumor | - |
| NCT00002960 | A Phase I Study in Patients With Peritoneal Carcinomatosis Using SCH 58500 (rAd/p53) Administered by Single Intraperitoneal Instillation | OV: SCH-58500 | Completed | I | Fallopian Tube Cancer，Metastatic Cancer，Ovarian Cancer，Primary Peritoneal Cavity Cancer | p53 |
| NCT00003880 | A Phase II/III Trial of Chemotherapy Alone Versus Chemotherapy Plus SCH 58500 in Newly Diagnosed Stage III Ovarian and Primary Peritoneal Cancer Patients With Greater Than or Equal to 0.5 cm and Less Than or Equal to 2 cm Residual Disease Following Surgery | OV: SCH-58500;  Other: Carboplatin, Paclitaxel | Terminated | II/III | Ovarian Cancer，Primary Peritoneal Cavity Cancer | p53 |
| NCT05361954 | A Phase 1b, Dose-Escalation Study of the Safety and Preliminary Efficacy of STI-1386 Oncolytic Virus in Patients With Relapsed or Refractory Solid Tumors | OV: STI-1386 | Not yet recruiting | I | Solid Tumor | PD-1、TGFBR2、IL12R |
| NCT00314925 | Phase I Dose-Escalation Study of Seneca Valley Virus (SVV-001), a Replication-Competent Picornavirus, in Patients With Advanced Solid Tumors With Neuroendocrine Features | OV: SVV-001 | Unknown | I | Carcinoid，Neuroendocrine | ANTXR1 |
| NCT04612504 | An Open-label Phase I/IIa Study to Evaluate the Safety, Tolerability and Efficacy of SynOV1.1 Recombinant Oncolytic Adenovirus Injection as Monotherapy or in Combination With Atezolizumab in Participants With Locally Advanced or Metastatic Hepatocellular Carcinoma | OV: SynOV1.1;  Other: Atezolizumab | Not yet recruiting | I/II | Hepatocellular Carcinoma | PD-L1，AFP |
| NCT04780217 | A Phase 1/2a Open-Label Dose Escalation and Dose Expansion Study of T3011 When Administered Via Intravenous Infusion as a Single Agent and in Combination With Pembrolizumab in Participants With Advanced or Metastatic Solid Tumors | OV: T3011;  Other: Pembrolizumab | Recruiting | I/II | Solid Tumor | PD-1、IL12R，PD-1 |
| NCT04301011 | A Phase 1/2a, Multicenter, Open-label Trial of TBio-6517, an Oncolytic Vaccinia Virus, Administered Alone and in Combination With Pembrolizumab, in Patients With Advanced Solid Tumors | OV: TBio-6517;  Other: Pembrolizumab | Recruiting | I/II | Solid Tumor | CTLA4、FLT3、IL12，PD-1 |
| NCT03921021 | Phase 2 Study of Telomelysin (OBP-301) in Combination With Pembrolizumab in Esophagogastric Adenocarcinoma | OV: Telomelysin | Recruiting | II | Esophagogastric Adenocarcinoma | TERT |
| NCT03294486 | Safety and Efficacy of the ONCOlytic VIRus Armed for Local Chemotherapy, TG6002/5-FC, in Recurrent Glioblastoma Patients | OV: TG6002;  Other: 5-flucytosine | Unknown | I/II | Glioblastoma，Brain Cancer | TYMS，FCU1 |
| NCT03724071 | A Phase I/IIa Study of TG6002 (VV TK-RR-FCU1) Administered by Intravenous (IV) Infusions in Combination With Oral Flucytosine (5-FC) in Patients With Advanced Gastro-intestinal (GI) Tumors. | OV: TG6002;  Other: 5-Flucytosine | Recruiting | I/II | Colorectal Neoplasm，Digestive System Neoplasm | FCU1，TYMS |
| NCT04194034 | A Dose-escalation and Phase IIa Study of TG6002 Plus Flucytosine in Patients With Unresectable Colorectal Cancer With Liver Metastases | OV: TG6002;  Other: Flucytosine | Recruiting | I/II | Colorectal Neoplasms | FCU1，TYMS |
| NCT04226066 | A Phase I/IIa Study to Evaluate the Safety, Tolerability and Pharmacokinetics Characteristics of Recombinant Oncolytic Vaccinia Virus Injection T601 as a Single Drug or in Combination With Oral Flucytosine (5-FC), in Patients With Advanced Malignant Solid Tumors | OV: TG601;  Other: Flucytosine | Recruiting | I/II | Advanced Malignant Solid Tumors | FCU1，TYMS |
| NCT04739046 | A Single Arm, Open, Exploratory Clinical Trial to Evaluate Efficacy and Safety for Combination Treatment of Replication Competent Adenovirus Double Suicide Gene Therapy(Theragene®,Ad5-yCD/mutTKSR39rep-ADP) and Radiation Therapy in Patients | OV: Theragene | Recruiting | II | Pancreas Cancer | - |
| NCT04695327 | A Phase 1, Open-Label, Dose-escalation Clinical Trial of Tumor Necrosis Factor Alpha and Interleukin-2 Coding Oncolytic Adenovirus (TILT-123) in Patients With Injectable Solid Tumors | OV: TILT-123 | Recruiting | I | Solid Tumor | IL2R、TNFR1、TNFR2 |
| NCT04217473 | A Phase 1, Open-Label, Dose-Escalation Clinical Trial of Tumor Necrosis Factor Alpha and Interleukin 2 Coding Oncolytic Adenovirus TILT-123 in Melanoma Patients Receiving Adoptive Cell Therapy With Tumor Infiltrating Lymphocytes | OV: TILT-123 | Recruiting | I | Metastatic Melanoma | IL2R、TNFR1、TNFR2 |
| NCT05222932 | A Phase I Open-Label, Dose-escalation Clinical Trial of Tumor Necrosis Factor Alpha and IL-2 Coding Oncolytic Adenovirus TILT-123 and Avelumab in Solid Tumor Patients (Melanoma and SCCHN) Refractory to or Progressing After Anti-PD(L)1 | OV: TILT-123;  Other: Avelumab | Recruiting | I | Melanoma，Head and Neck Squamous Cell Carcinoma | IL2R、TNFR1、TNFR2，PD-L1 |
| NCT05271318 | A Phase I Open-Label, Dose-escalation Trial of Tumor Necrosis Factor Alpha and Interleukin-2 Coding Oncolytic Adenovirus (TILT-123) in Combination With Pembrolizumab in Patients With Platinum Resistant or Refractory Ovarian Cancer | OV: TILT-123;  Other: Pembrolizumab | Recruiting | I | Platinum-refractory Ovarian Carcinoma，Platinum-resistant Ovarian Cancer，Platinum-Resistant Fallopian Tube Carcinoma，Platinum-Resistant Primary Peritoneal Carcinoma，Platinum-Refractory Fallopian Tube Carcinoma，Platinum-Refractory Primary Peritoneal Carcinoma | IL2R、TNFR1、TNFR2 |
| NCT04195373 | A Phase I Open-label, Safety Study of Intra-tumoral Application of TMV-018 in Combination With 5-FC or Anti-PD-1 Therapy in Patients With Tumors of the Gastrointestinal Tract | OV: TMV-018;  Other: Flucytosine, Anti-PD-1 | Terminated | I | Gastrointestinal Cancer | SCD |
| NCT03430687 | A Phase I Study of Intravesical Talimogene Laherparepvec for Non-Muscle Invasive Transitional Cell Carcinoma | OV: T-VEC | Terminated | I | Bladder Urothelial Carcinoma | - |
| NCT03458117 | A Phase I, Open Label, Single Arm, Single Centre Study to Evaluate Mechanism of Action of Talimogene Laherparepvec (T-VEC) in Locally Advanced Non-melanoma Skin Cancer | OV: T-VEC | Completed | I | Non-melanoma Skin Cancer，Basal Cell Carcinoma，Squamous Cell Carcinoma，Cutaneous Lymphoma，Merkel Cell Carcinoma | - |
| NCT00402025 | Targeted Delivery of OncoVEX^GM-CSF by Endoscopic Ultrasound (EUS)-Guided Fine Needle Injection (FNI) in Patients With Irresectable Pancreatic Cancer: A Pilot Multinational Experiment on Safety and Proof of Concept | OV: T-VEC | Completed | I | Pancreatic Cancer | - |
| NCT03663712 | A Phase I Trial of Talimogene Laherparepvec for the Treatment of Peritoneal Surface Malignancies | OV: T-VEC | Active, not recruiting | I | Stage IV Peritoneal Surface Dissemination From Gastrointestinal or Recurrent, Platinum-resistant Ovarian Cancer That Cannot be Completely Resected | - |
| NCT03086642 | Study of Talimogene Laherparepvec Administered Endoscopically for the Treatment of Locally Advanced or Metastatic Pancreas Cancer Refractory to at Least One Chemotherapy Regimen | OV: T-VEC | Active, not recruiting | I | Pancreatic Cancer | - |
| NCT03064763 | A Phase 1, Multi-center, Open-Label, Dose De-escalation Study to Evaluate the Safety and Efficacy of Talimogene Laherparepvec in Japanese Subjects With Unresectable Stage IIIB-IV Malignant Melanoma | OV: T-VEC | Active, not recruiting | I | Melanoma | - |
| NCT02756845 | A Phase 1, Multi-center, Open-label, Dose De-escalation Study to Evaluate the Safety and Efficacy of Talimogene Laherparepvec in Pediatric Subjects With Advanced Non Central Nervous System Tumors That Are Amenable to Direct Injection | OV: T-VEC | Active, not recruiting | I | Advanced Non CNS Tumors | - |
| NCT03555032 | A Phase I/II Study of the Safety and Efficacy of Talimogene Laherparepvec (T-VEC) Delivered by Intra-tumoural Injection in Combination With Isolated Limb Perfusion With Melphalan and Tumour Necrosis Factor-α in Patients With Advanced Extremity Tumours Including Metastatic Melanoma | OV: T-VEC | Completed | I/II | Melanoma and Sarcoma | - |
| NCT04427306 | Biomarker Analysis of Neoadjuvant Intralesional Therapy in High Risk Early Melanoma | OV: T-VEC | Recruiting | II | Melanoma | - |
| NCT04330430 | Neo-adjuvant T-VEC + Nivolumab Combination Therapy for Resectable Early Metastatic (Stage IIIB/C/D-IV M1a) Melanoma With Injectable Disease | OV: T-VEC | Recruiting | II | Melanoma Stage III，Melanoma Stage IV | -，PD-1 |
| NCT04065152 | Phase II Multicenter Study of Talimogene Laherparepvec in Classic or Endemic Kaposi Sarcoma - KAPVEC | OV: T-VEC | Recruiting | II | Kaposi Sarcoma | - |
| NCT02574260 | Phase 2 Extension Protocol for Extended Use of OncoVEX^GM-CSF for Eligible Patients Participating in Study 002/03: Study of the Efficacy, Safety and Immunogenicity of OncoVEX^GM-CSF in Patients With Stage IIIc and Stage IV Malignant Melanoma | OV: T-VEC | Completed | II | Melanoma | - |
| NCT02366195 | A Phase 2, Multicenter, Open-label, Single-arm Trial to Evaluate the Correlation Between Objective Response Rate and Baseline Intratumoral CD8+ Cell Density in Subjects With Unresected Stage IIIB to IVM1c Melanoma Treated With Talimogene Laherparepvec | OV: T-VEC | Completed | II | Melanoma | - |
| NCT02014441 | A Phase 2, Multicenter, Single-arm Trial to Evaluate the Biodistribution and Shedding of Talimogene Laherparepvec in Subjects With Unresected, Stage IIIB to IVM1c Melanoma | OV: T-VEC | Completed | II | Melanoma | - |
| NCT00289016 | A Phase II Study of the Efficacy, Safety and Immunogenicity of OncoVEX^GM-CSF in Patients With Stage IIIc and Stage IV Malignant Melanoma | OV: T-VEC | Completed | II | Melanoma | - |
| NCT02658812 | A Phase II Study Using Talimogene Laherparepvec for Inflammatory Breast Cancer (IBC) or Non-IBC Patients With Inoperable Local Recurrence | OV: T-VEC | Terminated | II | Malignant Chest Wall Neoplasm，Recurrent Breast Carcinoma，Recurrent Inflammatory Breast Carcinoma，Stage IV Breast Cancer AJCC v6 and v7，Stage IV Inflammatory Breast Carcinoma | - |
| NCT03921073 | A Phase II Study of Talimogene Laherparepvec (T-VEC) in the Treatment of Locally Advanced Cutaneous Angiosarcoma | OV: T-VEC | Active, not recruiting | II | Angiosarcoma of Skin | - |
| NCT03714828 | A Single Arm Phase 2 Study of Talimogene Laherparepvec in Patients With Cutaneous Squamous Cell Cancer | OV: T-VEC | Active, not recruiting | II | Squamous Cell Carcinoma，Skin Cancer，Keratoacanthoma，Cutaneous Tumor，Skin Cancer, Squamous Cell，Lesion Skin | - |
| NCT03256344 | A Phase 1b Study of Talimogene Laherparepvec in Combination With Atezolizumab in Subjects With Triple Negative Breast Cancer and Colorectal Cancer With Liver Metastases | OV: T-VEC;  Other: Atezolizumab | Completed | I | Metastatic Triple Negative Breast Cancer，Metastatic Colorectal Cancer | -，PD-L1 |
| NCT03300544 | A Phase I Study of Talimogene Laherparepvec (Talimogene Laherparepvec) With Neoadjuvant Chemotherapy and Radiation in Adenocarcinoma of the Rectum | OV: T-VEC;  Other: Capecitabine, Fluorouracil, Leucovorin, Oxaliplatin, Radiation | Active, not recruiting | I | Rectal Adenocarcinoma | - |
| NCT03088176 | A Phase 1b Trial of Talimogene Laherparepvec in Combination With Dabrafenib and Trametinib in Advanced Melanoma With an Activating BRAF Mutation | OV: T-VEC;  Other: Dabrafenib, Trametinib | Active, not recruiting | I | Melanoma，BRAF Gene Mutation | -，BRAF，MAP2K1、MAP2K2 |
| NCT03972046 | Neoadjuvant Use of Talimogene Laherparepvec and BRAF/MEK Inhibitor for Advanced Nodal BRAF Mutant Melanoma: A Pilot Study | OV: T-Vec;  Other: Dabrafenib, Trametinib | Terminated | II | Melanoma (Skin)，Melanoma Stage IIIb-IVM1a，Metastasis Skin，Tumor Skin，BRAF Gene Mutation | -，BRAF，MAP2K1、MAP2K2 |
| NCT01740297 | Phase 1b/2, Multicenter, Open-label Trial to Evaluate the Safety and Efficacy of Talimogene Laherparepvec and Ipilimumab Compared to Ipilimumab Alone in Subjects With Unresected, Stage IIIB-IV Melanoma | OV: T-VEC;  Other: Ipilimumab | Completed | I/II | Melanoma | CTLA4，- |
| NCT04185311 | A Phase 1 Study of Ipilumumab, Nivolumab and Talimogene Laherparepvec Preoperative Treatment of Localized Breast Cancer-deleted | OV: T-VEC;  Other: Ipilimumab, Nivolumab | Active, not recruiting | I | Breast cancer | CTLA4，PD-1，- |
| NCT03597009 | A Phase Ib/II Study of IV Nivolumab and Intrapleural Talimogene Laherparepvec for Patients With Malignant Pleural Effusion | OV: TVEC;  Other: Nivolumab | Terminated | I/II | Malignant Pleural Effusion，Metastatic Cancer，Lung Cancer | -，PD-1 |
| NCT02978625 | A Phase II Study of Talimogene Laherparepvec Followed by Talimogene Laherparepvec + Nivolumab in Refractory T Cell and NK Cell Lymphomas, Cutaneous Squamous Cell Carcinoma, Merkel Cell Carcinoma, and Other Rare Skin Tumors | OV: T-VEC;  Other: Nivolumab | Recruiting | II | Adenoid Cystic Skin Carcinoma，Adnexal Carcinoma，Anaplastic Large Cell Lymphoma, ALK-Negative，Anaplastic Large Cell Lymphoma, ALK-Positive，Apocrine Carcinoma，Cylindrocarcinoma，Digital Papillary Adenocarcinoma，Endocrine Mucin-Producing Sweat Gland Carcinoma，Extramammary Paget Disease，Extraocular Cutaneous Sebaceous Carcinoma，Hidradenocarcinoma，Keratoacanthoma，Malignant Sweat Gland Neoplasm，Merkel Cell Carcinoma，Microcystic Adnexal Carcinoma，NK-Cell Lymphoma, Unclassifiable，Papillary Adenocarcinoma，Porocarcinoma，Primary Cutaneous Mucinous Carcinoma，Recurrent Mature T-Cell and NK-Cell Non-Hodgkin Lymphoma，Recurrent T-Cell Non-Hodgkin Lymphoma，Refractory Anaplastic Large Cell Lymphoma，Refractory Mature T-Cell and NK-Cell Non-Hodgkin Lymphoma，Refractory Merkel Cell Carcinoma，Refractory Mycosis Fungoides，Refractory Primary Cutaneous T-Cell Non-Hodgkin Lymphoma，Refractory Skin Squamous Cell Carcinoma，Refractory T-Cell Non-Hodgkin Lymphoma，Sezary Syndrome，Signet Ring Cell Carcinoma，Skin Basal Cell Carcinoma，Skin Basosquamous Cell Carcinoma，Skin Squamous Cell Carcinoma，Spiradenocarcinoma，Squamoid Eccrine Ductal Carcinoma，Squamous Cell Carcinoma of Unknown Primary，Sweat Gland Carcinoma，Trichilemmal Carcinoma，Vulvar Squamous Cell Carcinoma | -，PD-1 |
| NCT03886311 | The TNT Protocol: A Phase 2 Study Using Talimogene Laherparepvec,Nivolumab and Trabectedin as First, Second/Third Line Therapy for Advanced Sarcoma, Including Desmoid Tumor and Chordoma | OV: T-VEC;  Other: Nivolumab, Trabectedin | Recruiting | II | Sarcoma | -，PD-1，MARCO、ABCB11 |
| NCT03554044 | A Phase 1b Study of Talimogene Laherparepvec (T-VEC) in Combination With Chemotherapy or Endocrine Therapy in Patients With Metastatic, Unresectable, or Locoregionally Recurrent HER2-negative Breast Cancer | OV: T-VEC;  Other: Paclitaxel, Nab paclitaxel, Gemcitabine, Carboplatin, Anastrozole, Exemestane, Fulvestrant, Letrozole, Tamoxifen | Recruiting | I | Anatomic Stage III Breast Cancer AJCC v8，Anatomic Stage IIIA Breast Cancer AJCC v8，Anatomic Stage IIIB Breast Cancer AJCC v8，Anatomic Stage IIIC Breast Cancer AJCC v8，Anatomic Stage IV Breast Cancer AJCC v8，Estrogen Receptor Positive，HER2/Neu Negative，Invasive Breast Carcinoma，Prognostic Stage III Breast Cancer AJCC v8，Prognostic Stage IIIA Breast Cancer AJCC v8，Prognostic Stage IIIB Breast Cancer AJCC v8，Prognostic Stage IIIC Breast Cancer AJCC v8，Prognostic Stage IV Breast Cancer AJCC v8，Recurrent Breast Carcinoma | Tubulin，-，Tubulin，RNR，-，CYP19A1，CYP19A1，ER-α、ER-β，CYP19A1，ER |
| NCT04163952 | A Phase 1 Study of Talimogene Laherparepvec and Panitumumab in Patients With Locally Advanced Squamous Cell Carcinoma of the Skin (SCCS) | OV: T-VEC;  Other: Panitumumab | Active, not recruiting | I | Locally Advanced Skin Squamous Cell Carcinoma，Metastatic Skin Squamous Cell Carcinoma，Recurrent Skin Squamous Cell Carcinoma | -，EGFR |
| NCT02626000 | A Phase 1b/3 Multicenter, Randomized, Trial of Talimogene Laherparepvec in Combination With Pembrolizumab for the Treatment of Subjects With Recurrent or Metastatic Squamous Cell Carcinoma of the Head and Neck | OV: T-VEC;  Other: Pembrolizumab | Completed | I | Carcinoma of the Head and Neck | PD-1，- |
| NCT02509507 | A Phase 1b/2, Multicenter, Open-label, Basket Trial to Evaluate the Safety of Talimogene Laherparepvec Injected Into Liver Tumors Alone and in Combination With Systemic Pembrolizumab in Phase 1b and to Evaluate the Efficacy and Safety of Intratumoral Talimogene Laherparepvec in Combination With Systemic Pembrolizumab to Treat Subjects With Advanced Solid Tumors in Phase 2 (MASTERKEY-318) | OV: T-VEC;  Other: Pembrolizumab | Active, not recruiting | I/II | Hepatocellular Carcinoma，Liver Metastases，Cutaneous or Subcutaneous Lymph Node，Liver Tumors | -，PD-1 |
| NCT03842943 | Neoadjuvant Combination Immunotherapy for Stage III Melanoma | OV: T-VEC;  Other: Pembrolizumab | Recruiting | II | Cutaneous Melanoma | PD-1，- |
| NCT03069378 | A Phase II Study of Talimogene Laherparepvec (T-VEC) Administered Concurrently With the Anti-PD1 Monoclonal Antibody Pembrolizumab in Patients With Metastatic and/or Locally Advanced Sarcoma | OV: T-VEC;  Other: Pembrolizumab | Recruiting | II | Sarcoma，Epithelioid Sarcoma，Cutaneous Angiosarcoma | - |
| NCT04068181 | Phase 2 Study of Talimogene Laherparepvec in Combination With Pembrolizumab in Subjects With Unresectable/Metastatic Stage IIIB-IVM1d Melanoma Who Have Progressed on Prior Anti PD-1 Based Therapy | OV: T-VEC;  Other: Pembrolizumab | Active, not recruiting | II | Melanoma | PD-1，- |
| NCT02965716 | A Phase II Study of Combining Talimogene Laherparepvec (T-VEC) (NSC-785349) and MK-3475 (Pembrolizumab) (NSC-776864) in Patients With Advanced Melanoma Who Have Progressed on Anti-PD1/L1 Based Therapy | OV: T-VEC;  Other: Pembrolizumab | Active, not recruiting | II | Melanoma | -，PD-1 |
| NCT02263508 | A Phase 1b/3, Multicenter, Trial of Talimogene Laherparepvec in Combination With Pembrolizumab (MK-3475) for Treatment of Unresectable Stage IIIB to IVM1c Melanoma (MASTERKEY-265) | OV: T-VEC;  Other: Pembrolizumab, Placebo | Recruiting | III | Melanoma | PD-1，- |
| NCT02819843 | A Phase II Randomized Trial of Intralesional Talimogene Laherparepvec (TALIMOGENE LAHERPAREPVEC) With or Without Radiotherapy for Cutaneous Melanoma, Merkel Cell Carcinoma, or Other Solid Tumors | OV: TVEC;  Other: Radiation | Active, not recruiting | II | Melanoma，Merkel Cell Carcinoma，Other Solid Tumors | - |
| NCT04599062 | Neoadjuvant Intralesional Injection of Talimogene Laherparepvec With Concurrent Preoperative Radiation in Patients With Locally Advanced Soft Tissue Sarcomas | OV: T-VEC;  Other: Radiation | Recruiting | I/II | Soft Tissue Sarcoma | - |
| NCT02923778 | A Phase 2 Study of Talimogene Laherparepvec (T-VEC) and Radiation in Localized Soft Tissue Sarcoma | OV: T-VEC;  Other: Radiation | Recruiting | II | FNCLCC Sarcoma Grade 2，FNCLCC Sarcoma Grade 3，Resectable Leiomyosarcoma，Resectable Liposarcoma，Resectable Soft Tissue Sarcoma，Resectable Undifferentiated Pleomorphic Sarcoma，Stage I Soft Tissue Sarcoma AJCC v7，Stage II Soft Tissue Sarcoma AJCC v7，Soft Tissue Sarcoma of the Trunk and Extremities | - |
| NCT02211131 | A Phase 2, Multicenter, Randomized, Open-label Trial Assessing the Efficacy and Safety of Talimogene Laherparepvec Neoadjuvant Treatment Plus Surgery Versus Surgery Alone for Resectable, Stage IIIB to IVM1a Melanoma | OV: T-VEC;  Other: Surgery | Completed | II | Melanoma | - |
| NCT01368276 | An Extension Protocol to Evaluate the Efficacy and Safety of Extended Use Treatment With OncoVEX^GM-CSF for Eligible Melanoma Patients Participating in Study 005/05 | OV: T-VEC;  Other:Granulocyte Macrophage Colony-Stimulating Factor (GM-CSF) | Recruiting | III | Melanoma | - |
| NCT00769704 | A Randomized Phase 3 Clinical Trial to Evaluate the Efficacy and Safety of Treatment With OncoVEX^GM-CSF Compared to Subcutaneously Administered GM-CSF in Melanoma Patients With Unresectable Stage IIIb, IIIc and IV Disease | OV: T-VEC;  Other:Granulocyte Macrophage Colony-Stimulating Factor (GM-CSF) | Recruiting | III | Melanoma | - |
| NCT02453191 | Neoadjuvant Intralesional Injection of Talimogene Laherparepvec With Concurrent Preoperative Radiation in Patients With Locally Advanced Soft Tissue Sarcomas | OV: T-VEC; Radiation | Active, not recruiting | I/II | Soft Tissue Sarcoma | - |
| NCT01161498 | A Phase 3 Randomized Trial of Concurrent Cisplatin & Radiotherapy With Or Without ONCOVEX^GM-CSF In Previously Untreated Patients With Locally Advanced Squamous Cell Carcinoma Of The Head And Neck | OV: T-VEC; Radiation, Cisplatin | Recruiting | III | Squamous Cell Carcinoma，Head and Neck Cancer | - |
| NCT04521621 | A Phase 1b/2 Clinical Study of Intratumoral Administration of V937 in Combination With Pembrolizumab (MK-3475) in Participants With Advanced/Metastatic Solid Tumors | OV: V937;  Other: Pembrolizumab | Active, not recruiting | I/II | Neoplasm Metastasis | ICAM1、CD55，PD-1 |
| NCT04152863 | A Phase 2, Randomized Clinical Study of Intravenous or Intratumoral Administration of V937 in Combination With Pembrolizumab (MK-3475) Versus Pembrolizumab Alone in Participants With Advanced/Metastatic Melanoma | OV: V937;  Other: Pembrolizumab | Active, not recruiting | II | Advanced/Metastatic Melanoma | ICAM1、CD55，PD-1 |
| NCT01436968 | A Randomized Controlled Trial of ProstAtak® as Adjuvant to Up-front Radiation Therapy For Localized Prostate Cancer | OV: Valacyclovir;  Other: Placebo | Recruiting | III | Prostate Cancer | DNA Pol，- |
| NCT03284268 | Phase I Study, Single Site, Open Label With Dose Escalation, for Evaluate Safety and the Oncolitic Adenovirus VCN-01 Activity in Patients With Refractory Retinoblastoma | OV: VCN-01 | Recruiting | I | Retinoblastoma | - |
| NCT03799744 | A Phase I Study to Evaluate the Safety, Tolerability, and Efficacy of VCN-01 in Combination With Durvalumab (MEDI4736) in Subjects With Recurrent/ Metastatic Squamous Cell Carcinoma of the Head and Neck | OV: VCN-01;  Other: Durvalumab | Active, not recruiting | I | Head and Neck Neoplasms，Carcinoma, Squamous Cell of Head and Neck，Metastasis，Recurrence | PD-L1，- |
| NCT02045589 | A Phase I, Multicenter, Open-label, Dose Escalation Study of Intratumoral Injections of VCN-01 Oncolytic Adenovirus With Intravenous Gemcitabine and Abraxane® in Advanced Pancreatic Cancer | OV: VCN-01;  Other: Gemcitabine, Abraxane | Completed | I | Pancreatic Adenocarcinoma | - |
| NCT02045602 | A Phase I, Multicenter, Open-label, Dose Escalation Study of Intravenous Administration of VCN-01 Oncolytic Adenovirus With or Without Gemcitabine and Abraxane® in Patients With Advanced Solid Tumors | OV: VCN-01;  Other: Gemcitabine, Abraxane | Completed | I | Pancreatic Adenocarcinoma | -，RNR，Tubulin |
| NCT05057715 | Phase 1 Trial of Human Chimeric Antigen Receptor Modified T Cells (huCART-meso) Administered in Combination With VCN-01 in Patients With Pancreatic and Serous Epithelial Ovarian Cancer | OV: VCN-01;  Other: huCART-meso Cells | Recruiting | I | Pancreatic Cancer，Serous Ovarian Cancer | MSLN，- |
| NCT04806464 | A Dose Ascending, Open Phase I Clinical Study to Evaluate the Safety, Tolerability , Pharmacokinetics Characteristics and Preliminary Effectiveness of VG161 in Subjects With Advanced Primary Liver Cancer | OV: VG161 | Recruiting | I | Primary Liver Cancer | IL12R、IL2/15Rβγc、PD-L1 |
| NCT04758897 | A Dose Ascending, Open Phase I Clinical Study to Evaluate the Safety, Tolerability and Pharmacokinetics Characteristics of VG161 in Subjects With Advanced Malignant Solid Tumors | OV: VG161 | Recruiting | I | Solid Tumor | IL12R、IL2/15Rβγc、PD-L1 |
| NCT05223816 | An Open-Label, Multiple-Center, Phase IIa/IIb Clinical Trial to Evaluate the Efficacy, Safety and Tolerability of VG161 in the Treatment of Patients With Hepatocellular Carcinoma or Intrahepatic Cholangiocarcinoma | OV: VG161 | Recruiting | II | Hepatocellular Carcinoma，Intrahepatic Cholangiocarcinoma | IL12R、IL2/15Rβγc、PD-L1 |
| NCT05162118 | Clinical Trial to Evaluate the Safety, Tolerability and Preliminary Efficacy of VG161 in Combination With Nivolumab in Patients With Advanced Pancreatic Cancer | OV: VG161;  Other: Nivolumab | Recruiting | I/II | Advanced Pancreatic Cancer | IL12R、IL2/15Rβγc、PD-L1 |
| NCT05477849 | A Dose Escalation, Open-label Phase I Clinical Study to Evaluate the Safety, Tolerability, Pharmacokinetics and Biologic Effect of VG2025 in the Treatment of Patients With Advanced Malignant Solid Tumors | OV: VG2025 | Recruiting | I | Solid Tumor | IL12、IL15 |
| NCT05266612 | A Phase I Clinical Study to Evaluate the Safety, Tolerability, Pharmacokinetics and Biologic Effect of VG2025 in Subjects With Advanced Malignant Solid Tumors | OV: VG2025 | Not yet recruiting | I | Solid Tumor | IL12、IL15 |
| NCT03647163 | Ph I/II Trial of Systemic Administration of VSV-IFNβ-NIS in Combination With Pembrolizumab, With Expansion Cohorts in Patients With Refractory NSCLC and NEC | OV: VSV-IFNβ-NIS;  Other: Pembrolizumab | Recruiting | I/II | Solid Tumor | IFNB1 |
| NCT02923466 | Phase 1 Trial of Vesicular Stomatitis Virus Genetically Engineered to Express NIS and Human Interferon Beta (VSV-IFNβ-NIS) Monotherapy and in Combination With Avelumab, in Patients With Refractory Solid Tumors | OV: VSV-IFNβ-NIS;  Other: VSV-IFNβ-NIS, Avelumab | Completed | I | Malignant Solid Tumour | IFNB1 |
| NCT04291105 | Phase 2 Trial of Voyager V1 in Combination With Cemiplimab in Patients With Hepatocellular Carcinoma, Non-Small Cell Lung Cancer, Melanoma or Endometrial Carcinoma | OV: VV1;  Other: Cemiplimab, Ipilumumab | Recruiting | II | Melanoma，Non Small Cell Lung Cancer | IFNB1，PD-1 |
| NCT00574977 | A Phase I Dose-escalation Trial of vvDD-CDSR (Double-deleted Vaccinia Virus Plus CD/ SMR) Administered by Intratumoral Injection or Intravenous Injection | OV: vvDD-CDSR | Completed | I | Melanoma，Breast Cancer，Head and Neck Squamous Cell Cancer，Liver Cancer，Colorectal Cancer，Pancreatic Adenocarcinoma | - |
| NCT01533194 | A Phase 1 Study of Reolysin Alone in Patients With Relapsed or Refractory Multiple Myeloma | OV: Wild-type Reovirus | Completed | I | Light Chain Deposition Disease，Refractory Plasma Cell Myeloma | - |
| NCT00651157 | A Phase II Trial of Intravenous Administration of Reovirus Serotype 3 - Dearing Strain (Reolysin®) in Patients With Metastatic Melanoma | OV: Wild-type reovirus | Completed | II | Melanoma | - |
| NCT01280058 | A 2-arm Randomized Phase II Study of Carboplatin, Paclitaxel Plus Reovirus Serotype-3 Dearing Strain (Reolysin) vs. Carboplatin and Paclitaxel in the First Line Treatment of Patients With Recurrent or Metastatic Pancreatic Cancer | OV: Wild-type Reovirus,  Other: Carboplatin, Paclitaxel | Completed | II | Pancreatic Acinar Cell Carcinoma，Pancreatic Ductal Adenocarcinoma，Recurrent Pancreatic Carcinoma，Stage IV Pancreatic Cancer | - |
| NCT02514382 | A Phase 1b Study of REOLYSIN® (Reovirus Serotype 3 - Dearing Strain) Combined With Standard Doses of Bortezomib and Dexamethasone in Patients With Relapsed/Refractory Multiple Myeloma | OV: Wild-type Reovirus;  Other: Bortezomib, Dexamethasone | Active, not recruiting | I | Recurrent Plasma Cell Myeloma，Refractory Plasma Cell Myeloma | - |
| NCT01240538 | A Phase 1 Dose Escalation Study of Reolysin, a Replication Competent Reovirus, in Pediatric Patients With Relapsed or Refractory Solid Tumors | OV: Wild-type reovirus;  Other: Cyclophosphamide | Completed | I | Unspecified Childhood Solid Tumor | - |
| NCT03723915 | A Phase 2 Study of Pembrolizumab in Combination With Pelareorep in Patients With Advanced Pancreatic Adenocarcinoma | OV: Wild-type Reovirus;  Other: Pembrolizumab | Terminated | II | Pancreatic Adenocarcinoma | PD-1，- |
| NCT02444546 | Phase 1 Study of Replication Competent Reovirus (Reolysin®) in Combination With GM-CSF in Pediatric Patients With Relapsed or Refractory Brain Tumors | OV: Wild-type Reovirus;  Other: Sargramostim | Active, not recruiting | I | Childhood Astrocytoma | -，CSF2R |
| NCT00390299 | Phase I Trial of a Measles Virus Derivative Producing CEA (MV-CEA) in Patients With Recurrent Glioblastoma Multiforme (GBM) | OV:MV-CEA;  Other: Surgery | Completed | I | Anaplastic Astrocytoma，Anaplastic Oligodendroglioma，Mixed Glioma，Recurrent Glioblastoma | CEA |
| NCT04370587 | A Phase 1/2a, Open-Label, Dose Escalation and Expansion Study of the Safety and Tolerability of T3011 Administered Via Intratumoral Injection as a Single Agent and in Combination With Intravenous Pembrolizumab in Patients With Advanced or Metastatic Solid Tumors | OV: T3011;  Other: Pembrolizumab | Recruiting | I/II | Solid Tumor | PD-1、IL12R，PD-1 |
| NCT02779855 | A Phase 1/2 Study of Talimogene Laherparepvec in Combination With Neoadjuvant Chemotherapy in Triple Negative Breast Cancer | OV: T-VEC;  Other: Paclitaxel | Active, not recruiting | I/II | Breast Cancer，Ductal Carcinoma，Invasive Breast Carcinoma，Invasive Ductal Breast Carcinoma | -，Tubulin |
